# The tRNA identity landscape for aminoacylation and beyond

**DOI:** 10.1093/nar/gkad007

**Published:** 2023-02-06

**Authors:** Richard Giegé, Gilbert Eriani

**Affiliations:** Architecture et Réactivité de l’ARN, UPR9002 Centre National de la Recherche Scientifique, Université de Strasbourg, Institut de Biologie Moléculaire et Cellulaire, 2 allée Konrad Roentgen, 67084 Strasbourg, France; Architecture et Réactivité de l’ARN, UPR9002 Centre National de la Recherche Scientifique, Université de Strasbourg, Institut de Biologie Moléculaire et Cellulaire, 2 allée Konrad Roentgen, 67084 Strasbourg, France

## Abstract

tRNAs are key partners in ribosome-dependent protein synthesis. This process is highly dependent on the fidelity of tRNA aminoacylation by aminoacyl-tRNA synthetases and relies primarily on sets of identities within tRNA molecules composed of determinants and antideterminants preventing mischarging by non-cognate synthetases. Such identity sets were discovered in the tRNAs of a few model organisms, and their properties were generalized as universal identity rules. Since then, the panel of identity elements governing the accuracy of tRNA aminoacylation has expanded considerably, but the increasing number of reported functional idiosyncrasies has led to some confusion. In parallel, the description of other processes involving tRNAs, often well beyond aminoacylation, has progressed considerably, greatly expanding their interactome and uncovering multiple novel identities on the same tRNA molecule. This review highlights key findings on the mechanistics and evolution of tRNA and tRNA-like identities. In addition, new methods and their results for searching sets of multiple identities on a single tRNA are discussed. Taken together, this knowledge shows that a comprehensive understanding of the functional role of individual and collective nucleotide identity sets in tRNA molecules is needed for medical, biotechnological and other applications.

## INTRODUCTION

**Figure 1.**
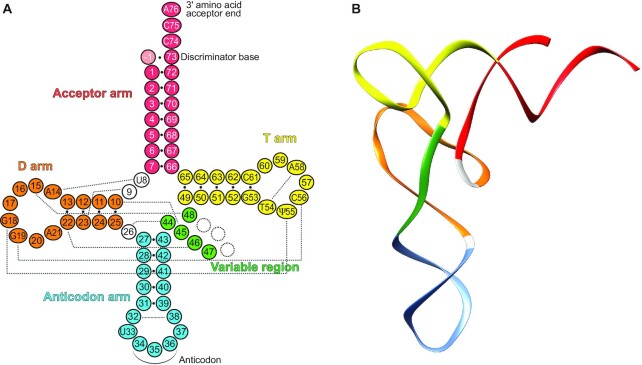
Cloverleaf folding of tRNA and its three-dimensional L-shaped organization. The color code highlights the structural domains in tRNA. (**A**) The standard cloverleaf structure of cytosolic tRNAs and the conventional numbering system are used. Conserved nucleotides are explicitly indicated. The variable region (nucleotides 44–48) encompasses the long extra arm of tRNA^Leu^, tRNA^Ser^ and tRNA^Tyr^. The · symbol indicates Watson–Crick base pairings (including G·U pairs); dotted gray lines indicate other pairings important for tRNA L-shaped architecture. (**B**) Three-dimensional L-shaped structure of tRNA^Phe^ (1ehz) showing the folding of the different arms.

tRNAs are the adapters that decode mRNAs into proteins in the ribosome-dependent translation apparatus, but this classical vision is expanding since tRNAs act in many biological processes (1–3). However, translation remains the cornerstone relying mainly on the fidelity of tRNA aminoacylation by aminoacyl-tRNA synthetases (aaRSs). How synthetases achieve fidelity/specificity was perhaps the first protein–RNA recognition problem to be seriously investigated. It led to the concept of tRNA identity, which refers to the amino acid ligated at the 3′ end (4,5). Currently, this issue is supported by a robust theoretical and experimental background. Due to their interaction with multiple components of the translation machinery, tRNAs have undergone significant constraints on their primary and secondary structures during evolution to retain structural similarity and the ability to interact with the ribosome-dependent translation machinery (Figure 1) (6). Early studies in the 1970s pinpointed the importance of the acceptor end and anticodon of tRNAs for recognition by aaRSs (7,8). It was also found that the specificity of aaRSs for amino acid activation and aminoacylation of tRNA is rather low (in other words, aaRSs catalyze amino acid misactivation and mischarging of tRNAs) and that accurate tRNA charging relies more on kinetic effects than on discrimination among cognate and non-cognate tRNAs through binding affinity (9). In addition, it was found that the binding of tRNAs to aaRSs, followed by the correct aminoacylation of tRNA, relies on a limited number of nucleotides called ‘identity elements’ supporting the ‘RNA operational code’ theory, associated with the idea of a second genetic code (10). The G_3_·U_70_ base pair in *Escherichia coli* tRNA^Ala^ was the first identity determinant experimentally validated (11,12). This pioneering result was followed by the characterization of determinant sets that specify the identity of all standard tRNA specificities, mainly in the tRNAs of *E. coli* and *Saccharomyces cerevisiae*. These early results were obtained mainly by *in vitro* methods and less often by *in vivo* methods that also measure the functional importance and strength of determinants (4,13). Two decades later, numerous results have enriched knowledge in the field of tRNA identity, shedding new light on the importance of the fidelity of the aminoacylation reaction. A full analysis incorporating these features and novelties is presented here.

## IDENTITY ELEMENTS FOR tRNA AMINOACYLATION IN STANDARD CYTOSOLIC SYSTEMS

### Positive identity elements in standard cytosolic tRNAs

#### General considerations

The panel of validated aminoacylation identity elements (nucleotides and structural features) also called ‘determinants’ has been substantially enriched over the last two decades. Typically, identification of determinants is achieved by comparing the aminoacylation capacities of mutant and native tRNAs determined by measuring their catalytic efficiencies which are related to the *k*_cat_/*K*_M_ ratios, where *k*_cat_ is the catalytic rate constant and *K*_M_ is the Michaelis constant representing an approximation of the inverse of the tRNA’s affinity for an aaRS. Comparison of the aminoacylation capacities of mutant and native tRNAs defines the ‘loss’ parameter ‘L’ = (*k*_cat_/*K*_M_)_native_/(*k*_cat_/*K*_M_)_mutant_ which reflects the loss of catalytic efficiency of the mutated tRNA. An ‘L’ <10 is generally considered a minor effect, while a larger ‘L’ >1000 is considered a major effect, with intermediate values being considered intermediate effects (4). Additionally, for *E. coli* tRNAs, strengths are also determined by tRNA suppressor-based genetic assays. However, despite its great potential, the *in vitro* SELEX approach that allows selection of aminoacylated tRNAs from a pool of randomized sequences is seldomly used (see below for Asp and Phe identities) (13,14). With the increasing number of studies in different organisms, the diversity of identity elements within each system has become apparent, challenging the idea of universal identity rules. The identity of mitochondrial tRNAs (mt-tRNAs) and atypical or standard tRNAs charged by aaRSs is covered in dedicated sections. Due to the abundance of literature covering the subject, some older references will only be cited sparingly; for additional references, see (4).

**Figure 2.**
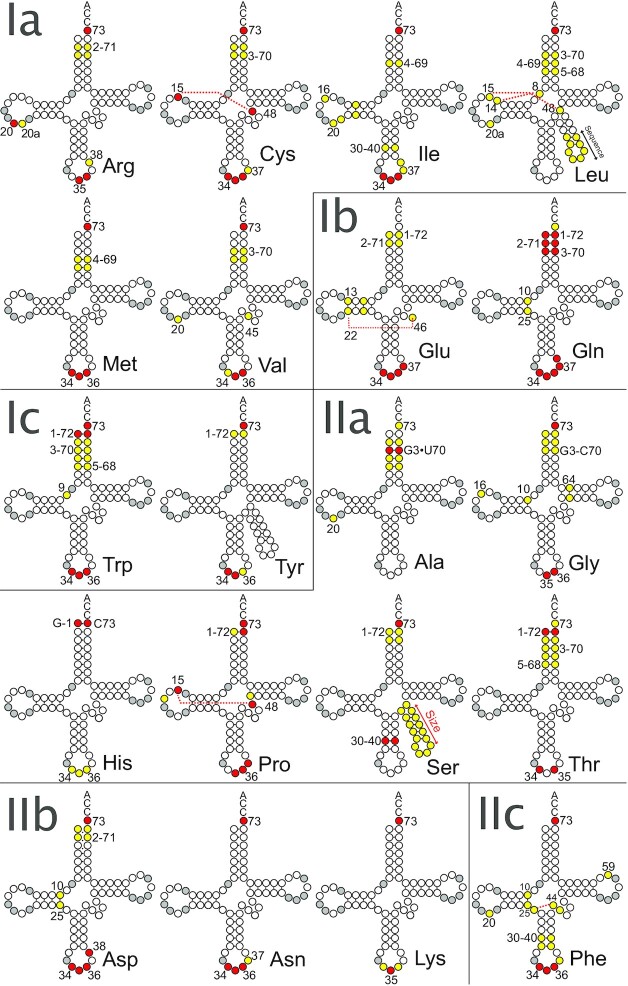
Schematic representation of the distribution of identity elements across the 20 isoacceptor tRNA families. The tRNAs are presented by class and subclass of aaRSs. Invariant positions are represented in gray. The positions in red are the conserved or nearly conserved strong identity elements. Positions in yellow are weak determinants or determinants not conserved in the three domains of life. The red dashed lines indicate tertiary interactions involved in identity. For tRNA^Leu^, the yellow double arrow indicates the low importance of the sequence elements in the extra arm; for tRNA^Ser^, the red double arrow indicates that the size rather than the sequence of the extra arm is the major identity element.

Below, the positive identity elements for tRNA aminoacylation, validated by functional assays, are shown in Table 1 and schematized in Figure 2. The identity determinants are discussed following the classification into classes and subclasses of aaRSs, namely subclass Ia (Arg-, Cys-, Ile-, Leu-, Met- and ValRS), subclass Ib (Glu- and GlnRS), subclass Ic (Trp- and TyrRS), subclass IIa (Ala-, Gly-, His-, Pro-, Ser- and ThrRS), subclass IIb (Asp-, Asn- and LysRS) and subclass IIc (PheRS). This classification is based on common structural characteristics, notably the oligomeric status of aaRSs, mainly monomeric for class I aaRSs and dimeric for class II aaRSs, which are considered among the most ancient in evolution (15). However, in some tRNA families, the oligomeric status of aaRSs is not conserved. Some class Ia Leu- and MetRSs and all class Ic Trp- and TyrRSs are dimers. Class IIa GlyRSs are dimers in *Eukarya* and *Archaea* and either dimers or heterotetramers in *Bacteria*. Tetrameric AlaRSs, GlyRSs and PheRSs are pseudodimers (15,16).

**Table 1.** Positive identity determinants for aminoacylation experimentally characterized in tRNAs from *Bacteria*, *Eukarya* (cytosol) and *Archaea*

tRNA families		tRNA domains		
		Acceptor branch	Core region	Anticodon branch
**Arg** ^a^	(Bac)	A/G_73_, U_73_, G_2_–C_71_, C_3_–G_70_	A_20_	C_35_, U/G_36_
	(Euk)	—_73_^b^	U_20_^c^, C_20a_, U_20a_	A/C_35_, A/U/G_36_, A_38_
	(Arc)	…	…	…
**Cys**	(Bac)	U_73_, G_2_–C_71_, C_3_–G_70_	TI^d^	G_34_, C_35_, A_36_
	(Euk)	U_73_	G_15_:G_48_, A_13_:A_22_	G_34_, C_35_, A_36_
	(Arc)	U_73_	G_15_, A_47_	G_34_, C_35_, A_36_, G_37_^c^
**Ile**	(Bac)	A_73_, C_4_–G_69_	U_12_–A_23_, G_16_, U_20_^c^, U_21_^c^	C_29_–G_41_, C/G_34_^c^, A_35_, U_36_, A_37_^c^, A_38_
	(Euk)	…	…	N_34_^c^, A_35_, U_36_
	(Arc)	…	…	…
**Leu** ^a^	(Bac)	A_73_	TI^d^, A_20a_, largeVR^e^	…
	(Euk)	A_73_, C_3_–G_70_, A_4_–U_69_, G_5_–C_68_	largeVR^e^, U_8_:A_14_	…
	(Arc)	A_73_	largeVR^e^	…
**Met** ^f^	(Bac)	A_73_, (G_2_–C_71_) (C_3_–G_70_) U_4_–A_69_, A_5_–U_68_	…	(C_32_) (U_33_) C_34_, A_35_, U_36_ (A_37_)
	(Euk)	A_73_	TI^d^	C_34_, A_35_, U_36_
	(Arc)	…	…	…
**Val**	(Bac)	A_73_, G_3_–C_70_, U_4_–A_69_	…	A_35_, C_36_
	(Euk)	A_73_	…	A_35_
	(Arc)	…	…	G_34_, A_35_, C_36_
**Glu**	(Bac)	–_73_^b^, G_1_–C_72_, U_2_–A_71_	TI^d^, Δ_47_	U_34_^c^, U_35_, A_37_, C_36_
	(Euk)	…	…	…
	(Arc)	…	…	…
**Gln**	(Bac)	G_73_, U_1_–A_72_, G_2_–C_71_, G_3_–C_70_	G_10_	U_34_^c^, U_35_, G_36_, A_37_, U_38_
	(Euk)	…	…	…
	(Arc)	…	…	…
**Trp**	(Bac)	G_73_, A_1_–U_72_, G_2_–C_71_, G_3_–C_70_, G_4_–C_69_, G_5_–C_68_^g^	A_9_	C_34_, C_35_, A_36_
	(Euk)	A_73_, G_1_–C_72_, U_5·_G_68_^i^/G_5_–C_68_	…	C_34_, C_35_, A_36_
	(Arc)	A_73_, G_1_–C_72_, G_2_–C_71_	…	C_34_, C_35_, A_36_
**Tyr**	(Bac)	A_73_	…	G_34_^c^, U_35_
	(Euk)	A_73_, C_1_–G_72_	…	G_34_, U_35_^c^
	(Arc)	A_73_, C_1_–G_72_	…	G_34_^c^, U_35_, A_36_
**Ala**	(Bac)	A_73_, G_2_–C_71_, G_3_·U_70_, G_4_–C_69_	G_20_	…
	(Euk)	G_3–_U_70_	…	…
	(Arc)	…	…	…
**Gly**	(Bac)	U_73_, G_1_–C_72_, C_2_–G_71_, G_3_–C_70_	…	C_35_, C_36_
	(Euk)	A/U_73_, G_1_–C_72_, C_2_–G_71_, G_3_–C_70_	(G_10_:Y_25_):G_45_	C_35_, C_36_
	(Arc)	A_73_, C_2_–G_71_, G_3_–C_70_	…	C_35_, C_36_
**His**	(Bac)	G_-1_, C_73_	…	G_34_, U_35_, G_36_
	(Euk)	G_-1_, A_73_	…	G_34_, U_35_
	(Arc)	—_-1_^b^, C_73_	C_50_–G_64_	G_29_–C_41_
**Pro**	(Bac)	A_73_, G_72_	G_15_:C_48_, U_17a_, G_49_	G_35_, G_36_, G_37_
	(Euk)	C_73_	…	G_35_, G_36_
	(Arc)	A_73_, G_1_–C_72_, G_2_–C_71_, G_3_–C_70_	…	G_35_, G_36_
**Ser**	(Bac)	G_73_^h^, C_72_, G_2_–C_71_, R_4_–Y_69_	C_11_–G_24_; largeVR^g^	G_30_–C_40_
	(Euk)	G_73_	largeVR^e^	…
	(Arc)	…	…	…
**Thr**	(Bac)	G_1_–C_72_, C_2_–G_71_, G_4_–C_69_, G_5_–C_68_	…	G_34_, G_35_, U_36_
	(Euk)	U_73_, G_1_–C_72_, U_3_–A_70_, G_5_–C_68_	…	G_35_, U_36_
	(Arc)	—_73_^b^/U_73_^h^, G_1_–C_72_, C_2_–G_71_, C_3_–U_70_	…	G_34_, U_35_, C_36_
**Asp**	(Bac)	G_73_	G_10_	G_34_^c^, U_35_, C_36_, C_38_
	(Euk)	G_73_	G_10_·U_25_	G_34_, U_35_, C_36_
	(Arc)	…	…	…
**Asn**	(Bac)	G_73_	…	G_34_, U_35_, U_36_, A_37_^c^
	(Euk)	…	…	…
	(Arc)	…	…	…
**Lys**	(Bac)	A_73_	…	U_34_, U_35_, U_36_
	(Euk)	…	…	…
	(Arc)	…	…	…
**Phe**	(Bac)	A_73_	C_10_–G_25_, U_20_^c^, U_45_, U_59_	A_26_:G_44_, G_34_, A_35_, A_36_
	(Euk)	A_73_	G_20_	G_34_, A_35_, A_36_, A_37_
	(Arc)	A_73_	C_13_–G_22_, G_20_	G_34_, A_35_, A_36_

The 20 tRNA families designated by their amino acid identity are displayed according to the class and subclass of their corresponding aaRSs. The position of the determinants in the sequence of the tRNA acceptor branch, the core region and the anticodon branch is shown, with standard numbering.

Bac, *Bacteria*; Euk, *Eukarya*; Arc, *Archaea*; N, nucleos/tide; R–Y or Y–R (with R for purine and Y for pyimidine), Watson–Crick pairs; G·U, non-Watson-Crick pair; modified residues are shown in standard abbreviations; TI, tertiary interaction (with atypical N:N pairing); Δ, missing residue or domain; VR, variable region; …, no data; n.d., not determined.

^a^Insertion of an additional N in the D loop of some tRNA isoacceptors; ^b^position not involved in identity; ^c^modified Ns; ^d^specific N as determinants in TIs; ^e^specific N as determinants in large VRs; ^f^determinants of *E. coli* initiator tRNA^Met^ are given in parentheses; ^g^U_5_–G_68_ is determinant in *Homo sapiens* and G_5_–C_68_ in *B. subtilis* tRNA^Trp^; ^h^U_73_ is determinant in *Haloferax volcanii* tRNA^Thr^ but not in *A. pernix* tRNA^Thr^.

Due to the degeneracy of the genetic code, isoacceptor tRNAs can have different anticodons, for example up to six for tRNA^Arg^, tRNA^Leu^ and tRNA^Ser^, but only one for tRNA^Met^ and tRNA^Trp^. This results in a variable distribution of identity elements in the tRNA structure, with or without selection of the anticodon as a main element. Overall, determinants in bacterial and eukaryal tRNAs are well documented, in contrast to poorly known determinants in archaeal tRNAs (Table 1). In the **Appendix** section at the end, we provide a list of definitions of common terms used throughout the review.

#### Conservation and diversity of identity sets

##### Identity sets recognized by subclass Ia aminoacyl-tRNA synthetases

###### Arginine identity

The arginine identity is mainly defined by A_20_ in the variable pocket of the D loop, C_35_ and U/C_36_ in the anticodon and discriminator base A/G_73_ (17–21). The arginine identity set was enriched by the addition of U_20a_ and A_38_ elements in plant tRNAs^Arg^ (22). A_20_ is the main determinant of arginylation by *E. coli* ArgRS, as shown with a panel of *E. coli* and plant tRNA^Arg^ isoacceptors, despite variations in their frameworks (22). This is unique among the 20 tRNA families and is supported in the structures of the tRNA^Arg^:ArgRS complexes (23).

In contrast, position 20 in the D loop of *S. cerevisiae* tRNA^Arg^ isoacceptors participates only marginally in identity, while C_35_ followed by G_36_ or U_36_ in the anticodon loop are the prominent determinants (24). Here, arginine identity is defined by two distinct combinations of determinants in the anticodon loop of the four yeast tRNA^Arg^ isoacceptors. Surprisingly, the arginine identity in yeast is related to that of aspartate and does not require the participation of the discriminator base at position 73. The cryptic aspartate identity was carefully studied in the minor tRNA_4_^Arg(CCG)^ in which a specificity switch to aspartate was obtained with only the two mutations C_38_ and G_73_, thereby allowing discrimination between the three major tRNA_1–3_^Arg^ and the minor tRNA_4_^Arg^ isoacceptor (25). It is worth highlighting the importance of position 20 in controlling species specificity (A_20_ in *E. coli* and C_20_ in yeast) (26) and the association of A_20_ and C_35_ in hamster (27). The crystal structures of the yeast and *Thermus thermophilus* tRNA^Arg^:ArgRS complexes (28,29) show how D_20_ and C_20a_ in yeast and A_20_ in *T. thermophilus* control arginine identity. Finally, a comparative search of arginine identity elements performed in two related plant taxa, soybean and jack bean, concluded that the main arginine determinants are also A_20_ and C_35_ in plants, mammals and *E. coli*, but species variability occurs at other positions (22).

###### Cysteine identity

The discriminator U_73_ and the anticodon GCA are major identity determinants in *E. coli* and yeast tRNA^Cys^, although minor determinants occur in the yeast accepting stem and their strength is variable (30–32). The discriminator U_73_ alone is sufficient to confer cysteine identity to minihelices (33), suggesting an ancestral role in the evolution of cysteine identity. In addition, the atypical 15–48 *trans*-Watson–Crick Levitt pair (G_15_–G_48_ instead of R_15_–Y_48_) in the core region of *E. coli* tRNA^Cys^ is a strong determinant for aminoacylation (34). This atypical pair affects both the structure and function of *E. coli* tRNA^Cys^ (34,35) and is conserved in a few bacterial species, such as *Haemophilus influenzae* tRNA^Cys^ (36). In *Methanosarcina mazei*, one of the few methanogenic *Archaea* encoding CysRS, serylation of tRNA^Cys^ requires m^1^G_37_ that is also a serylation determinant for SepRS (37).

###### Isoleucine identity

The identity elements of the isoleucine system were among the first known and include strong determinants in the discriminator and anticodon positions (38–40). In *E. coli*, minor determinants in stem regions of tRNA^Ile^ (C_4_–G_69_, U_12_–G_23_ and C_29_–G_41_) (41) and discrete determinants in the D loop (G_16_, D_20_ and D_21_) are crucial for isoleucylation and editing (42,43). The determinants in the anticodon loop are of interest because of the closely related Ile (AUU, AUC and AUA) and Met (AUG) codons and the frequent modifications at wobble position 34. In *E. coli*, the minor tRNA_2_^Ile^ with an anticodon mimicking the CAU anticodon of tRNA^Met^ carries a lysine-substituted C_34_ named lysidine (L or k^2^C) which confers the ability to decode isoleucine AUA codons (38). This modified L_34_ residue in *E. coli* tRNA_2_^Ile^ is an isoleucine identity determinant since its replacement by unmodified C impairs isoleucine-accepting activity and surprisingly confers methionine activity. In contrast, it is an antideterminant that prevents the decoding of the methionine AUG codon and its recognition by MetRS (38). This dual property is likely to be generalized, as lysidine or a lysidine mimic is found at position 34 in other *Bacteria*, such as in *Lactobacilli* tRNAs^Ile^ (44,45) and in *Archaea* where the modified base is called agmatidine (or C+) (46). In *Eukarya*, I_34_ in the anticodon of yeast tRNA^Ile^ is also a determinant (47). At position 37, t^6^A_37_ is a strong determinant for tRNA isoleucylation by bacterial-type but not eukaryal-type IleRSs (48) except human cyto-IleRS (and IleRSs in related mammalian taxa) (49). In Table 2, we have compiled the currently known post-transcriptional modifications that play a role in the expression of tRNA identity (50).

**Table 2.** Modified tRNA nucleosides that act as identity elements for aminoacylation

Short name	Full name	Nucleoside position	tRNA	Role in human diseases^d^
I	Inosine	34	Ile	Yes^e^
k^2^C or L	2-Lysidine or 2-lysyl cytidine	34	Ile	–
mnm^5^s^2^U	5-methylaminomethyl-2-thio uridine	34	Glu	Yes
C+	Agmatidine (an L mimic)	34	Ile (*Archaea*)	–
Q	Queuosine^a^	34	Tyr	Yes
Ψ	Pseudouridine^b^	35, 36	Ile, Tyr	Yes
t^6^A	*N* ^6^-Threonylcarbamoyl adenosine	37	Ile	Yes
m^1^G	1-Methyl guanosine	37	Asp, Cys, Pro	Yes
yW	Wybutosine^c^	37	Phe	Yes
m^5^C	5-Methyl cytosine	38	Asp	Yes

^a^Hypermodified guanosine or 7-{[(4,5-*cis*-dihydroxy-2-cyclopenten-1-yl)amino]methyl}-7-deazaguanosine; ^b^ribose is linked to uracil position C5 instead of uracil position N1 in uridine; ^c^heavily hypermodified guanosine; ^d^for an associated role in diseases due to aberrant mt- or cyto-tRNAs, see (422); ^e^only in cyto-tRNA^Ile^ (422)

###### Leucine identity

Cytosolic tRNAs^Leu^ share with tRNA^Ser^ and tRNA^Tyr^ species a long variable arm typical of class II tRNAs. Given this unusual structural feature, the role of the variable arm in the identity was tested very early on. Nucleotide swap experiments resulted in identity changes from serine to leucine (51) and vice versa from leucine to serine (52). Leucine identity was further investigated *in vitro* or *in vivo* in five models covering the three domains of life, notably bacterial *E. coli* (53) and the primitive hyperthermophile *Aquifex aeolicus* (54), archaeal *Haloferax volcanii* (55), and eukaryal *S. cerevisiae* (56,57) and human (58,59) tRNAs.

In *E. coli* tRNA^Leu^, the discriminator base A_73_ but not the anticodon, or the long variable arm, is crucial for leucine identity (60). The absence of an identity element in the anticodon was not surprising since tRNA^Leu^ belongs to the six codon families, and five isoacceptors of tRNA^Leu^ exist in *E. coli* with, however, a common A_35_ nucleotide. Using SELEX on a library of *E. coli* tRNA_1_^Leu^ randomized in the D loop, T loop and variable arm revealed sequence elements crucial for leucylation in the hinge region. The *trans*-Levitt pair A_15_–U_48_, nucleotides G_18_G_19_ and A_20a_ in the D loop were shown to be essential elements. Although the long variable arm is a characteristic feature of the tRNA^Leu^ structure, its sequence and folding were not correlated with the leucylation activity (61). A similar conclusion was obtained by additional studies performed *in vitro* and *in vivo* (62). Other studies showed that *E. coli* tRNA^Leu^ variants missing the anticodon arm and the long variable arm remain active. The aminoacylation of such truncated tRNAs^Leu^ was abolished when the discriminator A_73_ was replaced with C_73_ or when the tertiary interactions between the D and T loops were disrupted, suggesting that these identity elements were still active in the minimal tRNAs (53). More focused mutagenesis of *E. coli* tRNA^Leu^ explicitly demonstrated the importance of tertiary interactions G_18_:U_55_, G_19_–C_56_ and U_54_:A_58_ in leucine identity (63). It can be noted that mutants of *E. coli* and *A. aeolicus* tRNA^Leu^ that remain active in charging also remain active in editing (53,54).

In *S. cerevisiae* tRNA^Leu(UAG)^, insertion of a U residue in the loop region of its long variable arm destroys the stable tetraloop U_47a_UCG_47d_, forming an additional U·G pair in the variable arm that confers serine acceptance to the variant (56). In another study performed on *S. cerevisiae* tRNA^Leu(UAG)^, seven nucleotides were shown to decrease steady-state levels of tRNA leucylation *in vivo* in a triple tRNA^Leu(UAG)^ knockout strain. Strong impact was observed with mutants G_18_, m^1^G_37_, Y_55_ and A_73_, and more moderate decreases with mutants of nucleotides G_2_, G_30_, C_61_ and C_62_. However, A_35_ or G_36_, which are essential for *in vitro* leucine identity, play only a marginal role in the leucylation of tRNA^Leu(UAG)^*in vivo*, but are essential for decoding (57).

In the archaeon *H. volcanii*, leucine identity is defined by the discriminator A_73_ and the long variable arm of tRNA^Leu^, especially the specific loop sequence A_47_CG_47d_ and U_47h_ at the base of the variable arm. Interestingly, the unmodified transcript maintains full cognate leucine acceptance (55).

Together, these results show a mosaic of different situations that can be summarized in a few points. (i) Minimal tRNA variants with the discriminator determinant A_73_ are leucylated in the presence of specific tertiary interactions between the D and T loops in *E. coli* and *H. sapiens* (53,64). (ii) Switches of leucine to serine identity can be achieved in different structural contexts after replacing the discriminator A_73_ with G_73_ (51,64). (iii) Structural variations are tolerated in the long variable arm of *E. coli* tRNA^Leu^ (53). (iv) Sequence-specific recognition of the long variable arm is unique to *Archaea* (55). From the perspective of aaRS recognition, aminoacylation of class II tRNAs predicts the importance of the discriminator base, tertiary nucleotides (at positions 15–48 and 59), the D loop (α and β subdomains), the number of bases and unpaired bases in the variable arm and position 37 in the anticodon loop (55), features that are currently conserved in known tRNA sequences (65).

###### Methionine identity

Early studies performed on variants of tRNA_f_^Met^ enzymatically synthesized *in vitro* showed that recognition of tRNA_f_^Met^ requires highly specific interactions of MetRS with functional groups on the nucleotide bases of the anticodon sequence (66). Mutant tRNA transcripts were later prepared that contained normal and interchanged anticodon sequences. It was confirmed that a tRNA^Val^ variant with a CAU methionine anticodon is charged by *E. coli* MetRS with the same catalytic efficiency as native tRNA^Met^. This indicates that the anticodon contains sufficient information to distinguish methionine and valine tRNAs with high fidelity (67). The methionine recognition system is special since it governs the aminoacylation of elongator and initiator tRNAs which play different roles in protein synthesis and interact with different partners. Although the general principles of recognition of the elongator tRNA by MetRS and the translation machinery are similar in the three domains of life, those of the initiator tRNA show important phylogenetic differences. Formylation of the charged methionyl moiety exists in *Bacteria*, but not in *Eukarya* or in *Archaea* because of the lack of the tRNA_f_^Met^-formyltransferase recognition elements (the 1–72 mismatched pair and the R_11_:Y_24_ base pair disappeared) (68,69). However, the methionine anticodon CAU is important for *in vivo* methionylation of *H. volcanii* tRNA^Met^. Lastly, *E. coli* MetRS utilizes the anticodon nucleotides as determinants to mismethionylate *E. coli* tRNA^Arg(CCU)^ and tRNA^Thr(CGU)^, defining the concept of mischarging identity for MetRS (70).

###### Valine identity

Valine identity is closely related to the isoleucine and methionine identities, due to the common discriminator A_73_ and anticodon A_35_ determinants. This explains the efficient mischarging of tRNA^Ile^ and tRNA^Met^ by ValRS (71). G_20_, in the variable pocket, and G_45_, in the central core of tRNA, are minor recognition elements. Mutations at either the G_3_–C_70_ or U_4_–A_69_ base pairs in the acceptor stem also affect the activity (72), and introduction of a G·U pair at the third or fourth position of the acceptor stem of *E. coli* tRNA^Val^ significantly impairs its activity (73). Transplantation of A_35_, C_36_, A_73_, G_20_ and G_45_ in *E. coli* tRNA^Phe^ containing a regular A-RNA acceptor helix and an tRNA^Val^ anticodon stem is necessary to confer effective valine acceptance (74). The importance of the anticodon is also demonstrated in *Archaea* (68). Atomic group mutagenesis suggests that the unprotonated N1 position in A_76_ of *E. coli* tRNA^Val^, acting as a H-bond donor, is an essential valylation determinant (75).

##### Identity sets recognized by subclass Ib aminoacyl-tRNA synthetases

###### Glutamate identity

Glutamate identity is one of the few examples where a modified base is a major identity determinant, in particular the hypermodified thiol part of mnm^5^s^2^U_34_ at the wobble position of the anticodon (76–78). This role is reinforced by A_37_ next to the anticodon (76). In *E. coli*, the N_73_ discriminator position is not required, but the base pairs G_1_–C_72_ and U_2_–A_71_ in the acceptor stem are weak identity determinants. In summary, the strong identity determinants are U_34_, U_35_, C_36_ and A_37_ in the anticodon loop. In addition, the base pair U_11_–A_24_, the base-triple U_13_–G_22_:A_46_ and the absence of residue 47 serve as major identity determinants in tRNA^Glu^, presumably for the formation of structural features that are recognized by GluRS (79).

###### Glutamine identity

In early work, an *E. coli* tRNA^fMet^ derivative (an amber suppressor tRNA^fMet^) containing an anticodon sequence altered with U_34_ showed a large increase in glutamine acceptance and a large decrease in methionine acceptance (80). The result was consistent with the previously reported aminoacylation with glutamine of the amber suppressor of tRNA^Trp^ (81). Therefore, it was concluded that the central position of the anticodon is involved in tRNA substrate recognition by GlnRS. Analysis of the amber tRNA^Ser^ showed that the substitution of two base pairs in the acceptor helix changed the aminoacylation specificity from serine to glutamine. The importance of base pairs 1–72 and 3–70 for the identity of glutamine was thus established (82). Now, it is accepted that the elements of *E. coli* glutamine identity are located in the anticodon and acceptor stem regions, including the discriminating base (83). Comprehensive kinetics revealed that interactions with the acceptor stem act as strong determinants of tRNA specificity to correctly position the 3′-CCA end in the active site. The 10–25 base pair and central U_35_ are also important binding sites for GlnRS, with G_36_ contributing to both binding and recognition (84). Mutations of these determinants primarily affect *k*_cat_, resulting in up to a 10^5^-fold loss of catalytic activity, far greater than in most other systems (83,85).

Many microorganisms such as the pathogenic bacterium *Helicobacter pylori* do not have a GlnRS but have two divergent glutamyl-tRNA synthetases: GluRS1 and GluRS2. While GluRS1 aminoacylates tRNA^Glu^ as a canonical GluRS, GluRS2 has lost the ability to charge cognate tRNAs^Glu^ and mischarges tRNA^Gln^ to form Glu-tRNA^Gln^ (86,87). GluRS2 rejects tRNAs^Glu^ by predominantly looking at antideterminants located in the acceptor stem, at the first base pair position. The base pair U_1_–A_72_ is found in tRNA^Gln^, while tRNA_1_^Glu^ and tRNA_2_^Glu^ both contain a G_1_–C_72_ base pair. The importance of this position is conserved throughout indirect aminoacylation (87). Amidotransferases that convert Glu-tRNA^Gln^ into Gln-tRNA^Gln^ also rely on the U_1_–A_72_ base pair for recognition of Glu-tRNA^Gln^. In another study, identity elements were revealed in tRNA^Gln^ from *Bacillus subtilis* and *E. coli* for mischarging by *B. subtilis* GluRS, the main recognition element for GluRS being a modified U at the 34th position (88).

##### Identity sets recognized by subclass Ic aminoacyl-tRNA synthetases

###### Tryptophan identity

Data have been collected in five different taxa: *Aeropyrum pernix*, *Arabidopsis thaliana*, *B. subtilis*, *E. coli* and *H. sapiens*, covering the three domains of life. Early studies revealed that the discriminating base, the nucleotides of the anticodon and the first base pair of the acceptor stem are the major identity elements (89–93). More recent studies showed that in *A. thaliana*, the discriminator A_73_ and the C_34_C_35_A_36_ anticodon are also strong determinants. Mutation of the tRNA^Trp^ CCA anticodon to an amber CUA anticodon allows tRNA^Trp(CUA)^ to be mischarged *in vitro* and *in vivo* by plant LysRS (94). In *H. sapiens*, the major determinant is A_73,_ in contrast to *E. coli* and *B. subtilis* where it is G_73_ (95). Three base pairs in the acceptor stem of *B. subtilis* tRNA^Trp^ (G_2_–C_71_, G_3_–C_70_ and G_4_–C_69_), selected from a random library, are required for efficient aminoacylation by cognate TrpRS (96). These pairs, found in the native sequence of *B. subtilis* tRNA^Trp^, are strong identity determinants with a strength between that of the major G_73_ and the minor A_1_–U_70_ and G_5_–C_68_ elements (96). In *A. pernix* hyperthermophile aerobic archaeal tRNA^Trp^, the main identity elements are the discriminator A_73_, the anticodon bases C_34_ and C_35_, and the base pair G_1_–C_72_. The G_2_–C_71_ pair only plays a minor role and acts by a *K*_M_ effect (97). Together, these results suggest that species differences in tryptophan identity are modulated by minor determinants.

###### Tyrosine identity

tRNA^Tyr^ is a type II tRNA with a long extra arm. While the two other type II tRNAs (tRNA^Leu^ and tRNA^Ser^) carry a long variable arm in all organisms and organelles except in animal mitochondria, the long variable arms of tRNAs^Tyr^ have been lost twice: early after the separation of *Bacteria* from *Archaea* and *Eukarya*, and later in parallel with comparable changes in tRNAs^Leu^ and tRNAs^Ser^ in animal mitochondria (98). Studies carried out on the tyrosine identities of *E. coli* and *S. cerevisiae* have shown the critical importance of the A_73_ discriminator and the U_35_ anticodon for tyrosylation [reviewed in (4,99)]. These identity elements, also found in tRNA^Ala^, are sufficient for the easy transformation of a tRNA^Tyr^ into a tRNA accepting both alanine and tyrosine by simple substitution of the U_3_–A_70_ pair with the G_3_·U_70_ alanine identity pair (100). Mutated tRNA with ‘double identity’ was quantitatively aminoacylated with either of the two amino acids *in vitro* (100). As a class II tRNA, the identity of tRNA^Tyr^ is close to that of tRNA^Leu^ and tRNA^Ser^, as demonstrated by identity nucleotide transplantation experiments of several tertiary elements between these tRNAs (55,60). Tyrosine identity is also close to phenylalanine identity since yeast tRNA^Tyr^ with a phenylalanine GAA anticodon is mischarged by yeast PheRS, showing the major role of the anticodon in identity (101). Currently, determinants for tyrosylation are known for cytosolic tRNAs from five taxa (*A. pernix, E. coli*, *Methanocaldococcus jannaschii*, *Pneumocystis carinii* and *S. cerevisiae*) representing the three domains of life. In most of these systems, a small number of nucleotides in tRNAs^Tyr^ govern tyrosine identity in conjunction with adaptation of TyrRSs. Interestingly, the tyrosine system shows that identity elements are not always conserved in evolution. While the A_73_ discriminator is conserved as an identity element in all three domains of life, the first pair 1–72 is not conserved, although it has retained an identity function (102–106). In *Archaea* and *Eukarya*, the identity element is a C_1_–G_72_ pair, whereas in *Bacteria* it is a G_1_–C_72_ pair. This is accompanied by structural changes in TyrRSs to ensure recognition of the first base pair in the tRNA acceptor stem, notably in archaeal TyrRSs of *A. pernix*, *Archaeoglobus fulgidus* and *Pyrococcus horikoshii* (107), and in eukaryal TyrRS of *S. cerevisiae* (108).

##### Identity sets recognized by subclass IIa aminoacyl-tRNA synthetases

###### Alanine identity

The major identity determinant of tRNA^Ala^ was discovered in 1988 when it was shown that the G_3_·U_70_ wobble pair governs the specific charging of *E. coli* tRNA^Ala^ by alanine (11,12). This pioneering work was followed by numerous studies in several laboratories (109–122). The determinant role of G_3_·U_70_ is conserved in evolution, as shown by more recent studies. Conversion of G_3_·U_70_ to A_3_–U_70_ or G_3_–C_70_ eliminates alanylation by insect or human AlaRSs (123) and the introduction of a G_3_·U_70_ pair in a tRNA^Tyr^ confers acceptance of alanine (100). In *A. thaliana* tRNA^Ala^, conversion of the G_3_·U_70_ identity pair to G_3_–C_70_ blocks alanylation, while conversion of G_3_–C_70_ to G_3_·U_70_ in tRNA^Phe^ allows this mutated plant tRNA^Phe^ to be an efficient substrate of plant AlaRS (124). A genetic selection performed on an *E. coli* tRNA^Ala^ knockout strain revealed that tRNA^Ala^ mutants having a variety of sequence combinations in the acceptor stem region can support knockout cell growth. Several mutant tRNAs having substantial activity lacked the G·U wobble pair and instead contained mispairings C–C, C–U or G–A at the 3–70 position (125). In line with this result, atomic group mutagenesis was applied to discriminator A_73_ and base pair 2–76 that severely affected AlaRS recognition when mutated in the context of minihelices. The results revealed a subtle interplay between positive and negative effects on transition state stabilization of the alanylation reaction (126,127). It should be noted that although G_3_·U_70_ is the main identity element of tRNA^Ala^, distinct modes of G·U pair recognition have been characterized by comparing bacterial AlaRS with eukaryotic/archaeal AlaRS (128–130). Finally, very rare exceptions occur in mitochondria, where the G_3_·U_70_ pair may be absent or translocated (131–133) (details below in the section dedicated to mitochondrial identities).

###### Glycine identity

Glycylation systems are very complex because neither the oligomeric structure of GlyRS (dimers in *Eukarya* and *Archaea* and dimers or heterotetramers in *Bacteria*) nor the discriminator bases (U_73_ in bacterial tRNA^Gly^ and A_73_ in eukaryal and archaeal tRNAs^Gly^) are conserved in evolution. This diversity impacts glycine identity, since the consensus sequence of tRNAs^Gly^ only contains a G_1_–C_72_ pair in the acceptor arm, C_35_ and C_36_ in the anticodon and the (G_10_–Y_25_):G_45_ triple involved in tRNA folding (134). Early studies showed that mutation of the U_73_ discriminator base of *E. coli* tRNA^Gly^ reduced glycine acceptance, revealing that it acts as an identity element (135). A similar result was observed *in vivo* using the amber suppressor of tRNA^Gly^ (136). The reduction of the suppressor activity *in vivo* was even much more severe than that in the *in vitro* aminoacylation experiment (136). Mutation studies on tRNA^Gly^ of *E. coli*, *T. thermophilus* and the yeast *S. cerevisiae* showed that the identity set also contains the first and second base pairs, G_1_–C_72_ and C_2_–G_71_, in the acceptor stem, and the anticodon nucleotides C_35_ and C_36_. However, differences exist between yeast and the two bacteria in the acceptor stem. The first base pair, G_1_–C_72_, is important for glycylation in *E. coli* and *T. thermophilus*, whereas the second and the third base pairs are important in yeast (137). *Thermus thermophilus* GlyRS also recognizes the C_50_–G_64_ pairs together with the G_10_, U_16_, C_35_ and C_36_ single residues (138). The glycine identity set of archaeal *A. pernix* tRNA^Gly^ includes C_35_ and C_36_ from the anticodon and the C_2_–G_71_ and G_3_–C_70_ base pairs from the acceptor stem, but does not require discriminator base A_73_ (139). Currently, no examples of higher eukaryal tRNA^Gly^ identity elements are known.

###### Histidine identity

This identity is unique due to the presence of an additional residue G_−1_ in all three domains of life (although some organisms lack this residue; see below). The essential role of G_–1_ in identity and possibly of the G_–1_–C_73_ pair, together with that of the anticodon, was first demonstrated for *E. coli* (140) and *S. cerevisiae* tRNA^His^ (141). HisRS efficiently aminoacylates minihelix (13 bp) and microhelix (8 bp) RNAs resembling the tRNA acceptor stem which contain G_–1_–C_73_. Transplantation of this base pair is also sufficient to confer histidine acceptance to a tRNA^Ala^ minihelix (110). These identity conversions mediated by the G_–1_–C_73_ base pair were exploited to isolate secondary site revertants in *E. coli* HisRS which restore histidine identity to a tRNA^His^ suppressor carrying a G_–1_·U_73_ pair. The revertant substitutions were found in the anticodon-binding domain located in the C-terminal domain of HisRS, demonstrating that the anticodon of tRNA^His^ also plays an important role in tRNA selection *in vivo* (142). In the hyperthermophile archaeon *A. pernix*, G_–1_ but also A_–1_ and C_–1_ can be recognized by cognate HisRS together with a weak participation of the discriminator base C_73_, in contrast to the anticodon that is not recognized (143). Atomic group mutagenesis was carried out at the –1 to 73 position of chemically synthesized microhelix^His^ substrates. The results suggested that the G_–1_ base serves to position the 5′-monophosphate, which is critical for aminoacylation. Furthermore, the 6-keto oxygen of G_–1_ and the major groove amine of C_73_ contribute to HisRS recognition. This supports the existence of a canonical G_–1_–C_73_ pair (144).

In most *Bacteria* and *Archaea*, G_–1_ is encoded by the tRNA gene. In contrast, G_–1_ incorporation occurs post-transcriptionally in *Eukarya* and is catalyzed by tRNA^His^-guanylyltransferase (Thg1), an enzyme that catalyzes the addition of nucleotides in the 3′–5′ direction, in contrast to all known DNA and RNA polymerases (145,146). Despite the lack of sequence similarity, Thg1 enzymes share structural homology with canonical 5′–3′ DNA polymerases and recognize the tRNA^His^ anticodon during the maturation process.

The crystal structure of *T. thermophilus* HisRS complexed with tRNA^His^ reveals that G_–1_ recognition is principally based on non-specific interactions with this base and is made possible by an enlarged binding pocket (for the extra base G_–1_) absent in other aaRSs, while the anticodon triplet makes additional specific contacts with the enzyme. The structural complementarity between the 5′ extremity of tRNA and the enzyme is probably a result of co-evolution of both tRNA^His^ and HisRS (147). Divergence of bacterial histidylation rules was observed in some groups of α-*Proteobacteria*. In this clade, neither the genetically encoded G_–1_ nor C_73_, which are essential for histidine identity in *E. coli*, is present. Instead, tRNA^His^ contains A_73_, which in yeast is a less essential but still important element of histidine identity. In parallel, the motif II loop in HisRS that recognizes the discriminator base 73 of tRNA^His^ covaries perfectly with the presence of C_73_ (148). This was experimentally validated in *Caulobacter crescentus*, a G_–1_-lacking α-*Proteobacterium*, whose *in vitro* identity is based on A_73_, U_72_ and the anticodon (149). Similarly, in some protists, tRNA^His^ lacks G_–1_ (150), and these organisms do not possess the tRNA^His^-guanylyltransferase gene. In the case of the protists *Trypanosoma brucei* and *Acanthamoeba castellanii*, a non-canonical G_−1_-independent HisRS charges the atypical tRNAs^His^ (150).

###### Proline identity

Proline identity shows unexpected complexity, with deviations in the identity sets of the three *E. coli* tRNA^Pro^ isoacceptors and variations in the structures of ProRSs that are divided into two groups, prokaryal-like and eukaryal/archaeal-like (151,152). Beside strong determinants at the extremities of tRNA (G_72_, G_35_, A_37_ followed by G_36_ and A_73_) and the Levitt G_15_C_48_ pair, additional determinants were found in the major tRNA^Pro(CGG)^, notably G_37_, G_72_, G_49_ and U_17a_ (153,154). In archaeal *A. pernix* tRNA^Pro^, the G_1_–C_72_ identity pair on top of the acceptor stem completes the discriminator and anticodon determinants (155). Analysis of ProRS sequences in the three domains of life revealed that the sequences are divided into two evolutionarily distant groups (152). While A_73_ is strictly conserved in bacterial and archaeal tRNAs^Pro^, a C_73_ pyrimidine is found in eukaryal tRNAs^Pro^ and the base pair 1–72 is inverted (G_1_–C_72_). Analysis of aminoacylation revealed that, while anticodon recognition has been maintained during evolution, significant changes in acceptor stem recognition have occurred. The C_1_–G_72_ pair is a strong determinant in *E. coli* tRNA^Pro^, but the G_1_–C_72_ pair is without effect in human tRNA^Pro^ where identity relies predominantly on the anticodon branch. Atomic group mutagenesis was carried out to probe the role of sugar–phosphate backbone interactions in recognition of human tRNA^Pro^. A network of interactions with the first base pair and the discriminator base was revealed in both *E. coli* and human tRNA^Pro^. Therefore, unlike the bacterial system, backbone-specific interactions contribute much more to tRNA recognition by the human enzyme than base-specific interactions (156). Finally, in *E. coli* tRNA^Pro^, m^5^ methylation of G_37_, which is known to suppress frameshift errors, also contributes to proline identity since its absence significantly affects prolylation efficiency (157).

###### Serine identity

Identity sets are known in various tRNA^Ser^ isoacceptors of *E. coli* (51,82,158–160), *S. cerevisiae* (56), *H. sapiens* (98), *Archaea* (161) and *Zea mays* (162). Given that serine is assigned by six codons in the genetic code, the tRNA^Ser^ anticodon can hardly play a major role in serine identity. Indeed, serine identity is of astonishing complexity, due to a phylogenetic divergence in size and orientation of the variable region and, more generally, to the variability of the tRNA^Ser^ and SerRS sequences (163). For instance, the G_1_–C_72_ and G_2_–C_71_ pairs are absolutely conserved in *E. coli* and in most bacterial tRNA^Ser^ isoacceptors, while the A_27_–U_43_ pair is conserved in yeast tRNA^Ser^ and in some eukaryal (not human) but not in archaeal and bacterial tRNA^Ser^ isoacceptors (164). Interestingly, the discriminator G_73_ in *E. coli* tRNA^Ser^ acts as an identity determinant *in vivo*, but not in unmodified transcripts *in vitro* (165). The functional importance of sequence differences in the yeast and human tRNA^Ser^ acceptor stems, which account for species-specific serylation, was confirmed *in vivo* in yeast (56). Overall, serine identity relies primarily on the variable arm and is independent of the anticodon (52).

Comparing the different domains of life, several differences are observable. The main determinants for tRNA serylation by human SerRS are the large variable arm and the G_73_ discriminator (58,59). In *E. coli* tRNA^Ser^, the moderate importance of the discriminator G_73_ is strengthened by determinants in the acceptor stem (165). Interestingly, *E. coli* SerRS selectively recognizes tRNA^Ser^ on the basis of its characteristic tertiary structure rather than the nucleotides specific to tRNA^Ser^ (165). Due to the co-existence of two dissimilar SerRSs in the archaea *Methanosarcina barkeri* (one bacterial-like, the other specific to methanogenic *Archaea*), two tRNA recognition modes with distinct overlapping identity sets co-exist in the three tRNA^Ser^ isoacceptors (161). The discriminator base G_73_ followed by the weaker G_30_–C_40_ pair are strong identity elements in bacterial and archaeal SerRSs. Other determinants are required for serylation by methanogenic SerRS, including the G_1_–C_72_ pair and several unpaired nucleotides at the base of the extra stem in the variable region that control stem helicity and tertiary interactions (161). As a result, the serine identity elements are used differently in *Archaea* and *Eukarya* (166). In *Z. mays* plants, the discriminator base G_73_ is by far the strongest determinant of serylation by cytosolic SerRS, as is the case with human cytosolic SerRS (162). As the anticodon of tRNA^Ser^ does not play a role in aminoacylation, variants of tRNA^Ser^ with anticodon changes cause similarly high levels of mistranslation. tRNA^Ser^ mutants with proline anticodons (UGG) remain serylated and therefore cause mistranslation in yeast (167).

###### Threonine identity

Threonine identity is unique, since the discriminator position 73 does not strongly participate in threonine identity, except in *H. volcanii* tRNA^Thr^ where U_73_ is an identity determinant (168) and in yeast where substitution of the discriminator base A_73_ by G_73_ or C_73_ impairs the threonine accepting activity (169). Threonine identity sets are known in five species, notably in tRNA^Thr^ from *E. coli* (170), *T. thermophilus* (171), *H. volcanii* (168), *A. pernix* (172) and *S. cerevisiae* (169). Identity elements consist of base pairs in the acceptor stem and nucleotides of threonine anticodons. Identity elements in the acceptor stem are not fully conserved between species and the identity sets alone are not sufficient to confer tRNA^Thr^ charging fidelity, as shown by the strong mischarging capacities of the tRNA^Thr^ isoacceptors (173). In *Vertebra*, the G_4_·U_69_-containing tRNA^Thr^ incorporates alanine, but mistranslation is prevented by a robust *trans*-editing activity of ThrRS towards alanyl-tRNA^Thr^ (173). Interestingly, in yeast, the G_3_·U_70_ wobble pair in tRNA^Ala^ acts as an antideterminant for ThrRS (169).

##### Identity sets recognized by subclass IIb aminoacyl-tRNA synthetases

###### Aspartate identity

Aspartate identity has been studied in *S. cerevisiae*, *E. coli* and *T. thermophilus* tRNAs (4,174). It is mainly based on five strong determinants (G_73_, G_34_, U_35_, C_36_ and C_38_) which are conserved during evolution. However, there are differences in the strength of the determinants in eukaryal and bacterial systems, with the anticodon determinants being strongest in *Bacteria* (175). The two pairs G_1_–C_72_ and G_2_–C_71_, strictly conserved in the bacterial tRNA^Asp^, are crucial for identity and act differentially. The G_2_–C_72_ pair is a minor determinant (175) while the C_1_–C_72_ pair helps to position the 3′ acceptor end in the catalytic site of *E. coli* AspRS (176). The charging specificity of yeast tRNA^Asp^ is achieved by the modified base m^1^G_37_ which acts as an antideterminant against arginyl-tRNA^Asp^ formation by yeast ArgRS (177). The iodine cleavage of yeast tRNA^Asp^ transcripts substituted with phosphorothioates revealed the critical role of specific phosphates during AspRS recognition. The cognate AspRS protects the phosphate groups of four determinants (G_34_, U_35_, U_25_ and G_73_), and mutation of these nucleotides results in the loss of phosphate protection in the mutated regions while the overall protection pattern remains unchanged (178). In another study, active variants of yeast tRNA^Asp^ lacking the D and T arms were constructed, leading to minimal active tRNAs^Asp^ that mimic mt-tRNAs (179). In these minimal structures, the rules of identity are preserved, and aminoacylation activity remains strictly dependent on the discriminator G_73_ and the three anticodon nucleotides (179).

In yeast AspRS, the improved aminoacylation efficiency results from the acquisition of a lysine-rich N-terminal extension that interacts with the anticodon stem of tRNA^Asp^ with, however, a loss of specificity and a risk of mischarging, especially of tRNA^Glu(UUC)^ and tRNA^Asn(GUU)^, which have identical G_73_ discriminator and anticodons close to the aspartate anticodon GUC (180,181). More generally, N-terminal extensions are conserved in eukaryal class IIb aaRSs where they can control cellular precision of tRNA charging. In yeast, the aspartate aminoacylation system appears to be connected to the arginine system, with the early observation that ArgRS aminoacylates tRNA^Asp^ and more recently with the observation that a minor tRNA^Arg^ is a cryptic tRNA^Asp^ (25,182). In yeast, the aspartate aminoacylation system appears to be connected to the arginine system as suggested by the early observation that ArgRS aminoacylates tRNA^Asp^ (182). This hypothesis has been reinforced by the observation that a minor tRNA^Arg^ is a cryptic tRNA^Asp^ (25). Finally, functional aspartylated tRNA^Asp^ mutants were selected *in vitro* in a selection procedure applied to a yeast tRNA^Asp^ library randomized at the anticodon triplet level. The active tRNAs mostly carried the original aspartate anticodon GUC, but one mutated tRNA had an alanine anticodon GGC (183). This mutated tRNA exhibits a 19-fold drop in catalytic efficiency resulting from a 4-fold reduction in affinity and a 5-fold drop in *k*_cat_ (184). These substantial alterations in catalytic parameters certainly protect cells from effective *in vivo* aminoacylation of tRNA^Ala^ which, moreover, has an A_73_ discriminator not favorable to aspartylation.

###### Asparagine identity

A first study demonstrated that bacterial asparagine identity is transferable into a tRNA^Lys^ by transplantation of the three base anticodon and the G_73_ discriminator of tRNA^Asn^ (135). The aminoacylation levels in *E. coli* of tRNA^Asn^ mutants confirmed that both the anticodon and the discriminator base are important for aminoacylation of tRNA^Asn^ (185). Although early attempts to convert the *E. coli* tRNA^Asn^ into an amber suppressor by modification of its anticodon sequence failed (186), several active glutamine-inserting suppressors were obtained using *in vivo* selection. The mutated suppressors all had substitutions in the first base pair 1–72 that reduced their stability and were all glutamine insertion suppressors (187). In the yeast *S. cerevisiae*, the identity conferred by the bases of the anticodon and discriminator is enhanced by the post-transcriptional modification t^6^A_37_ that prevents aspartylation (188). Despite similarity between *Plasmodium falciparum* and *H. sapiens* tRNA^Asn^ sequences, cross-species aminoacylation is not observed with the corresponding enzymes. The human enzyme does not recognize the plasmodial transcript of tRNA^Asn^, and the *P. falciparum* enzyme charges the human transcript of tRNA^Asn^ with an 8-fold reduction compared with its cognate tRNA^Asn^. Subtle differences in the two tRNA^Asn^ sequences or the use of *in vitro* transcripts deprived of post-transcriptional modifications could prevent cross-recognition between species (189).

###### Lysine identity

The role of U_35_ in the lysine identity of tRNA^Lys^ was initially suggested by the finding that several amber suppressor tRNAs with a U_35_ inserted lysine into the suppressed protein (19,190). Discriminator base A_73_ also played an important role in tRNA^Lys^ identity since substitution to G_73_ reduced the suppression efficiency and the resulting tRNA became a partially glutamine-inserting suppressor (19). The unmodified *E. coli* tRNA^Lys^ transcript showed a 140-fold lower lysine charging activity than the native tRNA^Lys^, suggesting the involvement of base modifications in recognition. Substitution of the discriminator base A_73_ by any of the other bases confirmed the decrease in lysine acceptor activity observed *in vivo*. Substitutions of anticodon nucleotides showed the involvement of all three bases in the lysine identity (20). Therefore, as with the aspartate and asparagine identities, the lysine identity relies on the anticodon triplet and the A_73_ discriminator, which differs from the G_73_ of the first two systems. In *H. sapiens*, LysRS aminoacylates an RNA minihelix that mimics the amino acid acceptor stem–loop domain of tRNA^Lys^, but without specificity of sequence. However, the continuity between the acceptor and anticodon domains is important for efficient lysylation (191,192). The UUU anticodon is sufficient for the acceptance of lysine of human tRNA^Lys^ since its transplantation into tRNA^Asp^ or the initiator tRNA^Met^ confers lysine identity to these tRNAs. As in the aspartate system, the specificity of lysylation is facilitated by contacts with the N-terminal helical extension of human LysRS (192).

##### Identity sets recognized by subclass IIc aminoacyl-tRNA synthetase

###### Phenylalanine identity

Phenylalanine identity was investigated in tRNA^Phe^ from five organisms (*A. pernix*, *E. coli*, *T. thermophilus*, *S. cerevisiae* and *H. sapiens*) covering the three domains of life. The common identity set of phenylalanine, revealed by different strategies, contains four strictly conserved elements which are the anticodon nucleotides G_34_, A_35_ and A_36,_ and the discriminator base A_73_. Additional elements are found, such as G_20_ in *S. cerevisiae* and *A. pernix* or U_20_ and U_59_ in *E. coli* (193–195)*. In vitro* selection from a random library was used in *E. coli* to isolate active tRNA^Phe^ variants. Critical elements for phenylalanylation of tRNA were thus identified at the three positions of the anticodon G_34_, A_35_ and A_36_, and at the nucleotides of the variable pocket (U_20_ and U_59_) (196,197). The two lower base pairs (G_30_–C_40_ and A_31_–U_39_) in the anticodon stem of tRNA^Phe^ are also recognition elements for human PheRS (198). The strength of determinants is significantly greater in human than in yeast tRNA^Phe^. Interestingly, tRNA^Phe^ from bacteriophage T5 shows a non-identical mode of recognition by *E. coli* PheRS at low and high concentrations of Mg^2+^, suggesting that the local conformation of the tRNA is essential for recognition by bacterial PheRS (199). Finally, an amber suppressor derived from native plant tRNA^Phe^ showed little suppressor activity *in vivo* in *A. thaliana* and was poorly phenylalanylated *in vitro*, suggesting that the anticodon is also a major identity determinant for tRNA^Phe^ in plant cells (124).

#### Post-transcriptional modifications as identity signals for aminoacylation

In general, tRNA modifications do not participate in tRNA identity, as clearly shown for leucine identity in *H. volcanii* (55) and proline and tyrosine identities in *S. cerevisiae* (103,154). The main role of the modifications is in maintaining the structure of the tRNA and the decoding of the codons on the ribosome (200). However, in some cases, modifications are crucial and play an active role by acting as aminoacylation determinants (Table 2) or antideterminants (Table 3) (78). Decreases in the activity of *in vitro* transcribed tRNAs lacking modified bases have been observed for several identities in *E. coli* (cysteine, glutamate, isoleucine, lysine and phenylalanine) and *S. cerevisiae* (isoleucine and phenylalanine) (78). However, it is difficult to attribute the inhibitory effect to one or a combination of modifications. Finally, the low number of modified nucleotides essential for aminoacylation does not mean that only a few aaRSs use post-transcriptional modifications of tRNAs as identity signals, but rather reflects the fact that they have rarely been studied.

**Table 3.** Antideterminants identified in tRNAs that prevent erroneous recognition of aaRS

Antideterminant	tRNA (organism)	Against aaRS
A_1_–U_72_^a^	tRNA^Trp^(*E. coli*)	MetRS
C_2_–G_71_	tRNA^Leu^ (*E. coli*)	SerRS
G_2_·U_71_	tRNA^Lys^^b^ (*B. burgdorferi*)^c^	LysRS-1
G_3·_U_70_	tRNA^Al^^a^ (*S. cerevisiae*)	ThrRS
	cyto-tRNA^Al^^a^ (*D. melanogaster*)	mt-AlaRS
U_30_·G_40_	tRNA^Ile^ (*S. cerevisiae*)	LysRS
k^2^C_34_	tRNA^Ile^ (*E. coli*)	MetRS
C + C_34_	tRNA^Ile^ (*Archaea*)	MetRS
A_36_	tRNA^Arg^ (*E. coli*)	TrpRS
t^6^A_37_	tRNA^Asn^ (*S. cerevisiae*)	AspRS
G_37_	tRNA^Ser^ (*S. cerevisiae*)	LeuRS
m^1^G_37_	tRNA^Asp^ (*S. cerevisiae*)	ArgRS
A_73_	tRNA^Leu^ (*H. sapiens*)	SerRS
G_73_	tRNA^Ser^ (*E. coli*)	LeuRS and TyrRS
	tRNA^Ser^ (*H. volcanii*)	LeuRS

^a^A_1_–U_72_ is a context-dependent negative identity elemente; ^b^except in *Nanoarchaeota*, found in all archaeal phyla; ^c^probably applies to Spirochetes (a bacterial phylum).

The base modifications that affect the identity of tRNA aminoacylation are mainly located in the anticodon loop (Table 2). At the anticodon wobble position, the thio group of s^4^U_34_ in *E. coli* tRNA^Glu^ and the inosine I_34_ of *S. cerevisiae* tRNA^Ile^ are strong identity elements (47,77). In addition, Ψ_36_ is a weak aminoacylation determinant in tRNA^Ile^ where its main role is to prevent misreading of Ile codons (47). The yW_37_ residue has a dual identity role, weak for aminoacylation by PheRS and strong for decoding tRNA^Phe^ on the ribosome (78). The weak tyrosylation activity of *E. coli* tRNA^Tyr^ when Q_34_ is replaced by C_34_ also suggests its identity role (78). At position 37 adjacent to the anticodon, t^6^A_37_ in *E. coli* tRNA^Ile^ and mammalian cyto-tRNA^Ile^ as well as m^1^G_37_ in *M. mazei* tRNA^Cys^ are determinants for aminoacylation (41,49,201). Interestingly, t^6^A_37_ that is crucial for isoleucylation in most prokaryotic IleRSs is not an identity element in *Bacteria* and its role in translation might vary greatly between organisms (48). t^6^A_37_ functions as a determinant of human cytoplasmic IleRS (49). In mouse tRNA^Asp^, m^5^C_38_ is essential for aminoacylation *in vivo* (202). Often, the modifications and hypermodifications involved in identity and located in anticodons have a dual function in both aminoacylation and codon reading (200,203). Some RNA modifications present in both tRNAs and tRNA-like structures (TLSs) of mRNAs suggest links between tRNA biology and mRNA regulation (204).

#### Distribution of identity elements on the cloverleaf structure of tRNAs

**Figure 3.**
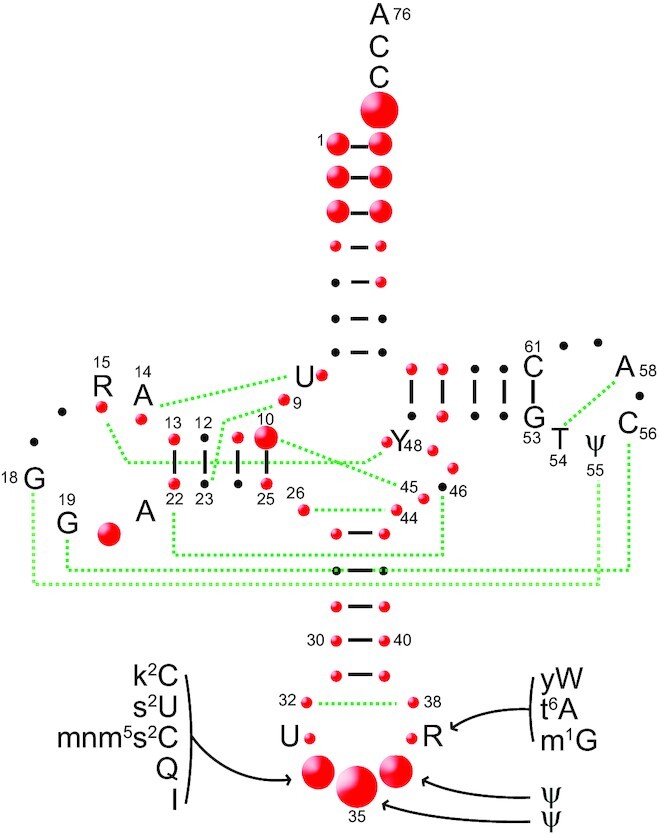
Positions occupied by identity elements in canonical tRNA cloverleaf folding, including identity-determining post-transcriptional modifications. Conserved and semi-conserved nucleotides, as well as tertiary interactions (broken green lines) are shown. The size of red bullets schematizes the extent of occupation in the canonical tRNA sequence (large for the four heavily occupied positions, medium for the eight significantly occupied positions and small for the 32 poorly occupied positions). The standard numbering of positions is used as in Figure 1. Variable region (nucleotides 44–48) with up to 16 nts for extra arms (47a to 47p). Modifications characterized as identity determinants for aminoacylation in anticodon loops are shown next to identity positions; they are displayed in standard abbreviations.

Identity elements are well known in bacterial and eukaryal tRNAs, in contrast to the poorly known determinants in archaeal tRNAs. Except for the *E. coli* and *S. cerevisiae* systems, which have 20 and 18 identity sets known to date, data from other organisms are still sparse. Several identity sets were established in *T. thermophilus* and *H. sapiens* tRNAs and in tRNAs from a dozen other organisms, notably four archaeal phyla. Identity elements were sometimes validated by chemical probing and crystallography. In general, a given identity is specified by a limited number of determinants (2–11 nt) (Table 1). All 20 tRNA families show determinants in both acceptor stems and anticodon loops, except the tRNA^Ala^ and tRNA^Ser^ families. Discriminator and anticodon positions are by far the most represented (Figure 3). The highest diversity in determinants specifying aminoacylation occurs for tRNAs recognized by class IIa aaRSs. The eukaryal identities of glutamine, glutamate, asparagine and lysine are poorly documented (Table 1).

Only 11 identities utilize the complete anticodon triplet as a determinant (Cys, Ile, Met, Gln and Trp anticodons recognized by class I aaRSs, and His, Thr, Asp, Asn, Lys and Phe anticodons recognized by class II aaRSs). Identity sets can overlap (i.e. a given nucleotide at the same tRNA position can code for several identities in a given organism), such as A_73_ coding for 14 identities or C_35_ coding for 15 identities. The distribution of determinants varies when comparing aminoacylated tRNAs by class I or class II aaRSs and, more precisely, when comparing the same identity in *Bacteria*, *Eukarya* and *Archaea*. With the exception of the glutamine, glutamate, asparagine and lysine identities, the other 16 identities have conserved determinants in anticodons and the extremity of acceptor stems in model organisms from all three domains of life. Few determinants are found in the D loop due to sequence and structural constraints imposed by the formation of tertiary interactions with the T loop and the presence of determinants for the editing reaction catalyzed by class 1 aaRSs (63,205).

Many aminoacylation systems use determinants located in the central ‘core’ region of tRNA that forms tertiary atypical interactions, such as the non-Watson–Crick 15:48 interaction that connects the D loop to the variable arm and participates in the cysteine and proline identities in bacteria (34,154). However, data are still lacking for tRNAs specifying valine, tyrosine, threonine, asparagine and lysine identities. Likewise, the conserved U_8_:A_14_ tertiary pair in tRNA^Leu^ is crucial for recognition by the primitive bacterial *A. aeolicus* LeuRS (206). Altogether, this indicates the importance of the tRNA shape in aminoacylation identities.

### Negative determinants or antideterminants

In addition to positive determinants, negative elements (antideterminants) contribute to identity by blocking false recognitions between aaRSs and non-cognate tRNAs, thus providing an additional layer of specificity control. Antideterminants in tRNA can be isolated nucleotides (59,207), modified residues (38,46,177,208) or base pairs (165,209–211). Table 3 shows a panel of antideterminants against identified aaRSs, located in the acceptor and anticodon arm of tRNA. The first modified nucleotides acting as antideterminants were found in the anticodon loop, such as k^2^C_34_ (i.e. lysidine or L_34_) in minor *E. coli* tRNA_2_^Ile^ which blocks recognition by *E. coli* MetRS (38), and m^1^G_37_ in yeast tRNA^Asp^ against yeast ArgRS (177,208).

In the acceptor stem, the A_1_–U_72_ base pair is a context-dependent negative identity element of *E. coli* tRNA^Trp^ (211). Likewise, the C_2_–G_71_ pair in *E. coli* tRNA^Leu^ is a negative identity element against *E. coli* SerRS (165) whereas in the archaeal *H. volcanii* tRNA^Ser^ discriminator G_73_ acts as an antideterminant against LeuRS (55). In *E. coli* tRNA^Ser^, G_73_ is also an antideterminant for LeuRS and TyrRS (161,165). An amber suppressor corresponding to the *S. cerevisiae* tRNA^Ile^ carries a U_30_·G_40_ wobble pair in the anticodon stem that is a negative signal for the *E. coli* LysRS interaction under heterologous expression conditions (209).

In insects, including *D. melanogaster*, the base pairs 2–71 and 3–70 found in cytosolic tRNA^Ala^ behave as antideterminants for mitochondrial AlaRSs that cannot charge these tRNAs^Ala^ because of a shifted mode of recognition (131,132). Interestingly, the G_3_·U_70_ pair in yeast tRNA^Ala^ that blocks interaction with yeast ThrRS was predicted by an algorithm (212), confirming previous functional data (169). Many other putative antideterminants in yeast tRNAs have been predicted and await experimental validation (212).

Other examples are determinants in *E. coli* tRNA^Gln^ that are antideterminants in *E. coli* tRNA^Glu^ and, conversely, determinants in tRNA^Glu^ that are antideterminants in tRNA^Gln^ (213). In *H. pylori*, GluRS2 mischarges tRNA^Gln^ to form Glu-tRNA^Gln^ and rejects tRNA^Glu^ by looking at the antideterminant base pair G_1_–C_72_ that is found in tRNAs^Glu^ (87). In *S. cerevisiae*, tRNA^Leu^ becomes an efficient serine acceptor when unmodified (56). Several antideterminants can co-exist in a given tRNA, such as the base pair U_28_–A_42_ in the anticodon stem and the discriminator base A_37_ in unmodified yeast tRNA^Trp^ that act as negative elements for bovine TrpRS (214).

On the other hand, amino acids in aaRSs can play an equivalent antidetermining role against false aminoacylations of non-cognate tRNAs. For instance, several substitutions in *E. coli* MetRS induce recognition of nonsense suppressors, without affecting recognition of native tRNA^Met^ (215). In *Bacillus stearothermophilus* TyrRS, Glu152 acts as an antideterminant for non-cognate tRNAs by electrostatic and steric repulsions (216). Some amino acids in aaRSs can also have dual positive and negative functions, as found in yeast AspRS (217,218) and *E. coli* GlnRS (219).

## IDENTITIES IN ATYPICAL tRNA FOLDS AND ORGANELLAR tRNAS

### Structural diversity in the tRNA world

#### Non-canonical cloverleaves and atypical folds in cytosolic tRNA and tRNA-like structures

A variety of non-canonical tRNA cloverleaves (220) and viral TLSs (221,222) aminoacylable by standard aaRSs have been described. Other tRNA mimics recognized by aaRSs are present in mRNAs (223). RNA fragments of the large tRF (tRNA-derived RNA fragment) family are additional tRNA mimics (224). They arise from individual transcription units or result from the processing of canonical tRNAs, and some interact with aaRSs (225).

**Figure 4.**
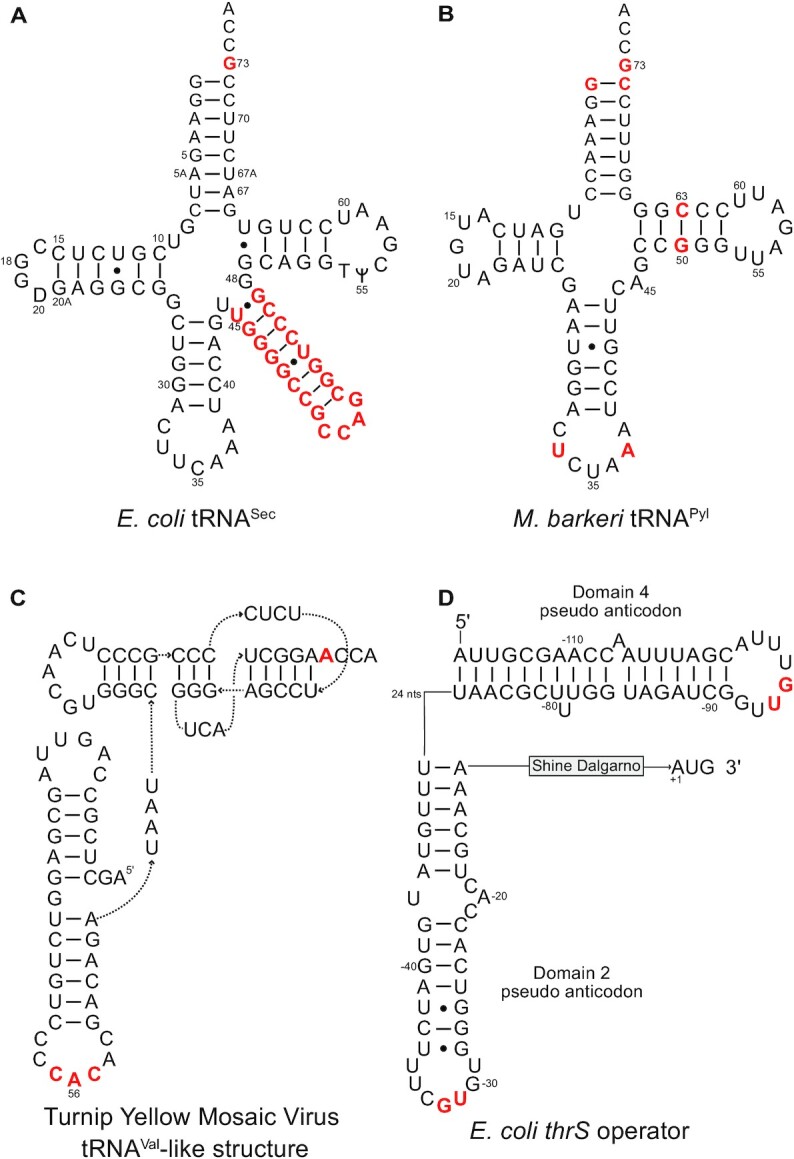
A panel of atypical RNA folds present in tRNAs aminoacylated by aaRSs or only recognized by aaRSs. (**A**) *E. coli* tRNA^Sec^. (**B**) *M. barkeri* tRNA^Pyl^. (**C**) Turnip yellow mosaic virus (TYMV) tRNA-like structure (TLS) with valine-charging capacity. (**D**) *E. coli thrS* operator. Experimentally characterized identity elements are in red font. For easier comparison, the numbering of atypical tRNA folds is as in canonical tRNAs with, for example, positions 73 and 34–36 for discriminator bases and anticodons. For TYMV and mRNA TLSs, sequence numbering is from 3′ to 5′ ends of the molecules with starts at A_1_ (in the -CCA_OH_ accepting end of the viral TLS and the A_1_UG triplet next to the Shine and Dalgarno sequence in the mRNA TLS).

Figure 4 shows four atypical tRNA folds that highlight deviations in helical regions and the presence of pseudoknots, but also shows conserved features, including anticodon loops. Selenocysteine-specific tRNAs, ubiquitously present in life, possess atypical secondary structures as in *E. coli* tRNA^Sec^ (Figure 4A) (226). In *M. barkeri*, the D loop is small and the anticodon arm of tRNA^Pyl^ is unusually long (Figure 4B) (227). The presence of a pseudoknot at the 3′ terminus of many viral RNA genomes was discovered in the TLS^Val^ of turnip yellow mosaic virus (TYMV) (Figure 4C) (222,228). Note the presence of standard anticodon loops in some viral TLSs with anticodon triplets that match the acceptor identity, such as the valine CAC anticodon in TYMV TLS^Val^ (229). Other TLSs lack the anticodon, such as the intricate TLS^Tyr^ from brome mosaic virus (BMV) that is lacking a tyrosine anticodon in its short pseudo-anticodon loop of four nucleotides (230). tRNA mimicry is also responsible for the translational control of the ThrRS gene of *E. coli*. Its 5′-untranslated region contains a *thrS* operator which mimics a tRNA L-shape, with domain 2 corresponding to the anticodon loop and stem of tRNA^Thr^, but lacking an equivalent to the acceptor arm replaced by a domain mimicking a second anticodon stem (Figure 4D) (231,232).

#### Structural diversity in organellar tRNAs

**Figure 5.**
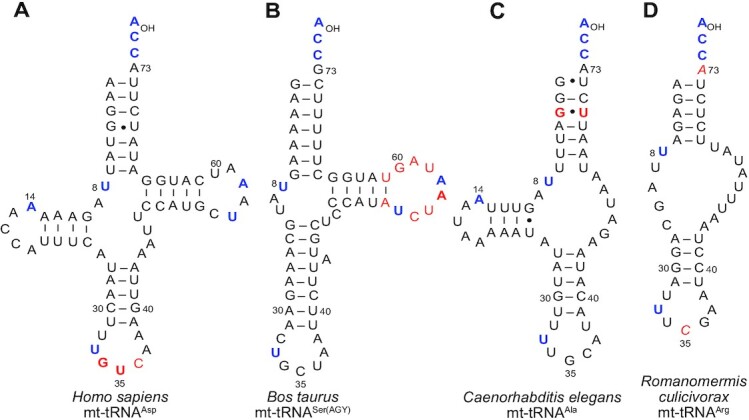
Typical 2D folds in the four mt-RNA structural families indicating partial conservation of universal features necessary for canonical 3D folding, as found in representative mammalian and/or nematode mt-RNAs. (**A**) Canonical-like cloverleaf folding, (**B**) folding missing D arm, (**C**) folding missing T arm, and (**D**) folding missing both D and T arms. The numbering is based on that of canonical tRNAs. Conserved and semi-conserved nucleotides are in blue bold scripts. Experimentally characterized identity determinants are in red font (in bold for major determinants). Predicted determinants in *R. culicivorax* tRNA^Arg^, based on *E. coli* tRNA^Arg^, are in red italic font.

In the world of tRNAs, organellar tRNAs show the greatest structural diversity as evidenced by mt-tRNAs (Figure 5). They include examples with altered cloverleaves such as human mt-tRNA^Asp^ (Figure 5A) or missing either the D arm [e.g. *Bos taurus* mt-tRNA^Ser(AGY)^] (Figure 5B) or the T arm (e.g. *Caenorhabditis elegans* mt-tRNA^Ala^) (Figure 5C) (233). Remarkably, armless structures deprived of both D and T arms are predicted in the nematode *Enoplea* (233). These bizarre armless structures, as first discovered in *C. elegans* (234), are common in nematodes, mites, arachnids and insects (235,236). The length of the sequences varies from 70 nt, for example for human mt-tRNA^Asp^, to 44 nt for *Romanomermis culicivorax* mt-tRNA^Arg^ (Figure 5D), the shortest experimentally characterized natural tRNA to date (233). This length is even predicted to be shorter for several spider mt-tRNAs (235,237) and for tRNA^Gln(UUG)^ from a common plant pest, the acariform mite *Tetranychus urticae* (238).

Some of the conserved and semi-conserved nucleotides characterizing standard tRNAs are present in mt-tRNAs and therefore support their L-shaped conformation. Although they are almost absent in the miniature tRNAs, these tRNAs mimic an L shape as shown for *R. culicivorax* mt-tRNA^Arg^ by small angle X-ray scattering (233). The distribution and nature of modified nucleotides in organellar tRNA are essential for tRNA structure and activity (239). For instance, m^1^A_9_ has a structural role in human tRNA^Lys^ by participating in the cloverleaf folding of the tRNA (240). m^1^A_9_ is also essential for binding of several T-armless *Ascaris suum* mt-tRNAs on the nematode elongation factor EF-Tu (241).

### Surprising identities in atypical systems

#### Identity elements in atypical tRNAs and TLSs

##### Identity in tRNA^Sec^

These tRNAs allow the incorporation of selenocysteine into selenoproteins in response to a specific stop codon (226). Since there are no corresponding aaRSs, aminoacylation of tRNA^Sec^ by selenocysteine occurs in a two-step indirect process involving in the first step serylation by a standard SerRS and in the second step conversion of seryl-tRNA^Sec^ to selenocysteinyl-tRNA^Sec^ by specialized factors, either selenophosphate synthetases (SelA/B) in *Bacteria* or other factors in a more complex process in *Eukarya* and *Archaea* (242). Consequently, tRNA^Sec^ includes identity elements for recognition by SerRS, selenocysteine factors and other elements that prevent interactions with canonical partners of protein synthesis, notably canonical elongation factors. Many of these identity elements remain unknown.

In *E. coli*, serylation efficiency of tRNA^Sec^ is only ∼1% that of the five canonical serine isoacceptors (226), probably due to its atypical solution conformation distinct from that of tRNA^Ser^ (243). The discriminator base G_73_ in tRNA^Sec^ is the strongest identity element for serylation by *E. coli* SerRS. In addition, the long extra arm and other elements of the atypical secondary structure of tRNA^Sec^ contribute to a lesser extent to the serylation identity. These features are observed in the crystal structure of human SerRS in complex with tRNA^Sec^ (244). On the other hand, three base pairs (C_7_–G_66_, G_49_·U_65_ and C_50_–G_64_) in the core of *E. coli* tRNA^Sec^ are identity elements for SelB recognition and reject the standard elongation factor EF-Tu (245). Mutations in these pairs and elsewhere in *E. coli* tRNA^Sec^ restore recognition by EF-Tu and improve serylation (246). In *Eukarya* and *Archaea*, the second pathway of synthesis of selenocysteinyl-tRNA^Sec^ is dependent on the PSTK phosphorylation factor and the dedicated SepSecS conversion factor (242). However, most of the identity elements that control this pathway remain unknown.

##### Identity elements in viral TLS domains

TLSs are located at the 3′ end of viral genomic RNAs. They are found in several genera of plant viral RNAs and are recognized and aminoacylated (222) by either ValRSs (247), HisRSs (248) or TyrRSs (249). TLSs do not participate in protein synthesis but act in amplification of viral genomes. All TLSs contain the characteristic nucleotide N_–1_ of tRNA^His^, so that the base pair N_–1_–N_73_ can form in all TLSs, including those aminoacylated by ValRS and TyrRS and therefore all are also substrates of HisRS.

The identity of TLSs follows the identity rules of tRNAs and includes determinants mimicking those from the canonical valine, tyrosine or histidine identity sets (222). The discriminator base N_73_ and the anticodon nucleotides are the conserved identity elements in all three plant viral TLS families, but they display functional idiosyncrasies. In the TYMV TLS^Val^ (Figure 4C), the major valylation determinants are A_35_ and to a lesser extent A_73_ when the charging reaction is catalyzed by ValRS of yeast. To comprehensively uncover the full set of valine identity elements in this TLS and assess the role of the pseudoknot in aminoacylation, valylatable variants were selected from a pool of RNA molecules derived from the TYMV TLS^Val^. Selected sequences show strong conservation of C_53_, A_56_ and C_57_ in the pseudo-anticodon loop, but variability is allowed in the length of the L1 loop of the pseudoknot (250). In the presence of wheat germ ValRS, A_35_ and C_36_ of the CAC anticodon together with C_38_ are strong determinants, while A_73_ has no effect on valylation (251). Note that A_73_ and A_35_ are strong valylation identity elements in eukaryal tRNAs^Val^.

The tyrosine identity of bromoviral TLS includes the base A_73_ and anticodon nucleotides (U_35_ and A_36_), in addition to the pair C_1_–G_72_ in the acceptor helix. Interestingly, the tyrosine identity elements in the acceptor helix of TLS^Tyr^ are similar to those of eukaryal tRNA^Tyr^. The contacts seen in the cryo-electron microscopy (EM) structure of BMV-TLS^Tyr^ in complex with TyrRS of *Phaseolus vulgaris* (249) are essentially the same as in the crystal structure of the yeast tRNA^Tyr^:TyrRS complex (108) and involve the catalytic and anticodon-binding domains of TyrRS. However, the topology of the two complexes is different. As seen in the cryo-EM structures, the free BMV-TLS^Tyr^ RNA does not contain a classic L-shaped tRNA mimic. To bind the enzyme, the BMV-TLS^Tyr^ undergoes large conformational changes. The resulting complex resembles the overall configuration of the tRNA^Tyr^:TyrRS complex; however, there is substantially more space between the TLS and the surface of the enzyme (249). The determinants of histidine identity in tobamoviral TLS are the atypical pair N_–1_–N_73_ and two nucleotides that mimic the histidine anticodon nucleotides G_34_U_35_ (252).

The identity of TLSs can be engineered. For instance, replacement of the valine by the methionine anticodon in the TLS^Val^ from TYMV results in an infectious virus with methionine acceptance (222). However, some TLSs are not aminoacylable, such as the one from tymoviral *Erysimum* latent virus (ELV) that lacks a mimic of the valine anticodon loop (253).

##### Identity of tRNA-like structures found in mRNAs

Such RNAs were discovered in the 5′-untranslated regions of mRNAs coding for aaRS (mRNA^aaRS^), first in mRNA^ThrRS^ (254) (Figure 4D) followed by *S. cerevisiae* mRNA^AspRS^ (255). These two TLSs bind to their own aaRS through anticodon loop mimics, thereby regulating the translation of ThrRS and AspRS in processes under the control of tRNA identity rules. Functional and structural studies have discovered the mechanisms that lead to the regulation of the *E. coli* ThrRS gene (*thrS*) (232,256). Using tRNA identity rules, it was possible to switch the specificity of the translational control from ThrRS to MetRS by changing the threonine anticodon-like sequence to the methionine anticodon-like sequence (257). This happens because both ThrRS and MetRS recognize nucleotides in their anticodon loops, thereby allowing the switch of control of the mRNA. A more complex mechanism explains regulation of yeast AspRS by mRNA^AspRS^. Here, the N-terminal extension of each AspRS subunit anchors mRNA^AspRS^ to AspRS via two distinct motifs, connected by a linker sequence, one mimicking the aspartate anticodon domain (255). Recently, several other yeast aaRSs (MetRS, GluRS, ValRS, GlnRS and HisRS) have been found to bind their own mRNAs at anticodon-like structures (223), suggesting that this type of regulation is widely used. Consistently, all these enzymes recognize their tRNAs through identity elements located in the anticodon.

Another TLS fold, called mascRNA, is a small cytoplasmic RNA derived from a long non-coding MALAT RNA that mediates multiple processes in mammalian cells. Once processed by tRNA-specific enzymes, mascRNA folds in a quasi-perfect cloverleaf, and interacts with GlnRS in the multi-aaRS complex and contains tRNA^Glu^ identity determinants (258). However, mascRNA lacks a conserved anticodon loop, is inactive in aminoacylation and does not compete with tRNA^Gln^ for binding on GlnRS. MascRNA enhances global protein translation by increasing GlnRS stability and therefore provides a new paradigm for TLSs to regulate protein levels.

#### Identity rules in atypical aaRS systems

Five amino acid specificities appear in this group of aaRSs. These are monomeric class Ib LysRS (LysRS-1) and ND-GluRS, the dimeric α_2_ class IIb ND-AspRS and α_2_ class IIc PylRS, and the tetrameric class IIc SepRS. They are unevenly distributed in the phylogenies, particularly in archaeal and bacterial phyla (15).

##### Identity elements in tRNA^Lys^ for LysRS-1

Atypical LysRS-1 is found mainly in *Archaea* and some *Bacteria* (in contrast to the standard class IIb LysRSs, which are present in all *Eukarya*, many *Bacteria* and some *Archaea*). To date, only the identity of the tRNA^Lys^ lysylation of the *Borrelia burgdorferi* spirochete is known. While the anticodon bases U_35_ and U_36_ are determinants for both class I and class II LysRSs, the strength of U_36_ is more important for LysRS-1. In contrast, the discriminator base A_73_ plays a marginal role, but the nearby G_2_–U_71_ pair is essential for lysylation by LysRS-1. This pair is also an antideterminant that affects lysylation of *E. coli* class II LysRS (Table 3). Finally, the structural context of the acceptor stem is crucial, since a shift of the wild-type identity pair G_2_–U_71_ to another position in the acceptor stem has dramatic effects on lysine charging by *B. burgdorferi* LysRS (210).

##### Identity elements of tRNAs for ND-AspRS and ND-GluRS

AsnRS and GlnRS genes are missing in many bacterial and archaeal genomes. To overcome the absence of these enzymes, asparaginyl-tRNA^Asn^ or glutaminyl-tRNA^Gln^ are produced via a two-step indirect process. First, a non-discriminating AspRS (ND-AspRS) aspartylates tRNA^Asp^ and tRNA^Asn^,and a non-discriminating GluRS (ND-GluRS) glutamylates tRNA^Glu^ and tRNA^Gln^. Second, the mischarged aspartyl-tRNA^Asn^ and glutamyl-tRNA^Gln^ are amidated in a dynamic supramolecular ternary complex (aaRS:tRNA:AdT) called the transamidosome (259), which comprises from five up to 14 macromolecular entities. Its mechanism is explained by the crystal structures of archaeal-type asparagine and glutamine transamidosomes from *T. thermophilus* (260) and *Thermotoga maritima* (261).

Quite similar identity sets exist in tRNA^Asp^/tRNA^Asn^ and tRNA^Glu^/tRNA^Gln^ pairs (i.e. major determinants in anticodon triplets and discriminator base). In the case of the pair tRNA^Asp^/tRNA^Asn^, the anticodon determinant C_36_ in tRNA^Asp^ is replaced by U_36_ in tRNA^Asn^. Consequently, the dual aminoacylation by ND-AspRSs is based primarily on the idiosyncratic features of their anticodon-binding domains. Similar considerations explain the dual glutamylation of tRNA^Glu^ and tRNA^Gln^ by ND-GluRSs. In addition, the identity sets for aminoacylation of tRNA^Asn^ or tRNA^Gln^ must co-exist with the identity elements for tRNA-dependent amidotransferases. The specificity of aspartyl-tRNA^Asn^ amidation is conferred by the pair U_1_–A_72_ in tRNA^Asn^ and prevented in aspartyl-tRNA^Asp^ by its pair G_1_–C_72_ and U_20a_ in the D loop that act as antideterminants (262). Although the same combination of nucleotides also determines specific tRNA^Gln^-dependent formation of glutamine, the situation is more intricate in glutamine transamidosomes.

*Helicobacter pylori* and several other bacterial species possess two genes of GluRS. GluRS1 aminoacylates tRNAs^Glu^ isoacceptors, while GluRS2 only misacylates tRNA^Gln^ to form glutamyl-tRNA^Gln^ (86,87). GluRS2 recognizes major identity elements clustered in the tRNA^Gln^ acceptor stem. An intermediate case is *Acidithiobacillus ferrooxidans* GluRS1 which charges the two tRNAs^Glu^ isoacceptors and the tRNA^Gln^ species while GluRS2 preferentially charges tRNA^Gln^. It appears that GluRS1 charges exclusively tRNAs with augmented D stem length and a deletion of nucleotide 47, which strongly suggests that these are the major identity elements for GlnRS1 (86). A similar situation could occur in mitochondria, as suggested by the discovery of mitochondrial ND-aaRSs in human fungal pathogens (263).

##### Identity of tRNA^Pyl^ for PylRS

The biology of the atypical PylRS/tRNA^Pyl^ system as present in *Archaea* and a few *Bacteria* is well documented (264). All tRNAs^Pyl^ possess an amber CUA stop codon, but behave towards bacterial elongation factors as typical elongator tRNAs (227). The major identity elements that guide charging are the discriminator base G_73_ and the G_1_–C_72_ pair in the acceptor stem (265,266). However, these determinants are not fully conserved in all tRNAs^Pyl^ and can be A_73_ in *H. volcanii* or U_73_ in tRNA^Pyl^ in some other bacterial species (267). In the extreme halophilic deep-rooted methanogen *Candidatus Methanohalarchaeum thermophilum*, two PylRS/tRNA^Pyl^ pairs are simultaneously present. The two pairs exhibit mutual orthogonality enabled by unique features. A G_73_ discriminator base specifies the identity of tRNA_1_^Pyl^ whereas in tRNA_2_^Pyl^ it is a A_73_ base. In addition, PylRS2 has evolved with a shorter motif 2 loop than PylRS1, which ensures the recognition specificity of A_73_ (268).

Pyrrolysine identity does not rely on anticodon determinants (Figure 4B) (266); therefore, the anticodon of tRNA^Pyl^ can be mutated to recognize codons other than UAG without affecting tRNA recognition by PylRS. Because of its high suppression efficiency, the PylRS/tRNA^Pyl^ system has been widely used for the construction of orthogonal aaRS/tRNA pairs with novel amino acid specificities to expand the genetic code. The crystal structure of the tRNA^Pyl^:PylRS complex from *Methanosarcina hafniense* revealed that the anticodon branch of tRNA^Pyl^ does not contact PylRS, which is the basis for orthogonality engineering required for the synthesis of proteins encompassing abiotic amino acids (269).

##### Identity of tRNA^Ser^ for SepRS

Synthesis of cysteinyl-tRNA^Cys^ in the methanogenic *Archaea* missing CysRS is SepRS dependent. These organisms use a two-step route that is only partially elucidated (270,271). In *M. jannaschii*, phosphoserine is first charged on tRNA^Cys^ by SepRS and subsequently transformed to cysteine by SepCysS (Sep-tRNA^Cys^-tRNA synthase). Interestingly, *M. jannaschii* SepRS differs from CysRS by recruiting the m^1^G_37_ modification as an aminoacylation determinant (272).

In *M. mazei*, one of the few *Archaea* encoding CysRS, serylation of tRNA^Cys^ requires m^1^G_37_ that is a serylation determinant for both SepRS and CysRS. Aminoacylation kinetics reveal that *M. mazei* SepRS and CysRS prefer distinct tRNA^Cys^ isoacceptors with specific determinants in their core region (201). Sequence analysis of tRNAs^Cys^ suggests that G_37_, A_47_ and A_59_ are additional minor identity elements specific to SepRS in addition to the strongest identity elements common to those of CysRS which are the anticodon G_34_C_35_A_36_ and the discriminator U_73_ and the weaker determinants G_15_ and A_47_ (273). Such features are in line with sequence variability of tRNA^Cys^ in the three domains of life and are consistent with the idea that tRNA^Cys^ identity is an ancient RNA record that depicts the emergence of the universal genetic code before the advent of modern aminoacylation systems (273).

### Identity elements in organellar tRNAs are partially conserved

Understanding the identity rules explaining the specificity and efficiency of organelle tRNA aminoacylation is still a largely open field. In mt-tRNAs, identity elements have been experimentally validated in 13 of the 20 mt-tRNA families in a dozen taxa (Supplementary Table S1). To date, the identity of chloroplast and apicoplast tRNAs has been little studied. These tRNAs are structurally close to canonical tRNAs, but with some deviations from canonical sequences. For instance, atypical features are found in plant chloroplast tRNAs (e.g. tRNA^Leu^, tRNA^Ser^ and tRNA^Tyr^), revealing relatedness to cyanobacterial tRNAs, including tRNA^Met^ and tRNA^Ile^ (274,275). In *P. falciparum*, the apicoplast tRNAs fold in canonical cloverleaves but have a nucleotide composition typical of mt-tRNAs (276). Identity elements are only known in *P. falciparum* tRNA^Tyr^, and the field of identities in chloroplast tRNAs remains unexplored.

As a rule, organellar tRNAs can be aminoacylated by bacterial, but not by eukaryal aaRSs, suggesting a relationship between organellar and bacterial identity elements. Identity determinants were first deduced by sequence comparison between bacterial and mt-tRNAs as, for example, for the 22 human mt-tRNAs (277) and other mt-tRNAs from many taxa (278). Due to the great structural diversity of mt-tRNAs, the search for identity elements has naturally been carried out on these idiosyncrasies, especially in *Mammalia* (278). The discovery of structural deviations of mt-tRNAs from canonical cloverleaf folding occurred first in human and bovine mt-tRNA^Ser^ (279) and then in nematodes (234). It was followed by the discovery of the first pathogenic mutations in human mt-RNAs (280).

Seven mt-tRNA identities (arginine, leucine, tyrosine, alanine, serine, aspartate and phenylalanine) and the identity of apicoplast tRNA^Tyr^ have been studied in detail. Partial data are available for tRNAs specific for Ile, Lys, Trp and His. All known identity elements are listed in Supplementary Table S2.

#### Mt-arginine identity

Mitochondrial arginine identity was investigated in tRNAs from the eumetazoan clade, notably in insects and sponges (281,282). For mt-tRNA^Arg(UCG)^ from the coleopteran insect *Caryedes brasiliensis*, the identity set for mt-ArgRS is restricted to the sole U_34_ and C_35_ anticodon determinants. However, in the dipteran *D. melanogaster*, transplantation of the arginine UCG anticodon into the structurally dissimilar mt-tRNA^Asp(GUC)^ led to very low arginine acceptance. Complete transfer of arginine identity could only be achieved with transplantation of the entire anticodon arm of tRNA^Arg^ into tRNA^Asp^, suggesting that the arginine identity is sensitive to specific structural features and to m^1^A and other modified nucleotides present in mt-tRNA^Arg^.

In eumetazoan organelles, the AGR (Arg) codons of the standard genetic code are reassigned to serine/glycine/termination. In *C. brasiliensis* and other evolutionarily related insects, the mitochondrial AGR codons are translated by tRNA^Ser(UCU)^. Investigation of the arginine-accepting activity of tRNA^Ser(UCU)^ from *C. brasiliensis* and other insects of related taxa revealed that the AGR reassignment does not result in arginine misaminoacylation and therefore the mitochondrial protein synthesis is not compromised by misincorporation of Arg residues. In other organisms, whose mitochondrial translation is dictated by the universal genetic code (e.g. *Porifera*/sponges), recognition of the two tRNA^Arg(UCG/UCU)^ resembles that of the yeast cytoplasmic system. The analysis of mt-tRNA^Arg^ variants representative of seven metazoan phyla shows that despite variations in secondary structures, the nucleotides and conformational identity elements (U_34_, C_35_ and anticodon environment) have been largely conserved in *Eumetazoa* that share the AGR codon reassignment but are complemented by specific structural features and/or by modified nucleotides present in insect mt-tRNAs^Arg^ (e.g. m^1^A or Ψ residues) (282).

#### Mt-leucine identity

As for *E. coli* tRNA^Leu^, the identity of the unmodified human mt-tRNA^Leu(UUR)^ transcript is essentially specified by A_73_ and A_14_. Recognition by the mitochondrial LeuRS occurs despite the absence of a long variable arm and, surprisingly, with a partially unfolded anticodon branch, as revealed by probing the structure in solution. This floppy structure is the result of mismatched base pairs and the absence of modified bases in the transcript. Replacement of these mismatches in the anticodon arm by G–C base pairs restores the expected cloverleaf and improves the leucylation efficiency to a level similar to that of native mt-tRNA^Leu(UUR)^. These results suggest that mitochondrial LeuRS contacts mt-tRNA^Leu(UUR)^ in the acceptor and anticodon stems and in the D loop, what has never been observed in any leucine aminoacylation system, and shows a contribution of nucleotide modifications to structure and identity of the tRNA (283,284).

#### Mt-tyrosine identity

Human mt-TyrRS exhibits characteristics of both bacterial and archaeal TyrRS (285). Therefore, mt-TyrRS aminoacylates a tRNA^Tyr^ with a G_1_–C_72_ base pair as efficiently as with an inverted C_1_–G_72_ pair. This is the first example of TyrRS lacking specificity with respect to N_1_–N_72_. This is due to the sequence of the mitochondrial enzyme, which has dual sequence features characteristic of bacterial and archaeal TyrRSs in the region recognizing the N_1_–N_72_ pair. Thus, human TyrRS disobeys general tyrosine identity rules, a behavior probably conserved in *Vertebra* as suggested by the phylogeny (285).

#### Mt-alanine identity

The first three base pairs of the acceptor stem (G_1_–C_72_, G_2_–C_71_ and G_3_·U_70_) and the discriminator A_73_ base are conserved in all known tRNA^Ala^ sequences from *Prokarya*, *Archaea*, eukaryal cytoplasm, chloroplast and plant mitochondria. However, strict conservation is not observed in all tRNA^Ala^ of animal and insect mitochondria. Thus, every animal mt-tRNA^Ala^ identified so far contains at least one sequence variation in these three base pairs (121,131), as found in *C. elegans* mt-tRNA^Ala^ where the G_1_–C_72_ pair is replaced by a G_1_·U_72_ pair (132). This results in *C. elegans* mt-tRNA^Ala^ carrying the identity pair G_3_·U_70_, the G_2_–G_71_ pair but not the G_1_–C_72_ pair replaced by G_1_·U_72_, being charged by *C. elegans* mt-AlaRS but not by *E. coli* AlaRS because of this G_1_·U_72_ pair that acts as an antideterminant (132). On the other hand, *C. elegans* mt-AlaRS charges both bacterial and mitochondrial microhelices, indicating that the G_3_·U_70_ pair remains active in a variety of structural contexts. Separate experiments confirmed that helix instability or irregularity in the acceptor stem is not important for the recognition of the G_3_·U_70_ pair by mt-AlaRS (121). In *D. melanogaster*, mt-tRNA^Ala^ has a G_2_·U_71_ but not a G_3_·U_70_ pair. In this case, the translocated pair G_2_·U_71_ and the pair G_3_–C_70_ are the two main identity elements for aminoacylation by *D. melanogaster* mt-AlaRS (131,132). A similar translocation of the G_3_·U_70_ identity pair to the 5–68 position is observed in human mt-tRNA^Ala^ (133). Furthermore, three weak identity pairs (A_1_–U_72_, A_2_–U_71_ and G_4_–C_69_) complete the identity set, suggesting that the interaction between human mt-AlaRS and its cognate mt-tRNA^Ala^ is different from that in other mt-alanine systems (133). A recent study suggests that because of high rates of mt-DNA sequence evolution in bilaterian animals, mt-tRNA genes have accumulated mutations at significantly higher rates than their cytoplasmic counterparts, resulting in foreshortened and fragile mt-tRNA structures. To compensate for this reduced structural complexity in mt-tRNAs and the loss of identity elements, bilaterians have developed sequence-independent induced-fit adaption mechanisms between cognate mitochondrial aaRSs and tRNAs. This would explain the loss of the G_3_·U_70_ determinants in the acceptor stem replaced by the U–A and G–C pairs (286).

#### Mt-serine identity

The T loop region is the main determinant for serylation and recognition by mt-SerRS in the bizarre animal mt-tRNA^Ser(AGY)^ (279). Data on serine identity of the two serine mt-isoacceptors were collected on *B. taurus* and human mt-tRNA^Ser^ isoacceptors. While mt-tRNA^Ser(UGA)^ has a standard cloverleaf structure, mt-tRNA^Ser(GCU)^ is missing the D arm. The striking feature of these two tRNAs is the absence of the long extra arm, which is the major identity element in cytosolic tRNA^Ser^. Instead, mammalian mt-SerRSs recognize the distinct shapes of the two mt-tRNAs by kinetic discrimination (287,288). In isoacceptor mt-tRNA^Ser(UGA)^, the T loop and the interaction between T and D loops are required, while in the truncated mt-tRNA^Ser(GCN)^, the T loop alone is sufficient with A_57_ and A_58_ as major identity elements (289). This implies a new tRNA binding mode to SerRS different from that in *Bacteria* and *Eukarya* and accounts for a dual-mode recognition employed to discriminate the two mt-tRNA^Ser^ by alternative interaction sites (290). Shape recognition of the D-armless human mt-tRNA^Ser(GCU)^ was recently visualized in cryo-EM structure with human mt-SerRS (291).

#### Mt-aspartate identity

In *H. sapiens* mt-tRNA^Asp^, the set of major elements of aspartate identity is restricted to only the three anticodon nucleotides, with U_35_ and C_36_ being the strongest and G_34_ being a moderate element (292,293). These elements are completed by structural determinants, notably the A_9_:A_12_–U_23_ triple interaction, since its disruption in the tRNA^Asp^ core leads to a huge loss of aspartylation efficiency (294). In addition, changes in the conformation of the anticodon loop can modulate the aspartylation activity. Mutation of A_38_ (a minor determinant) to G_38_ leads to a decrease in activity, whereas C_38_ or U_38_ mutations increase it (292). However, the replacement of G_73_ by A_73_ in human mt-tRNA^Asp^ is surprising considering that G_73_ is a major identity determinant of the aspartate system in all domains of life, including organelles, except in mammalian mitochondria. This is the consequence of a structural idiosyncrasy of mitochondrial AspRS in the peptide binding the acceptor end of human mt-AspRS (293). In marsupials, the mitochondrial aspartate identity is closely related to the glycine identity (see below).

#### Mt-aspartate/glycine identity in marsupials

The marsupial opossum *Monodelphis domestica* contains in its mitochondrial genome a typical tRNA^Asp^ gene carrying the glycine anticodon GCC. After transcription, the incorrect anticodon is edited so that ∼50% of the transcripts carry the GUC sequence while the rest carry the GCC sequence GCC (295,296). The edited form functions as tRNA^Asp^ whereas the unedited form serves as a tRNA^Gly^ (297). This is a unique case of a single tRNA gene that produces two tRNAs with different decoding specificities.

#### Mt-phenylalanine identity

Human mt-PheRS exhibits a minimalist monomeric structure. The enzyme exhibits a broad specificity for bacterial, eukaryal, chloroplastic and mitochondrial-derived tRNA^Phe^ transcripts. The crystal structure of the human mitochondrial tRNA^Phe^:PheRS complex shows that mt-PheRS possesses a relatively simple recognition mechanism of tRNA (298). Unlike *E. coli* PheRS which recognizes an identity set scattered throughout the L-shaped tRNA (see above) (197), human mt-PheRS recognizes a restricted set consisting primarily of nucleotide C_74_ (from the CCA), the pair G_1_–C_72_ and the discriminator base A_73_. Recognition of the anticodon nucleotide G_34_ requires a significant rearrangement of the anticodon-binding domain (298). This recognition mode is probably preserved in *Mammalia*. Furthermore, m^1^A_9_ is an identity element of phenylalanine in *A. suum* mt-tRNA^Phe^ (241).

The yeast mitochondrial PheRS, which is also a small monomeric enzyme, recognizes nucleotides from the anticodon and the acceptor end, including base A_73_ and the adjacent G_1_–C_72_ base pair or at least the C_72_ base. The small size of the monomeric yeast mitochondrial PheRS does not allow contacts with nucleotide 20 at the top corner of the L-shape as there are with the cytosolic enzyme of yeast, *A. pernix* and *E. coli* (299).

#### Diverse and poorly understood identities of mitochondrial tRNAs

In *H. sapiens* mt-tRNA^Ile^, five modified nucleotides (m^1^G_9_, m^2^_2_G_26_, Y_27_, Y_28_ and t^6^A_37_) collectively contribute to isoleucine identity since the unmodified transcript is less active than native mt-tRNA^Ile^ for isoleucylation by human mt-IleRS. Furthermore, A_7_ in the acceptor stem and A_59_ in the T loop are determinants of isoleucine identity, as suggested by two mutations of these residues that cause human pathologies (300).

Human mt-tRNA^Lys^ deprived of modifications (m^1^A_9_, m^2^G_10_, Ψ_27_, Ψ_28_, tm^5^s^2^U_34_ and t^6^A_37_) folds in an inactive extended bulged hairpin that recovers activity and canonical folding upon insertion of m^1^A at position 9. This reveals the major contribution of m^1^A_9_ in the identity of lysine and the L-shaped folding of this mt-tRNA and indirectly to its identity (240). The T-armless mt-tRNA^Met^ of *A. suum* also requires m^1^A_9_ for efficient methionine acceptance (241).

The aminoacylation of *Oryza sativa* mt-tRNA^Trp^ by TrpRS of human and *B. subtilis* reveals large changes in catalytic efficiency resulting from the presence of identity elements specific to the two taxa. However, the rice mt-tRNA^Trp^ identity elements are found in typical positions and include G_73_, G_1_–U_72_ and U_5_–A_68_ in the acceptor stem (301).

*Caenorhabditis elegans* is one of the few organisms that exhibits mt-tRNA^His^ that lacks nucleotide –1. Despite the lack of this canonical His identity element, mt-tRNA^His^ can be efficiently aminoacylated *in vivo*. However, *C. elegans* HisRS still prefers tRNA^His^ with G_–1_ but tolerates tRNA^His^ without G_–1_. Additionally, the results show that the anticodon has taken a leading role in the identity of tRNA^His^ instead of the almost universal –1 determinant (302).

#### Tyrosine identity in apicoplasts

In *P. falciparum*, apicoplast tRNAs fold into canonical cloverleaves but with a nucleotide content typical of mt-tRNAs (276). The identity nucleotide set of apicoplast tRNA^Tyr^ is limited to only three weak identity elements for tyrosylation by cognate *P. falciparum* apicoplast TyrRS, namely G_34_ and U_35_ in the anticodon and the long variable region. The identity element commonly found in the acceptor stem of tRNA^Tyr^, notably the pair G_1_–C_72_ in *Bacteria*, is replaced by A_1_–U_72_ in apicoplast tRNA^Tyr^. This probably results from the high AT content of the *Plasmodium* apicoplast genome and the resulting mutational pressure on the tRNA^Tyr^ gene. How efficiently and specifically tRNA^Tyr^ is aminoacylated in the apicoplast could be explained by the presence of additional antideterminants in tRNA^Tyr^ or additional idiosyncratic peptides in TyrRS (303).

## MECHANISTIC AND EVOLUTIONARY ASPECTS OF THE tRNA AMINOACYLATION REACTION

### Minimal structural requirements of tRNAs and modes of recognition by aaRSs

Positive identity elements for aminoacylation of cytosolic and/or organellar tRNAs have been searched and validated by functional assays in ∼35 taxa covering the three domains of life (Supplementary Table S1), but these taxa are unevenly distributed throughout the phylogeny. Only a few validated identity elements for tRNA aminoacylation are conserved in evolution. Most are concentrated in the distal parts of tRNAs: the amino acid acceptor branch and the anticodon (Table 4). All are strong identity elements. The number of phylogenetically conserved identity elements increases if the analysis is limited to bacterial and eukaryal elements (Table 1), showing the particular status of *Archaea* in evolution.

**Table 4.** Experimentally validated positive identity elements that are almost strictly conserved in all three domains of life including organellar tRNAs and viral TLSs^a^

tRNA families	tRNA domains		
	Acceptor branch	Core region	Anticodon branch
**Cys**	U_73_	…	G_34_, C_35_, A_36_
**Ile**	…	…	A_35_
**Leu**	A_73_	largeVR	…
**Met**	A_73_	…	…
**Val**	A_73_^b^	…	A_35_^b^
**Trp**	…	…	C_34_, C_35_, A_36_
**Tyr**	A_73_^b^	…	G_34_, U_35_
**Ala**	G_3·_ U_70_	…	…
**Gly**	G_3_–C_70_	…	C_35_, C_36_
**Pro**	…	…	G_35_, G_36_
**Thr**	G_1_–C_72_	…	G_34_, U_36_
**Phe**	A_73_	…	G_34_, A_35_, A_36_

Of the 20 tRNA families designated by their amino acid identity, eight do not contain conserved determinants (Arg, Gln, Glu, His, Ser, Asp, Asn, Lys). The position of the determinants in the sequence of the tRNA acceptor branch, the core region and the anticodon branch is shown with standard numbering. ^a^Rare exceptions can be found at certain positions such as G_3_·U_70_ in organellar tRNAs^Ala^, or G_34_ in eukaryal tRNAs^Thr^; ^b^determinants in TLSs. ‘…’ no data available. VR, variable region.

If it is difficult to find common elements of identity, it is equally difficult to find a universal tRNA scaffold as the diversity of aminoacylable structures is great. For instance, in viral tRNA-like structures, the amino acid acceptor arm contains a pseudoknot and only the terminal -N_73_CCA_OH_ sequence and the N_1_ residue are conserved. It has been known for a long time that a canonical acceptor arm is not mandatory for activity, since a fragment of yeast tRNA^Phe^ with an excised 5′ quarter is efficiently aminoacylated by yeast PheRS provided the m^7^ group of G_46_ is removed (304). Likewise, a poly(U) (∼30 U residues) with an attached 3′-NCCA_OH_ is aminoacylated by a mammalian LysRS (305). These mimics exhibit conformational flexibility and contain key anticodon identity determinants. In this context, it is noteworthy that arachnids contain remarkably short and unusual mt-tRNAs (235,306). Sequencing of mitochondrial genomes from several genera of spiders revealed that most tRNAs cannot be folded into a classical four-armed cloverleaf secondary structure. Most tRNAs lack the D or T arms, and at least four of them lack both D and T arms. In addition, the acceptor stems have multiple mismatches. In fact, the 39 bp tRNA^Ser1^ gene from *Parachtes romandiolae* is 3 bp shorter than the tRNA^Arg^ from the nematode *Romanomermis culicivorax*, setting a new record for the shortest tRNA gene ever described (235).

**Figure 6.**
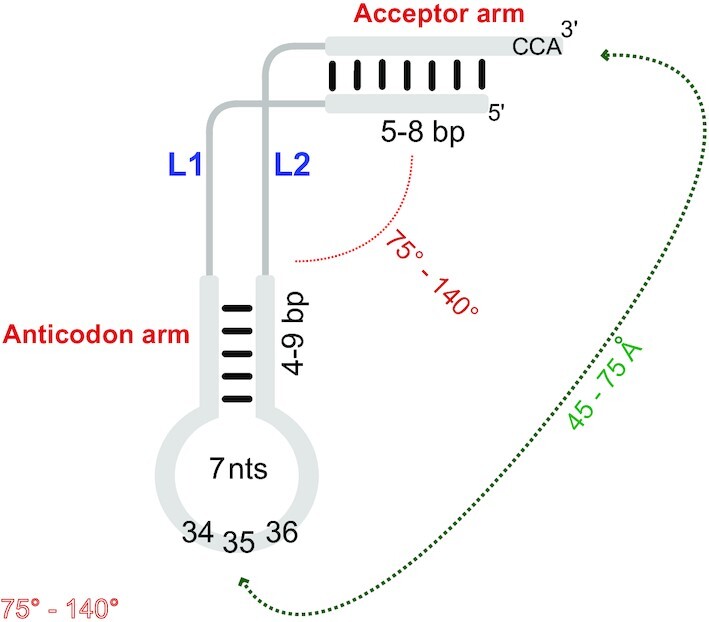
Generalized L-shaped structure of RNAs recognized and/or aminoacylated by aaRSs with structural requirements necessary for function. The drawing schematizes how the amino acid acceptor (7 bp) and anticodon (5 bp) branches are connected in canonical tRNAs by two linkers L1 (from U_8_ to N_26_ with the D arm) and L2 (N_44_ to N_48_ with the T arm and either the small or large variable region). In atypical and mt-tRNAs, this architecture shows important peculiarities, with distal stem regions of 5–8 and 4–9 bp for acceptor and anticodon branches, respectively, pseudoknotted acceptor stems and great diversity in L1 and L2 sequences. The conserved pairings are shown. The orientation of the two arms is variable and the distance between the CCA_OH_ end and the anticodon triplet ranges between 45 and 75 Å.

This highly diverse and aminoacylable set of RNAs leads to an L-shaped tripartite structural model (Figure 6) with a central region connecting the amino acid acceptor and anticodon branches. For translation, a 7 nt anticodon loop and a terminal 3′-NCCA_OH_ are mandatory, but other tRNA regions may show high variability. The minimalist and necessary structural and functional requirements for tRNA aminoacylation can be summarized as follows.

- For aminoacylation and more generally for ribosome-dependent protein synthesis, aaRSs recognize identity sets mainly located at the two distal ends of the tRNA.
- AaRSs are platforms that anchor the RNAs in adapted active conformations.
- Generally, mitochondrial aaRSs recognize minimalist identity sets in mt-tRNAs and sometimes altered sets, such as in metazoan animals, that can be restricted to elements located solely in the acceptor branch or the anticodon loop.
- The architectural frameworks in mt-tRNAs and atypical tRNAs play crucial functional roles. Evolution adapted identity rules to the intrinsic conformational fragility of aminoacylable RNAs.

A simplistic view of the mechanism of identity expression would be that direct electrostatic interactions made by aaRS with discriminating functional groups on tRNA determine the specificity of aminoacylation following the general lock-and-key model of interaction. Although true to some extent, an additional mechanism of indirect reading completes the expression of identity. Revealed by structural and functional studies and called the ‘indirect readout’ mechanism, this mechanism senses sequence-dependent conformations of tRNA upon recognition of aaRS by contacts with the sugar–phosphate backbone and non-specific parts of the bases (307). In other words, the indirect readout suggests that the distribution of nucleotides is not random outside the set of identity elements. This explains previous studies showing that nucleotide changes outside of recognition sets are not neutral for aminoacylation, as shown, for example, for yeast tRNA^Phe^ (308) or yeast tRNA^Tyr^ (309).

### Interactions with aaRS during tRNA aminoacylation

#### Direct contacts with identity elements of tRNA

**Figure 7.**
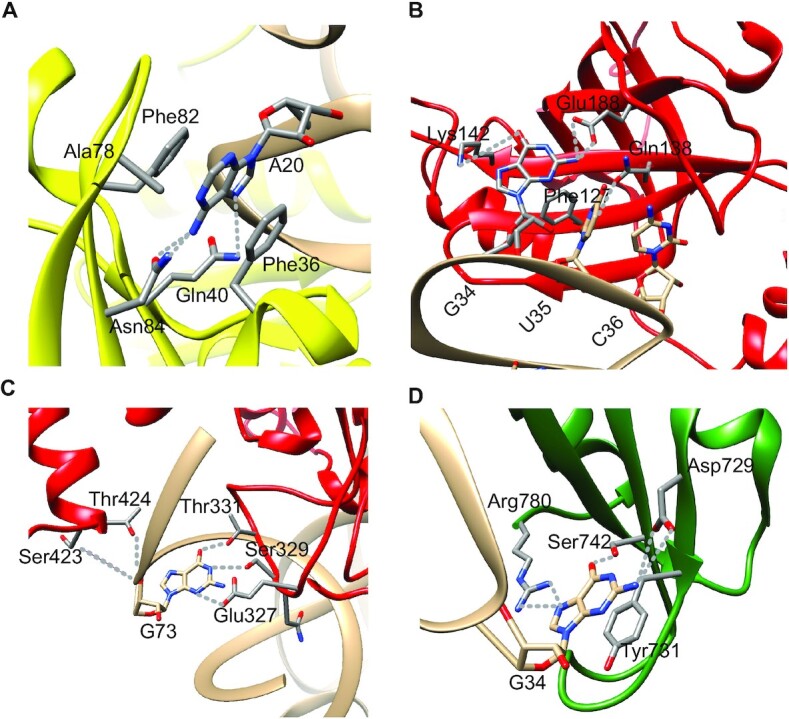
Examples of interactions between major identity determinants in tRNA with cognate aaRS as seen in crystal structures of tRNA:aaRSs complexes. (**A**) A_20_ in the *E. coli* tRNA^Arg^:ArgRS complex (5b63). (**B**) U_35_ and C_36_ in *the S. cerevisiae* tRNA^Asp^:AspRS complex (1asy). (**C**) A_73_ in the *S. cerevisiae* tRNA^Asp^:AspRS complex (1asy). (**D**) G_34_ in the *T. thermophilus* tRNA^Phe^:PheRS complex (1eiy).

Hydrogen bonding and stacking appear to be the straightforward way to recognize tRNA identity determinants during aminoacylation. Figure 7 displays examples of such contacts between strong determinants and aaRSs specifying arginine, aspartate and phenylalanine identities. Thus, A_20_, the strongest arginine determinant in *E. coli* tRNA^Arg^, interacts with five amino acids of *E. coli* ArgRS through a network of direct H-bonds completed by a stacking interaction (Figure 7A). Interestingly, these direct contacts represent the initial recognition step before the intervention of strong anticodon determinants (23). In the yeast aspartate system, the major identity determinants G_34_, U_35_ and C_36_ interact with AspRS via six amino acids by H bonding and Phe127 by stacking (Figure 7B). Moreover, the adenine moiety of the discriminator base G_73_ establishes direct H-bonds with four amino acids of AspRS (Figure 7C) (310). The strongest identity determinant G_34_ of the *T. thermophilus* tRNA^Phe^ makes direct contact with the β-subunit of the anticodon-binding domain B8 of PheRS, notably by H-bonds with three amino acids (Asp729, Ser742 and Arg780) and stacking with Tyr731 (Figure 7D) (311). The minor determinant A_73_ in *T. thermophilus* tRNA^Phe^ does not contact PheRS, but forms intra-tRNA H-bonds with adjacent nucleotides, as seen in the ternary complex with a stable phenylalanyl-adenylate (312). Generally, most of the contact amino acids, especially those stacked on bases, are conserved in aaRSs. Interestingly, in the *T. thermophilus* tRNA^His^:HisRS complex, only the two regions around the major G_–1_ and minor anticodon identity determinants are anchored on HisRS by a network of H-bonds (147).

Sometimes mutations in aaRSs that disrupt interactions with tRNA identity elements are so critical that they induce lethality *in vivo*. A mutation within yeast AspRS (Glu188Lys) is lethal due to the disruption of the interaction of the tRNA^Asp^ identity base G_34_ with the AspRS anticodon-binding β-barrel and thus inactivating the enzyme by relaxing the recognition specificity (217). Similar effects occur with other bacterial aaRSs, for example GlnRS, MetRS and TyrRS; see (4). In *B. stearothermophilus* TyrRS, Glu152 acts by electrostatic repulsion of non-cognate tRNAs (313). On the other hand, amino acids in aaRSs, although they do not make H-bonding contacts with tRNAs, may be critical for the specificity of aminoacylation, as is the case of Leu136 in *E. coli* GlnRS (314).

#### Direct contacts with the backbone of tRNA

It is generally expected that contacts with the standard sugar–phosphate backbone of tRNAs do not contribute to the specificity of recognition (although interactions of the same type can lead to the ‘indirect readout’ phenomenon). This is illustrated by the example of yeast AspRS where half of the tRNA/aaRS interactions do not participate in aspartate identity (310). These contacts are in the acceptor stem, the D stem and the anticodon branch of tRNA^Asp^, and include interactions with phosphate oxygens of the tRNA backbone. Mutation of the protein residues involved in these interactions only led to moderate changes in the affinity for tRNA^Asp^ without relaxation of tRNA recognition when assayed with bulk tRNA. This confirms that they do not contribute to specific recognition but stabilize the tRNA^Asp^:AspRS complex (218). Similar contacts have been described in other systems, for example in the histidine *T. thermophilus* (147) and the *E. coli* arginine (23) systems. This type of interaction is widely used in tRNA:aaRSs complexes and represents more contacts than with the identity elements of tRNAs.

#### Structural adaptations required for recognition of identity elements

Many functional and crystallographic investigations have revealed conformational adaptations in tRNA:aaRS complexes. For example, the G_10_·U_25_ pair is an identity element in the yeast AspRS system despite the absence of interaction with the enzyme, indicating that the pair modulates the conformation and positioning of other identity elements in tRNA (310). On the other hand, the interaction of the tRNA^Asp^ anticodon loop with the yeast AspRS results in a radical change in the L-shape, with the bases spreading in the β-barrel domain of the enzyme (315). This contrasts with *T. thermophilus* PheRS where the conformation of the anticodon loop is relatively similar to that of free tRNA. The only significant difference is that G_34_ is unwound outside the anticodon loop and stacks directly on Tyr731 in the B8 domain of PheRS (311).

As mentioned above, ‘indirect readout’ is an alternative way to specifically recognize tRNA (307). It takes advantage of global and local conformations, sequence elements and contacts with 2′-hydroxyl groups, as has been observed in several tRNA/aaRS systems (34,316). Indirect reading reinforced the importance of water-mediated H-bonds that complement direct macromolecular contacts. This is well illustrated in the complexes of *E. coli* AspRS with its cognate tRNA^Asp^ (317) or with the heterologous yeast tRNA^Asp^ (176), where H-bonds occur almost exclusively in the acceptor stem and often establish contacts with the phosphate oxygens and riboses of the ribophosphate backbone.

### Kinetics in tRNA aminoacylation and allosteric effects

#### Strength of identity determinants

Steady-state kinetics accounting for the strength of identity determinants is well documented (9,318). The strength of the determinants is measured by the parameters *k*_cat_ and *K*_M_, which reflect catalysis and binding, respectively. In general, the *k*_cat_ parameter is most affected in mutational studies. This is illustrated by *E. coli* glutamine (83,85) and yeast aspartate identities (184). For glutamine identity, decreases of *k*_cat_ can reach ∼10^4^-fold upon mutation of anticodon identity determinants, while *K*_M_ does not increase by more than 10-fold. The trend is similar for aspartate identity, although *k*_cat_ is consistently less affected with decreases of at most ∼200-fold. A *K*_M_ dependence characterizes the identity pair G_10_·U_25_ in yeast tRNA^Asp^ and is explained by the absence of contact of this determinant with AspRS (184,310). Similar features characterize the U_3_–A_69_ pair in *E. coli* tRNA^Val^ (73) and the G_2_–C_71_ pair in *A. pernix* tRNA^Trp^ (97). The case of *E. coli* tRNA^Glu^ is remarkable, as the 100-fold reduction in catalytic efficiency of the transcript lacking mnm^5^s^2^ modification on the U_34_ determinant results solely from a *K*_M_ effect, i.e. a loss of affinity (76). *K*_M_ effects are regularly observed in transplanted tRNAs, leading, for example, to a moderate loss of catalytic efficiency when the yeast aspartate identity set is transplanted into the yeast tRNA^Phe^ (184). The effect is much more pronounced when the aspartate set is transplanted in the *E. coli* tRNA^Gln^, and vice versa when the *E. coli* glutamine set is inserted into the yeast tRNA^Asp^ (319). This shows that the minor/major groove recognition modes of the tRNAs specific to each class of aaRS are not locked by the identity switches.

More generally, the conditions under which the aminoacylation parameters are measured *in vitro* can significantly modulate the catalytic efficiencies. One example is the tuning of the efficiency of phenylalanylation by Mg^2+^ concentration, which accounts for the non-identical sets of determinants in the *E. coli* tRNA^Phe^ isoacceptor (199). Similarly, *in vivo* associations of aaRSs affect the efficiency of tRNA charging, as shown by the archaeal associations between LysRS, LeuRS and ProRS (320,321).

#### Allosteric communications in tRNA:aaRS complexes

Structural plasticity is an essential phenomenon occurring throughout the interaction of aaRSs with their substrates (15,322,323). Contacts of aaRSs with amino acid and ATP substrates occur mainly in the catalytic site, while with tRNAs they also occur with distant modules, located up to 75 Å from the catalytic site. These events involve long-distance information transfer across the structural body of aaRSs and/or tRNAs. It is now well established, through decades of experimental and theoretical studies, that aaRSs have adapted their catalytic mechanisms to achieve specificity with moderate affinity for their substrates (tight binding would make product release more difficult and decrease turnover) (324). The objective for aaRSs is to provide the highest affinity for adenylate intermediates that must remain bound to the enzyme as premature release would lead to their hydrolysis in solution and prevent the transfer of amino acids to the tRNA acceptor end. Given this need, allostery works through conformational changes or stabilization of flexible regions during substrate binding. Structural studies have revealed a wide range of substrate-binding modes, from a rigid lock-and-key model to various types of induced fit modes.

Examples of induced fit have been described in yeast AspRS. The ‘flipping loop’ of the AspRS active site, which is disordered in the absence of amino acid, becomes visible upon interaction with aspartic acid in the presence of tRNA (325,326). Differences in the conformation of the two AspRS subunits are also observed after binding of the tRNA nucleotides G_73_ and C_74_ to the catalytic domain (310). Upon binding, additional interactions are established between AspRS and the tRNA^Asp^ anticodon that trigger the rotation of the anticodon and hinge domains, allowing the correct positioning of the 3′ end of the tRNA in the active site. Allosteric changes in the tRNA^Asp^:AspRS complex induce strong anticooperative effects on tRNA^Asp^ mutants between the anticodon identity elements G_34_, U_35_ and C_36_ and the discriminator G_73_, all of which interact with AspRS at a distance of ∼75 Å (327). The triple mutant of the anticodon nucleotides probably loses interactions with the anticodon-binding domain of AspRS since it behaves kinetically as a minihelix^Asp^ (328). Similar effects have been observed in other systems, notably in the proline system of *T. thermophilus* and in the mammalian arginine system. In the ProRS system, changes in the conformation of the Pro- and ATP-binding loops occur during binding, allowing functional binding of tRNA^Pro^ (329). In ArgRS, one of the three aaRSs that requires the presence of tRNA to catalyze the formation of arginyl-adenylate, the A_76_ terminal nucleotide, the D loop and the anticodon arm of tRNA^Arg^ together enable arginine activation in the active site (27). For some aaRSs, the induced adjustment mechanism resulting from substrate binding is probably precise enough not to require additional editing activity.

Currently, long-range allosteric communication pathways are documented in most aaRS families (324). For example, in the *E. coli* tRNA^Cys^:CysRS complex, a communication pathway between the anticodon-binding region and the catalytic site of CysRS has been identified in which specific amino acids provide functional coupling between the two sites and promote active tRNA conformation (330). In the case of the *E. coli* tRNA^Gln^:GlnRS complex, pre-steady-state kinetics on *E. coli* GlnRS mutants revealed allosteric signaling pathways in the enzyme body that regulate glutamine binding and glutaminyl-tRNA formation (331).

### Identity elements in the proofreading activities and ‘quality control’ of the aminoacylation reaction

The inaccuracy of amino acid and tRNA selection by aaRSs explains why the aminoacylation of tRNAs is not entirely specific. Aminoacylation errors increase the diversity of the proteome and appear to be a driving force of evolution (332). Errors are tolerated up to system-dependent thresholds. Too many errors could threaten cellular life and under certain circumstances lead to dysfunctions, which is why aaRSs have evolved correction mechanisms, also known as proofreading or editing mechanisms. Until recently, proofreading was thought to be absent from mitochondria, until the discovery of editing by human mt-AlaRS (333).

The concept of kinetic proofreading was proposed in the 1970s by Hopfield (334) and Ninio (335). It follows the observation of high levels of ATP consumption in the presence of non-cognate amino acids, as first demonstrated for valine mischarging on tRNA^Ile^ by *E. coli* IleRS (334). A two-step editing process was proposed by Fersht comprising pre-transfer editing (first sieve) that clears amino acids misactivated in the catalytic site, and post-transfer editing (second sieve) that edits mischarged tRNAs with the help of dedicated editing domains (336). More recently, *trans*-editing (third sieve) catalyzed by freestanding editing domains has been proposed (337). Currently, the editing mechanism is supported by a body of experimental data and is globally understood (338,339).

#### A balance between pre-transfer and post-transfer editing activities

The question of whether the editing mechanisms of IleRS, LeuRS and ValRS use a tRNA-independent or a tRNA-dependent pre-transfer pathway has been clarified. In *E. coli* IleRS, tRNA-dependent pre-transfer editing accounts for one-third of the total proofreading activity and uses a conserved tyrosine residue in the catalytic site for both editing and aminoacylation. This dual process is kinetically controlled and, in *E. coli* LeuRS, depends almost entirely on post-transfer tRNA editing in which the 3′-OH group of A_76_ in tRNA^Leu^ plays a crucial role (340).

Post-transfer editing deacylates mischarged tRNAs in editing domains distinct from the synthetic aminoacylation sites. This requires conformational changes in both aaRSs and tRNAs. The mechanism involves the use of partially distinct tRNA interactions in the editing and synthetic sites. In the isoleucine system, this results in the segregation of editing determinants in the L-shaped tRNA corner (G_16_, D_20_ and D_21_) while the anticodon of tRNA^Ile^ contains the aminoacylation identity elements (205). Similarly, the anticodon arm of tRNA^Leu^ is essential for LeuRS editing, but dispensable for aminoacylation (63).

In a comprehensive study, the functionality of editing domains was examined by domain exchanges between species. Deletion of the human LeuRS editing domain leads to a complete loss of synthetic activities (activation and transfer), and only the yeast editing domain can partially rescue the different functions of LeuRS. This demonstrates the structural interdependence of the synthesis and editing sites, which ensures the coordination of the two opposing activities (341–344).

Other aaRSs show different partitions of the pre- and post*-*transfer editing activities [for a review, see (339)], but little is known about the identity elements involved in these processes.

#### Editing in trans by freestanding editing proteins

AlaRSs, ProRSs and ThrRSs contain editing domains that are not strictly conserved through evolution and often involve *trans-*editing proteins [AlaXp, ProXp, YbaKp, ThrRS-ed and DTD (d-aminoacyl-tRNA-deacylase (345)]. These autonomous proteins recapitulate the editing function of the corresponding aaRSs. For example, ThrRS-ed proteins are truncated ThrRSs lacking the catalytic site, as found in some *Archaea* (346). ProXp proteins have relaxed specificities and recognize multiple mischarged tRNAs, even with non-proteinogenic amino acids (347). Sometimes a given aaRS mediates the *trans*-editing of its own tRNA mischarged by another aaRS (173). For example, in mammals, tRNA^Thr^ containing base pair G_4_·U_69_ is efficiently alanylated by AlaRS and is edited by ThrRS, thereby preventing the mistranslation of threonine to alanine. Interestingly, *E. coli* ThrRS has cross-editing capability, although alanyl-tRNA^Thr^ is not produced by AlaRS in bacteria (173).

Chiral proofreading catalyzed by DTD proteins deserves some attention (348). DTDs remove d-amino acids mischarged on tRNAs and achiral glycine mischarged on tRNA^Ala^. This chiral *trans*-editing process appears to be ubiquitous. One type of DTD is probably the progenitor of archaeal ThrRS that contains a module homologous to the DTD fold (349). The alanine identity base pair G_3_·U_70_ is a universal determinant for DTD proteins, which explains the functional relationship of DTD with AlaRS. Bacterial DTDs efficiently remove non-cognate glycyl-tRNA^Ala^ but much less so the cognate glycyl-tRNA^Gly^ due to its discriminator base U_73_ which acts as an antideterminant. More generally, the discriminator base N_73_ in tRNA modulates the activity of DTD proteins (348) and may be specific to phyla and organelles (350). In higher *Eukarya* (i.e. *Animalia*), a paralog of DTD with relaxed specificity, named ADT, proofreads alanylated tRNA^Thr^ isoacceptors. ADT acts as a glycine deacylase and hydrolyzes mischarged glycyl-tRNA^Ala^ in *Bacteria* and *Eukarya* (351). Surprisingly, the high level of d-alanyl-tRNA^Ala^ synthesized by *T. thermophilus* AlaRS is not edited by a DTD protein but progressively deacylated by post-transfer editing in the synthetic site of AlaRS. This demonstrates the active role of AlaRS in controlling chirality (352). In summary, chiral proofreading DTD enzymes are a major cellular checkpoint, but other molecules prevent the infiltration of d-amino acids into the translation machinery. They include aaRSs, EF-Tu and ribosomes (348).

Finally, we can highlight the resistance to these proofreading activities of mischarged tRNAs by the non-discriminating aaRSs D-AspRS and ND-GluRS. This resistance is partly due to the channeling of the aminocylation and amidation processes that prevents the premature release of poorly charged products into the cell (260,261).

#### Relaxing the ‘quality control’ mechanism of tRNA aminoacylation can benefit the cell by reinterpreting the genetic code

It is generally accepted that the aaRSs provide the first checkpoint of ‘quality control’ of the translation by ensuring the accurate aminoacylation of tRNAs assisted by the proofreading activity. The accuracy of tRNA aminoacylation is generally considered to be better than 10^−4^, a value comparable with the typical accuracy of ribosome decoding, also of the order of 10^−4^.

These two reactions are the two key steps in maintaining the translational fidelity of the genetic message. In total, high-speed translation of a cell results in a translation error rate of approximately one error for every 10^3^–10^5^ amino acids (353–355). This concept of highly accurate protein synthesis is commonly accepted, as an error in tRNA aminoacylation would lead to ambiguity in the genetic code and produce statistically modified proteins. This concept has been challenged by the discovery in fungi of the *Candida* clade of ambiguous decoding of leucine codons that increases phenotypic diversity (356). New studies have shown that corrupted identities can produce misinterpretation of the genetic code, which can be an advantage under stressful conditions. However, excessive corruptions of identity rules produce toxic effects, especially in higher eukaryotes, leading to metabolic dysfunctions and diseases in humans (357–359). Therefore, controlling the quality of the aminoacylation reaction may lead to antagonistic effects in different cases or circumstances. For example, it has been shown that *E. coli* cells not only tolerate the presence of misacylated tRNAs but may even require them to grow under selective pressure. Up to 10% of mismade proteins are tolerated by *E. coli*, suggesting that the editing function of aaRS is not essential for survival in some circumstances. This triggers a heat shock response that stimulates non-optimized polypeptides to reach a native conformation or to be degraded (360).

On the other hand, a bacterial strain containing a defective PheRS in tyrosyl-tRNA^Phe^ editing exhibits growth limitation when exposed to an excess of non-cognate amino acids and other stresses (361). Mischarging by human AlaRS of non-cognate tRNAs carrying a G_4_·U_69_ pair is beneficial to cells (362) whereas, conversely, errors mediated by *E. coli* AlaRS are not well tolerated and induce a global stress response that leads to a significant perturbation of the proteome, with potential catastrophic effects on fitness and viability (363). In agreement, a mouse with a ‘ticky’ mutation, which results in loss of cerebellar Purkinje cells and ataxia, carries a missense mutation in the AlaRS editing domain that results in low levels of misfolded tRNAs and accumulation of misfolded proteins in the neurons (357). Accordingly, different cell types tolerate different levels of mistranslation which is advantageous under certain physiological conditions due to a diversification of proteomes [for reviews, see (364,365)]. Loss of editing domains and ability to edit mischarged amino acids by LeuRS, ThrRS and PheRS in *Mycoplasma* results in tRNA mischarging, and the resulting mistranslation helps parasites evade host immune responses by increasing antigen diversity (366).

Finally, we cannot fail to mention the ultimate role of EF-Tu in the control of aminoacylated amino acids. The bacterial elongation factor EF-Tu binds to all tRNAs acylated with their correct amino acid (cognate) with almost uniform affinity. This occurs because the sequence of each tRNA has evolved to compensate for the variable thermodynamic contribution of the esterified amino acid. More precisely, each tRNA uses different combinations of three base pairs in the acceptor branch to adjust the affinity of aminoacyl-tRNAs for the EF-Tu elongation factor for optimized interaction (i.e. binding or rejection) with the ribosome (367). These base pairs are conserved in *Bacteria* (including tRNA^Sec^ for recognition by SelB and rejection of EF-Tu), supporting the idea that EF-Tu has a safeguarding role in the quality control process. In conclusion, the control of translation errors strongly contributes to the quality control of the expression of the genetic code (368).

### Comprehensive overview of tRNA identity expression processes

The two-step mechanism of tRNA aminoacylation is currently well understood. However, this mechanism is error prone *in vitro* and *in vivo*, revealing relatedness between identity sets, idiosyncrasies in catalytic processes and functions beyond aminoacylation. Strong identity elements in tRNA make direct and water-mediated indirect contacts with aaRSs, in processes requiring conformational changes in anticodon loops and acceptor termini. Major identity elements are generally conserved. However, idiosyncrasies occur in systems where aaRSs have a complex evolutionary history (AlaRSs, ArgRSs, ProRSs, ND-aaRSs and archaeal aaRSs). For example, AlaRSs use different ways to recognize the identity determinant G_3_·U_70_ (128), and the yeast ArgRS relies on different *in vivo* mechanisms, based on subtle relationships of nucleotides at identity positions 20, 34, 35 and 36 to charge the four tRNA^Arg^ isoacceptors (24). In addition, the use of sequence features outside canonical identity sets to adjust specificity is probably widespread but only occasionally documented (308,309).

The similarity of identity sets of certain specificities implies the possibility of identity changes. Such changes can be engineered *in vitro* by manipulation of identity elements, e.g. (112), and occur *in vivo* due to anticodon shifts (369), but are not obligatory as the identity of tryptophan is not altered in yeast amber suppressors (93).

Some unusual features of organelle tRNA/aaRS systems, such as minimalist or altered identity sets and sometimes miniaturized mt-tRNAs, add a new level of complexity. For example, although the structures of human mt-AspRS and *E. coli* AspRS are similar, the G_73_ discriminator is a major identity determinant in *E. coli* tRNA^Asp^ but not in human mt-tRNA^Asp^, as a consequence of the enlarged catalytic groove, electropositive surface and reduced thermal stability of human mt-AspRS (293). Furthermore, in the human mitochondrial alanine system, the G_3_·U_70_-independent charging of tRNA^Ala^ requires minor elements in the acceptor stem (133) complemented by shape and folding features (308,309). Similar recognition mechanisms in which anticodons would play a crucial role are postulated for the recognition of the miniature tRNAs by their mt-aaRSs.

In conclusion, based on current knowledge, it can be deduced that the mechanism of tRNA aminoacylation by aaRSs is based on (i) plasticity and dynamic functioning of tRNA/aaRS systems with allosteric adaptations; (ii) specificity tuning by elements outside the canonical identity sets; (iii) error-prone mechanisms leading to frequent misactivation of the amino acid and mischarging of the tRNA; and (iv) proofreading strategies to overcome excessive functional errors. Most of these features are the result of an evolutionary process, which implies the current existence of idiosyncrasies in taxa deeply rooted in the tree of life.

## EXPANDING THE WORLD OF tRNA IDENTITY

### Towards cracking a challenging conundrum in life sciences

Sequence analysis by statistical methods provided the first functional signatures in tRNAs (370,371) that were rationalized with the proposal of universal identity rules for tRNA aminoacylation (4). Beyond this historical and fundamental function of tRNAs as essential components of translation, many recent studies suggest that the roles of tRNA in many biological processes go beyond this paradigm (3,372). How to deconvolute the different signatures specific to these functions and encrypted in tRNAs, tRFs (also known as tRNA-related RNA fragments or tRNA fragments) and TLSs is a new challenge (222,373). Finding reliable answers is difficult and requires new algorithms, given the increasing amount of tRNA sequences compiled in databases [∼170 000 for tRNAs, ∼10^6^ for tRNA genes or pseudo-genes, ∼13 000 for tRFs, ∼3000 for mammalian mt-tRNA genes and ∼37 000 for plant photosynthetic tRNA genes (65,164,374–377)] as well as in TLSs and other tRNA-derived molecules (255).

Finally, the very notion of tRNA identity so often associated with the terms aminoacylation, positive and negative determinants and second genetic code (378,379) deserves to be revisited and refined, to include features and functions beyond aminoacylation. In what follows, extensive searches for ‘identity elements’ refer not only to signals that specify tRNA aminoacylation by aaRSs, but also to those that specify other functions of tRNAs.

### Early bio-informatic predictions

The first studies aimed to find identity elements mainly for tRNA aminoacylation. The first computer-assisted comparison of tRNA sequences was conducted on 67 sequences of *E. coli* and *Salmonella typhimurium* tRNAs and showed the importance of anticodon positions 35 and 36 for decoding and putatively for identity (371). Interestingly, a second region in the tRNA acceptor branch was distinguished, including nucleotide 73, and the first four base pairs of the acceptor stem (371). Much later, the first candidate identity elements in archaeal tRNAs were derived from large-scale sequence analysis performed on ∼1100 tRNA genes from 22 archaeal species, mainly *Euryarchaea*, a few *Crenarchaea* and one *Nanoarchaea* (380). These candidate identity elements are found in anticodons, notably at three positions of Asn, Asp, Cys, Gln, Glu, Ile, Met_e_, Phe, Trp and Tyr anticodons, or two positions (Gly, Thr and Val anticodons), or one position in Lys and Pro anticodons. Many predicted determinants are outside the anticodons, notably the G_3_·U_70_ pair in tRNAs^Ala^, the discriminator N_73_, A_20_ in tRNAs^Arg^, various base pairs in stems of cloverleaves of tRNA and the variable region in tRNAs^Leu^ and tRNAs^Ser^. Interestingly, some identity candidates located in stem regions have no known homologs in *Eukarya* and *Bacteria*, such as C_13_–G_22_ and C_50_–C_64_ in tRNAs^Phe^ and tRNAs^His^, respectively. Similarly, some candidates validated by aminoacylation assays (see above) are specific to *Archaea*, such as C_27_–G_43_ in tRNAs^Ile^, G_22_–U_44_ and A_59_ in tRNAs^Leu^, U_11_–A_24_ in tRNAs^Val^, G_29_–C_41_ and C_50_–G_64_ in tRNAs^His^, and the first three G–C pairs on top of the acceptor stem in tRNAs^Pro^. In contrast, the validated pair C_1_–G_72_ in *A. pernix* tRNA^Thr^ was not predicted to be a threonine determinant in the three domains of life (for comparisons, see Table 1). Later predictions conducted on larger sequence sets analyzed by standard routines revealed a co-evolution of tRNA^His^ identity rules (148) and found divergences in identity between tRNAs from *Proteobacteria* and *Cyanobacteria* (381). Other automated methods visualized determinants and antideterminants (212) and suggested a hierarchical organization of these elements according to ‘Class Informative Features’ (382).

### Current bioinformatic-assisted searches of tRNA identity elements

Several bioinformatics studies have aimed to identify tRNA sequence elements characteristic of aaRS classes (383) or to predict identity elements for aminoacylation (384,385). Surprisingly, some of the predicted identity elements disagreed with known aminoacylation data, suggesting that they were not associated with recognition by aaRSs (385) but could act as antideterminants to prevent false recognition (386,387). Alternatively, they could encode identities unrelated to tRNA aminoacylation and contribute to the recognition of other tRNA-binding proteins, such as base-modifying enzymes (see below).

### Ab initio *searches of identity elements*

#### Global search in tRNA sequences from the three domains of life

**Figure 8.**
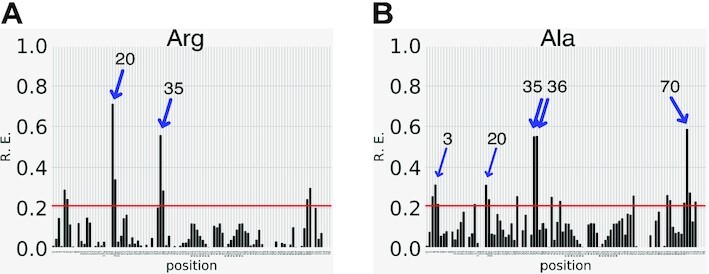
Examples of position importance in bacterial tRNAs for (**A**) arginine and (**B**) alanine identities. Importance of positions in tRNA sequences is quantitatively evaluated by relative entropy (RE) values. Adapted from Branciamore, S. *et al.* (2018) Intrinsic properties of tRNA molecules as deciphered via Bayesian network and distribution divergence analysis. Life (Basel), 8, E5. (385). Profiles in (A) and (B) are adapted from panels c of Supplementary Figures S1 and S2 of reference (385). Arrows have been added to highlight the positions with the highest RE values associated with aminoacylation identity. According to the CC BY 4.0 license (https://creativecommons.org/licenses/by/4.0/).

This was done from a set of ∼10^4^ tRNA sequences analyzed by probalistic Bayesian methods (164,385). The predicted identity positions were classified by amino acid specificities in each of the three domains of life. This gives, for each tRNA family, unique ‘importance’ profiles, as illustrated for two patterns specifying arginine and alanine identity in *Bacteria* (Figure 8). The profiles conform to previously identified identity elements. Indeed, the most important positions 20 and 35 are occupied by the major A_20_ and C_35_ identity elements in bacterial arginine systems (Figure 8A). Likewise, the alanine profile confirms the identity elements at positions 3:70 and 20. The profile also highlights positions 35 and 36 of the anticodon, which have never been identified as alanine identity determinants (Figure 8B).

Global data covering the conserved ‘identity positions’ in the three domains of life are summarized in Table 5. Several features are easily identifiable. (i) The number of conserved positions (nucleotides, base pairs and base-triples) in a given tRNA family is surprisingly low, with only two positions in the tRNA^Thr^ family and at most eight positions in the tRNA^Asp^ family. (ii) Conserved identity positions in each of the 20 tRNA families always include anticodon positions. These positions are always found to be of medium importance and often correspond to experimentally proven strong aminoacylation determinants, meaning that additionally they could code for tRNA properties outside aminoacylation. (iii) About 59% of conserved identity positions relate to determinants for tRNA aminoacylation that are already validated in some of the presently 36 investigated model taxa (Supplementary Table S1). Only 13% map with positions outside the anticodon and mostly in tRNA families recognized by class II aaRSs. (iv) Only ∼13% of conserved positions show medium ‘relative entropies’. Altogether, and as expected, this highlights the importance of anticodons and the anticodon-encrypted genetic code in all extant tRNAs. It also shows the princeps status of tRNA aminoacylation determinants and suggests a primordial role for aaRSs in this process. (v) Significantly different distributions of conserved positions in the tRNA families agree with the functional and structural differences in class I and class II tRNA/aaRS systems. (vi) Many conserved positions sustain either specific tRNA conformation or presently uncharacterized new identities, in particular in tRNA families recognized by class Ib or class IIb aaRSs.

**Table 5.** Importance of conserved ‘identity positions’ in tRNAs from the three domains of life, as measured by relative Eentropy according to (385)

tRNA families	Nucleotides with important identity positions
**Arg**	4–69; ***20***; *36*
**Cys**	*3*–*70*; 13–22; *34*; *35*; *36*
**Ile**	29–41; *34*; *35*; *36*
**Leu**	12–23; 35; 36
**Met**	*31*–*39*; *34*; *35*; *36*
**Val**	*35*; *36*; *73*
**Glu**	11–24; 12–23; 13, *34*; *35*; 36
**Gln**	12–23; 13–22; *34*; *35*; *38*; 44; 73
**Trp**	31–39; *34*; *35*; *36*
**Tyr**	12–23; *34*; *35*; 36
**Ala**	*2*–*71*; *3*–***70***; *4*–*69*; 20a; **35**; **36**
**Gly**	*2*–*71*; *3*–*70*; 31–39; 35; 36
**His**	2–71; 32; 34; *35*; *36*; 38
**Pro**	*2*–*71*; *35*; *36*; *37*
**Ser**	13–22; 35; 36; 46; 47; *73*
**Thr**	*35*; *36*
**Asp**	11–24; 20a; *25*; 31–39; *34*; *35*; *36*; ***73***
**Asn**	2–71; 31–39; ***34***; *35*; *36*; 51–63; ***73***
**Lys**	12–23; 34; 35; 36
**Phe**	12–23; *20a*; *34*; *35*; *36*; *73*

tRNA families refer to the ensemble of isoacceptors and isodecoders specific to a given proteinogenic amino acid. Data are displayed according to the ranking of aaRSs in two classes, each subdivided into three subclasses. Relative entropy (RE) values are shown in regular black (RE 0.22–0.4) and bold black (RE ∼0.4–0.7). Note that RE values >0.7 are found in a few tRNA families from specific domains of life (see Supplementary Tables S3–S5). Positions of anticodons are underlined. Positions in italics are occupied in some taxa by validated determinants for tRNA aminoacylation.

More specific characteristics emerge when analyzing the predicted ‘identity positions’ in tRNAs from individual domains of life. In this case, the number of conserved positions in most tRNA families increases significantly, with up to 21, 25 and 22 positions in bacterial, eukaryal and archaeal tRNAs, respectively. This trend mainly concerns the tRNA^Met^, tRNA^Glu^, tRNA^Asp^ and tRNA^Phe^ families (Supplementary Tables S3–S5). However, in a few families, this number remains low, with only 3–7 important positions, notably three in the bacterial tRNA^Val^ families and four in the eukaryal tRNA^Thr^ families. Finally, the variable distributions of predictions in the three domains of life associated with tRNA conformations or identity determinants support the particular evolutionary status of *Archaea* and of several tRNA families, notably the valine and threonine families that show well-differentiated importance profiles in the three domains of life.

#### A simplified global search

In this approach, the complexity of the problem was simplified by restricting the search to sequences of tRNAs whose lengths match the canonical cloverleaf (discarding the extra loop in the variable region and the 3′-CCA_OH_ terminus). Overall ∼13 000 gene sequences retrieved from a curated database were analyzed by measuring information variations (374,384). The result of the study was the prediction of clusters of tRNA positions defining each specificity. A cluster contains a number of nucleotides whose presence is coordinated. In other words, each nucleotide present in a site is derived by the presence of a nucleotide found in another site of the cluster. In each cluster, the search differentiates the anticodon, Watson–Crick and non-Watson–Crick positions. The number of clusters for tRNAs recognized by class I aaRSs ranges from one (Arg, Met and Trp), two (Glu, Leu and Val), three (Gln), seven (Cys and Ile) and up to 13 (Tyr). For class II specificities, clusters range from two (Ala and Phe), three (Gly, Lys and Thr), four (Asp and His), five (Asn and Ser) to six (Pro) predicted identity positions. In each cluster, the number of positions varies from two to 13/14 in the clusters defining Cys, Ile or Tyr identities. Interestingly, in four large clusters (Arg, Gln, Ile and Leu), positions 8–14 and 54–58 (tertiary pairs) occur with anticodon position 35, an association that never occurs in clusters for class II aaRSs. In both classes, positions from the T loop are often associated with the anticodon. Except for anticodons that are always predicted, only a few base pairs correspond to conserved positions in the three domains of life, such as C_3_–G_70_ in 55% of tRNA^Cys^, or base pair 11–24 that is always Y_11_–R_24_ for Ile and often U_11_–A_24_ for Asp, in agreement with other results (385). Altogether, anticodon positions and positions associated with tertiary interactions are highlighted, but other predictions, such as the variable size of clusters, remain unexplained.

#### Other global comparative searches

Further tRNA searches in the three domains of life were performed using the Leipzig database (164) and the archaeal split tRNA databases (375), and on sequences specific to some phylogenetic groups (383). First, and as expected, positions in anticodon loops and at the top of the acceptor stems are predicted in *Bacteria* and *Eukarya*, with the highest probability in *Bacteria* (388). More interesting, positions 30–40 and 31–39 in the anticodon branch are predicted in several tRNAs as determinants, e.g. the pair U_31_–A_39_ in *E. coli* tRNA^Trp^ and *S. cerevisiae* tRNA^Met^ and the pairs U_30_·G_40_ and G_30_·U_40_ in eukaryal tRNAs^Ile^ and *S. cerevisiae* tRNA^Asp^, respectively (383,388). With the exception of pair 30–40 in *S. cerevisiae* tRNA^Asp^ slightly shifted from the predicted 31–39 pair, these findings are consistent with previous studies using a Bayesian search (385).

Secondly, identity clusters were searched in sequences of comparable length, omitting the variable region (389). Many of these clusters are related to position 35 of the anticodons. Furthermore, in *Archaea*, positions at the 5′ end of the acceptor stem are present in all clusters (except Leu). In *Bacteria*, position 8 is present in clusters for Ala, Arg, Gln, Gly, Thr, Trp and Val, and in *Eukarya* for Gln, Leu, Met, Phe, Ser, Trp, Tyr and Val, but never in *Archaea*. More generally, archaeal tRNAs possess the broadest spectrum of predicted identity elements, followed by eukaryal tRNAs. Some of the predicted positions are occupied by validated aminoacylation identity elements (e.g. the G_3_·U_70_ in tRNA^Ala^ is part of a cluster in the three domains of life). In addition, the G_10_·U_25_ base pair in *S. cerevisiae* tRNA^Asp^ is either part of an eukaryal cluster or is associated with post-transcriptional modifications. Position 15, present in archaeal clusters, is probably associated with the identity of archaeosine tRNA guanine transglycosylase, the enzyme that inserts archaeosine derivatives into almost all archaeal tRNAs (390). However, the status of many positions (e.g. in the T branch) remains unknown and is probably associated with other tRNA functions.

#### Focused searches in individual kingdoms or specific tRNAs or derivatives

An extensive search for positions coding identity information in *Archaea* was performed on ∼4000 archaeal tRNA gene sequences from 86 species (instead of 22 previously) (386). The aim was to discover a minimal operational RNA code (moRNA) generalizing the operational RNA code for amino acids (10). The analysis was conducted on 43 potential informative positions outside the anticodon (since the anticodons alone perfectly predict the identity of the tRNAs). The six most informative positions are, respectively, G_72_, C_73_, U_47_, U_73_, A_20_ and G_20_ in the genes of tRNA^Tyr^, tRNA^His^, tRNA^Leu^, tRNA^Ala^, tRNA^Arg^ and tRNA^Phe^ (according to decreasing importance). These six positions are weakly predicted in other archaeal tRNAs, such as tRNA^Ile^ or tRNA^Val^. Interestingly, many of these statistically predicted positions were already known as archaeal identity elements for aminoacylation, for example in tRNA^Leu^ (55), tRNA^Tyr^ (104), tRNA^His^ (143) and tRNA^Phe^ (195). On the other hand, distinct phyla have different moRNA codes, suggesting that moRNA codes have evolved during speciation.

Other searches for identity determinants in mt-tRNAs have been performed in the ∼5000 mt-tRNA genes compiled in the Leipzig and Mamit databases (164,376). For example, the A_14_ and A_73_ determinants of human mt-tRNA^Leu(UUR)^ were found to be conserved in mammals (284). The A_73_ determinants and the GUC anticodon of human mt-tRNA^Asp^ are conserved in primates but only partially in other mammalian families (293) (e.g. the GUC anticodon replaced by GCC in marsupials and A_73_ absent in insects but conserved in marsupials). The case of human mt-tRNA^Ala^ is notable for the shift of the determinant G_3_·U_70_ to position 5–68. The shift is found in primates, but only partially in other *Mammalia* and *Eukarya* that show a weak preference for G_4_·C_69_ wobble pairs. This results from sequence constraints relaxed in the mt-tRNAs^Ala^ of eumetazoans (286). In the human mt-tRNA^Phe^, the G_1_–C_73_ and A_73_ determinants are conserved in mammals but only partially in lower phyla (298,299).

Further focused searches on several hundred non-metazoan organisms largely confirmed the importance of A_20_ in the tRNA^Arg^ identity, but revealed notable exceptions in Stramenopiles and Diatoms, where A_20_ is replaced by C_20_ or U_20_ in most cytosolic tRNAs, whereas it is conserved in mitochondria and plastids (391).

The recent search for sequence covariations in viral genomic RNAs, complemented by biochemical studies, has expanded the world of viral TLSs (247,248). As for TLSs^Val^, 108 examples of *in vitro* valylatable TLSs were found in plant-infecting viruses and in some insect-infecting tetraviruses. Except for their 7 nt anticodon loops (with fully conserved A_35_CAC_38_) and their well-conserved anticodon stems, these molecules show great structural heterogeneity. In a few cases, the insertion of large stem–loops into the TLS core does not prevent valylation, and in one case an anticodon distant from the valine anticodon prevents valylation. In contrast to the canonical tRNAs^Val^ where the discriminator base A_73_ is conserved and acts as a universal identity determinant, the discriminator of TLSs^Val^ is not conserved, being A_73_ or C_73_ (247). However, the strong valine identity element A_35_ in the anticodon of tRNA^Val^ is present in all TLSs^Val^ and is complemented by C_36_ as characterized in TYMV TLS^Val^ aminoacylated by wheat germ ValRS (392). Similarly, many new examples of histidinylatable TLSs^His^ have been added to the previously described TLSs^His^ (222). Like other viral TLSs, their sequences are organized in a consensus secondary structure in a pseudoknotted secondary structure (Figure 4C) that contains the identity elements previously characterized in tobamoviral TLS^His^, including the atypical pair N_−1_–N_73_ and G_34_, U_35_ of the His anticodon (248).

#### Search of structural features in tRNA and tRNA:aaRS complexes related to tRNA identity

##### Evolutionary conservation of interacting regions

The binding surfaces between tRNAs and their respective aaRSs were studied in 16 crystal structures of tRNA:aaRS complexes, mainly from *Bacteria* and *Eukarya* (393). This interaction information was analyzed considering a sequence conservation analysis carried out on tRNA sequences of ∼400 organisms evenly distributed in the three domains of life. This allowed the identification of interaction regions in the tRNA:aaRS complexes in the three domains of life and the evolutionarily conserved ribonucleotides in the tRNA molecules (393). The resulting common features and heterogeneity between the tRNA and tRNA:aaRS complexes can be summarized as follows. (i) Three regions (anticodon loop, -CCA_OH_ terminal region, followed by D stem) and two additional loop regions (D and T loops) involved in the formation of the tRNA L-shape are largely conserved. (ii) In tRNA:aaRS complexes, the regions of tRNA that interact most with aaRSs are the 3′-CCA_OH_ and the anticodon loop, except in the bacterial Leu and Ser complexes. (iii) The 5′ half of tRNAs contains more interacting nucleotide than the 3′ half. (iv) In *Bacteria*, tRNA:aaRS complexes classified according to similarities in their tRNA interaction patterns have led to six classes. They occur in a mosaic pattern, in which the tRNA:aaRS complexes corresponding to each class are intermingled, suggesting that variations in the interaction characteristics between tRNAs and aaRSs are not always dependent on the aaRS class (393).

##### Structural signatures in tRNAs related with aminoacylation identity

Despite the large number of tRNA genes found in eumetazoans, specific tRNA sequence motifs are highly conserved and often span one or more sets of isoacceptors and isodecoders (394). This is the case for non-Watson–Crick base pairs in helical stems, notably G·U pairs and non-isosteric U·G pairs. The G_10_·U_25_ pair in the D stem is found in five families of tRNA isoacceptors, including tRNA^Asp^. The G_10_·U_25_ pair has been shown to participate indirectly in the aspartylation identity of *S. cerevisiae* tRNA^Asp^ (184,327). Similarly, the U_30_·G_40_ pair is found in the anticodon stem of mammalian and insect tRNAs^Ile^ and other isoacceptors (394). The U_30_·G_40_ pair may be conserved because it interacts with the ribosome during translocation. For example, the suppression efficiency of the yeast amber tRNA^Ile^ in *E. coli* is modulated by the presence of the U_30_·G_40_ pair and is accompanied by an identity change revealing a different mode of recognition of the anticodon nucleotides (209). Similarly, although mutagenesis of the G_30_–U_40_ pair in the anticodon stem of *S. cerevisiae* tRNA^Asp^ only marginally affects aspartylation (184), it results in a deviation of the anticodon arm and a conformational change in the anticodon loop required for recognition of identity elements by AspRS (315,326).

##### Primordial identities in ancestral systems

Early studies suggested that the two classes of primordial aaRSs or proto-aaRSs might originally be encoded by complementary strands of the same nucleic acid (395). Functional class I and II amino acid-activating peptides encoded by opposite strands of the same gene have been characterized more recently (396). This is a first step towards the characterization of proto-aaRSs with aminoacylation activity. This raises questions about the mechanism of recognition of ancestral tRNAs by proto-aaRSs. Regression methods have identified possible sequence motifs in modern aaRSs that are able to perform this discrimination. As a result, groove recognition rules have emerged based on the differential thermodynamic stability of tRNA helical -NCCA_OH_ extensions (397).

##### Coding triplets in tRNA acceptor stems

The relationship between tRNA aminoacylation and the genetic code embedded in the tRNA acceptor arm has recently been re-investigated. A large-scale analysis of bacterial tRNA sequences revealed that for six amino acids (Ala, Asp, Gly, His, Pro and Ser), mainly those considered to be the oldest, the tRNA acceptor arm contains the corresponding coding triplets well beyond the statistical expectation (398). As relics and early identity elements, these coding triplets are suggested to have a primordial origin, being involved in the aminoacylation of pre-biotic tRNAs (399) and the establishment of the canonical codon set in agreement with the RNA operational code theory (10).

#### The unexplored identities of tRNA-derived fragments

A completely unexplored aspect in the emerging field of tRF (tRNA-derived RNA fragment) biology is the search for identity signatures for the recognition of the macromolecular partners interacting with these RNAs (224). These tRFs present in the three domains of life regulate many cellular processes, e.g. stress and immune responses, crosstalk with ribosomes, reverse transcriptases, tRNA modification enzymes and aaRSs. Recent work on tRF regulation of ribosome-associated aaRSs in *S. cerevisiae* provides information for a better understanding of identity signatures in tRNAs (225). Five tRFs [3′-tRF^His(GUG)^, 3′-tRF^Ser(AGA)^, 3′-tRF^Leu(UAA)^, 3′-tRF_1_^Thr(UGU)^ and 5′-tRF^His(GUG)^] interact with their cognate yeast aaRSs and impact tRNA aminoacylation. Interestingly, 3′-tRF^Ser(AGA)^ includes part of the long variable arm important for serine identity, and both 3′-tRF^Leu(UAA)^ and 3′-tRF_1_^Thr(UGU)^ include, respectively, A_73_ and G_71_C_72_ known as identity determinants of aminoacylation in eukaryal tRNAs (Table 1). Such observations should stimulate further studies of tRFs, in particular to deconvolute the encrypted identity signatures in tRNAs.

### tRNA identity for other tRNA-binding proteins

Fewer studies have been devoted to the study of identity elements governing tRNA biosynthesis or other tRNA functions. However, their number is increasing, starting with studies that focus on the identity elements of tRNA-modifying enzymes. For example, the G_10_–U_25_ base pair in the D arm was found as a major identity determinant for formation of m^2^_2_G_26_ or m^2^_2_G_10_ by archaeal *Pyrococcus abyssi* m^2^G transferases (400). In *S. cerevisiae*, the essential elements required for G_10_ methylation by the Trm11–Trm12 complex are the terminal A_76_ and the G_10_–C_25_ base pair in tRNAs with a variable region of regular size (e.g. tRNAs specific for Arg, Asn, Ile, Leu, Lys, Met, Phe, Thr, Trp, Tyr and Val). In addition, U_38_ in tRNA^Ala^ and the U_32_–A_38_ base pair in tRNA^Cys^ are negative identity elements against this methylation (401). For t^6^A_37_ biosynthesis (e.g. in tRNA^Ile^ or tRNA^Thr^), C_32_ and the D stem are essential determinants for the modification machinery (known as KEOPS complexes) in yeast, humans and nematodes (40). TrmL is the prokaryotic methyltransferase that catalyzes the 2′-*O*-methylation of base 34 of the isoacceptors tRNA^Leu(CAA)^ and tRNA^Leu(UAA)^. The anticodon loop of these tRNAs is critical for TrmL recognition. Nucleotide A_35_ is a key determinant, as is the A_36_A_37_A_38_ motif, which additionally requires the presence of the prior isopentenylation (i^6^) of A_37_. Only pyrimidine nucleotides at position 34 are substrates of TrmL (402). In another example, the human tRNA methylase DNMT2/TRDMT1 was shown to catalyze formation of m^5^C_38_ of several tRNAs thanks to a conserved identity sequence C_32_UNNCAC_38_ found in the anticodon loop (403).

Formylation of the tRNA^Met^ initiator is another form of post-transcriptional modification. The formyl group is present in *Prokarya* and in organelles (chloroplasts and mitochondria) but not in *Eukarya*. Formylation is not dependent on the side chain of the esterified amino acid, and several misaminoacylated tRNAs have been shown to be formylatable, suggesting that the nucleotide sequence of the tRNA rather than the esterified amino acid carries the formylation identity. Indeed, the identity elements for methionyl-tRNA transformylase are clustered in the acceptor stem with a major role for the unpaired pair C_1_–A_72_ assisted by the minor determinants A_73_, G_2_–C_71_, C_3_–G_70_ and G_4_–C_69_ (404–406). Acetylation of the amino acid charged on the tRNA is another post-transcriptional modification. Acetylation occurs on the glycine charged on tRNA^Gly^ by TacT toxin of *Salmonella typhimurium*. The identity of the acetylation activity is specified by U_73_ and G_71_, a combination of nucleotides found only in tRNA^Gly^ isoacceptors (407).

In addition to their role in protein biosynthesis, aminoacyl-tRNAs are found to participate in various biochemical processes, such as cell wall formation, protein labeling for degradation, aminoacylation of cell membrane phospholipids or synthesis of peptides with antibiotic properties (408). For the synthesis of the peptidoglycan of the bacterial cell wall, there is a plethora of enzymes depending on the bacterium and the wall to be synthesized. Various amino acids are ligated to the peptidoglycan, but glycine, alanine and serine are the most frequently incorporated from the corresponding aminoacylated tRNAs. These tRNAs can be dedicated to synthesis of peptidoglycans. For example, there are three non-proteinogenic tRNAs^Gly^ in *Staphylococcus aureus*, in which sequence elements have been replaced to escape EF-Tu. The lost identity elements for EF-Tu include base pairs 49–65, 51–63, 49–65 and 51–63 in the T loop. The new determinants for these enzymes remain to be discovered (409,410).

To resist cationic antimicrobial peptides, many *Bacteria* have developed resistance mechanisms through MprF proteins that aminoacylate anionic phospholipids with l-lysine or l-alanine. The presence of positive charges on the membrane surface reduces the affinity for cationic antimicrobial peptides. How MprF and similar enzymes divert aminoacyl-tRNAs to membrane lipid modification remains an open question, as these tRNAs seem to have the same affinity for EF-Tu and MprF (411). The specificity of MprF was proposed to arise from direct recognition of the aminoacyl moiety of the aminoacyl-tRNA (411).

The aminoacyl-tRNAs also serve as amino acid donors in the synthetic pathways of significantly different compounds with antibiotic properties (412), including valanimycin, pacidamycin and cyclodipeptides (413). The identity elements for the enzymes responsible for these transformations are still unknown. Aminoacyl-tRNAs also serve as amino acid donors in protein degradation pathways through aminoacyl-tRNA-protein transferases, which recognize a secondary destabilizing residue at the N-terminus of proteins and attach a primary destabilizing residue. In *Eukarya*, this added residue is the arginine bound by the arginyl(R)-transferase. In *Prokarya*, leucine and phenylalanine are the primary destabilizing N-terminal residues for leucyl/phenylalanyl(L/F)-transferases. Aminoacyl-tRNA-protein transferases specifically recognize the aminoacyl moiety of aminoacyl-tRNAs and the constant unpaired CCA_76_ nucleotides of their acceptor ends (414,415).

The tRNAs also serve as substrates for various processing enzymes. RNase P and Z mature the 5′ and 3′ ends of primary transcripts regardless of their sequences. Similarly, tRNA nucleotidyltransferase adds the CCA end to all tRNAs whatever their identity and sequence. The tRFs are produced by cleavage in the anticodon loop by angiogenin or colicin nucleases. The removal of introns from *Archaea* and *Eukarya* tRNAs is orchestrated by several enzymatic activities. The process depends little on conserved sequence-specific recognition and is primarily based on proximity and base pair interactions between the intron and the tRNA body to form the proper structure for cleavage (416).

As exposed above, most *Bacteria* and *Archaea* do not possess AsnRS and/or GlnRS, and some methanogenic archaea do not possess CysRS. Furthermore, no aaRS for the rare amino acid selenocysteine has been found in any domain of life. Instead, these organisms use indirect pathways to synthesize these amino acids (Asn, Cys, Gln and Sec) directly on their corresponding tRNAs. After an initial step in which non-discriminating aaRSs form misacylated aminoacylated tRNAs, they are converted to the corresponding aminoacyl-tRNAs by various RNA-dependent modification enzymes (GatCAB, GatDE and SepCysS) that specifically recognize the corresponding identity determinants. How these misacylated aminoacyl-tRNAs are recognized by the modifying enzymes and diverted from the translation machinery, where they could be toxic, has been studied and is described in the preceding sections.

Together, the different enzymes that interact with tRNAs imply the presence on a given tRNA of distinct or overlapping sets of identities. By analogy with recognition by aaRSs, indirect reading of structural elements on tRNAs could also be used. The search for these new identity elements could benefit from the large-scale bioinformatics analyses currently being carried out in the three domains of life (385,386,389).

## HUMAN DISEASES RESULTING FROM DISORDERS IN tRNA IDENTITY

Mutations in mt-tRNAs have been known for years to cause human diseases (417). More recently, cytoplasmic tRNA variants have also been identified (418,419). The toxicity of these disease-causing mutations often has pleiotropic effects that affect all stages from biogenesis, structure and function of tRNAs (420).

**Figure 9.**
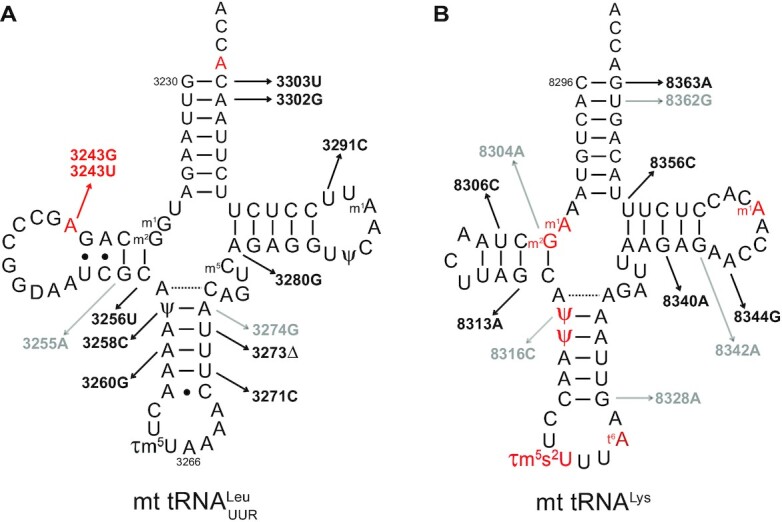
Pathogenic human mt-tRNAs, with most known mutations highlighted. (**A**) mt-tRNA^Leu(UUR)^ and (**B**) mt-tRNA^Lys^ cloverleaves with base modifications indicated. The location of mutations is indicated by arrows (black arrows for mutations with confirmed pathogenetic status, gray arrows for mutations of ‘likely pathogenic’ status). Red arrows highlight confirmed pathogenic mutations affecting identity elements. Data and status are from MITOMAP.

The number of diseases linked to dysfunction in human tRNAs is increasing. Diseases due to mutations in mt-tRNAs cover diverse pathologies, such as cardiopathies and neuropathies, with a wealth of clinical manifestations (277). A largely ignored cause of diseases relies on perturbed expression of genes encoding many tRNA modification enzymes, leading to aberrant tissue-specific profiles of tRNA modifications (421). Diseases, named ‘modopathies’, are associated with aberrant tRNA modifications (422). Today >350 mutations in human mt-tRNAs are compiled in the MITOMAP (http://www.mitomap.org) databases; among them about half are pathogenic (423). Their discovery, prediction, evolution and penetrance (presence in other organisms) result from a plethora of theoretical, experimental and clinical studies (424). Pathogenic mutations occur in the 22 human mt-tRNAs, but with uneven distributions. The two most common (A3243G and A8344G), which account for a majority of diseases, are expressed in mt-tRNA^Leu(UUR)^ and mt-tRNA^Lys^ (Figure 9).

In general, pathogenic mutations are mild, minimizing the risk of lethality, and rarely affect major identity positions in the tRNA. Most of the time, they are located in the vicinity of the positions occupied by the major identity determinants and often lead to mismatches in the helical regions that destabilize the conformations recognized by the aaRSs. Six pathogenic mutations leading to mismatches occur in the four stems of mt-tRNA^Ala^ (425), but only two (C_6_U and C_69_U) lead to G·U mismatches in the acceptor stem and are related to alanine identity (426). Thus, pathogeny is due to mutation of minor alanine identity elements.

Diseases associated with aminoacylation identity affect five other tRNA specificities (Ile, Leu, Asp, Ser and Lys). In mt-tRNA^Ile^, the two isoleucine determinants A_7_ and A_59_ in the acceptor stem and T loop, found in two cardiopathy-causing variants with G_7_ and G_59_, are not located at the positions of known identity determinants and act by conformational effects (300). In well-studied human mt-tRNA^Leu(UUR)^, three disease-causing mutations are associated with MELAS syndrome (Mitochondrial Encephalomyopathy, Lactic Acidosis and Stroke-like episodes). These mutations either disrupt the conserved U_8_:A_14_ reverse Hoogsteen base pair or are associated with a perturbed base triple (G_13_–C_22_):G_46_ and a fragile anticodon stem constituted of four A–U pairs and a mismatched A_41_·C_39_ pair closing the 7 nt anticodon loop (Figure 9A). Mutations affect both the tRNA leucylation efficiency and the tRNA structure. Interestingly, this fragile anticodon stem participates in leucine identity. Collectively, this indicates that the pathogenicity of mt-tRNA^Leu(UUR)^ is sensitive to perturbed structural identity determinants and anticodon stem structure (284). In human mt-tRNA^Asp^, the A_9_G mutation in the D stem, associated with myopathy, disrupts the triple interaction A_9_:A_12_–U_23_ and reduces aspartylation efficiency. Therefore, both A_9_ and A_9_:A_12_–U_23_ may participate in aspartate identity (294). In mt-tRNA^Ser(UCN)^, the G_31_A mutation associated with polycystic ovary syndrome and insulin resistance disrupts a G·U pair at the top of the anticodon stem and alters aminoacylation (427). In mt-tRNA^Lys^ (Figure 9B), the absence of m^1^ methylation at A_9_ in the core of the tRNA impairs cloverleaf folding and alters aminoacylation (240). Finally, nine mitochondrial ‘modopathies’ affect nucleotides undergoing modification (428). Aberrant modification patterns (i.e. at i^6^A, m^1^A, Ψ, m^1^G, m^2^_2_G, f^5^C, m^5^C and hypermodified U positions in anticodon loops) alter various steps of tRNA biology, including aminoacylation (240,300) and codon reading (203). Similarly, hypomodifications in pathogenic cytoplasmic tRNAs are common, particularly in the tRNA core and in the anticodon loops (i.e. at the positions mchm^5^U_34_, ms^2^t^6^A_37_ and yW_37_) (422). This probably perturbs tRNA conformations, impacting tRNA aminoacylation identity and/or codon reading on the ribosome.

Emerging evidence points to an increasing role for tRNA mutations in genetic disorders, cancer and neurological diseases. In this context, ∼600 pathogenic mutations have been identified in human tRNA genes, with consequences for tRNA function, mRNA translation and proteome composition. For example, a mutation at C_65_ in the extended acceptor stem of human tRNA^Sec^ causes a complex phenotype of neurological disorders due to reduced expression of the tRNA and decreased 2**′**-*O*-methylribosylation at mcm^5^U_34_ in the anticodon of mutant tRNA^Sec^ (418). As the C_65_ mutation occurs in one of the three base pairs that constitute identity elements for SelB, the elongation factor for selenoprotein translation in *E. coli*, it is tempting to propose that a disruption in recognition of the mammalian SelB homolog explains pathology (418).

## CONCLUSION

### A critical look at the methodologies used in identity studies

The determination of identity elements in tRNAs remains currently a technological issue that is not yet fully solved. Early approaches to constructing modified tRNAs by fragment ligation were complex and time consuming, e.g. (66). The use of suppressor tRNAs has proven to be an improvement due to its greater ease of implementation and the cellular context in which competition between aaRSs is ubiquitous, e.g. (51). However, the approach remains limited for some identities that use nucleotides in their anticodons that differ from those present in suppressor tRNAs. The synthesis of *in vitro* transcribed tRNA variants has been by far the most widely used (193). However, it is limited by the absence of modified bases, which can sometimes play a decisive role in identity. Modified bases may be involved in stabilizing the tertiary structure of tRNAs and thus play a role in indirect reading by ensuring the positioning of identity elements. In addition, standard nucleotide substitutions result in multiple atomic group changes relative to the wild-type base, leading to steric effects or repulsive effects. Interactions with the aaRS may disappear or be redistributed and generate amplifications or attenuations of the primary effects. Another drawback of the method is that aminoacylation assays are often performed *in vitro* with purified enzymes outside the context of competition, under experimental conditions that may be far from the cellular environment. Transplantation of the identified identity elements into another tRNA core is additional evidence that validates the identification, although it is also subject to some caveats due to the use of a different tRNA backbone.

Alternatively, mutagenesis of the side chains of aaRS residues that interact with identity elements can be used to remove interactions with identity elements (typically by Ala mutagenesis). Studies on well-known model systems have shown that loss of interactions involved in identity generally induces relaxed tRNA specificity with decreases in *k*_cat_ in the presence of competing non-cognate tRNAs but not in the presence of the pure cognate tRNA. This loss of discrimination against non-cognate tRNAs revealed by this approach allows for a more certain identification of interactions involved in identity (215,308).

Another method not yet employed would be to create abasic tRNAs at defined positions that would allow precise measurement of the effect of the loss of a given interaction [inspired by the molecular surgery already used in the case of tRNA^Ala^ (126,316,429)]. Although molecular tools are still lacking to create such mutants, abasic sites are already known, for example in 28S rRNA where the base A_4323_ is removed by ricin (430). Abasic sites are also found in tRNAs where certain bases are labile, such as wybutosine (Y or yW) at position 37 in tRNA^Phe^ (431), as well as the modified base m^7^G at position 46 in other tRNAs (432). Interestingly, removal of yW_37_ significantly impairs phenylalanine acceptance (433). It can be expected that in the near future new tools will be available. The recent characterization of methylpurine DNA glycosylase, which generates abasic RNAs on RNA–DNA hybrids (434), may become programmable in the future, opening up exciting prospects in the search for tRNA identity elements.

### Future directions for tRNA identity research

Over the past two decades, the field of tRNAs has entered a new era in which the prototypical concept of tRNA identity has been challenged and progressively refined in response to new discoveries. The increasing importance of auxiliary protein factors alongside aaRSs in tRNA biology is significant.

This has produced a paradigm shift, with semantic changes illustrated by the concepts of ‘quality control’ underlying tRNA aminoacylation (361) and of ‘code biology’ (435) extending the ‘operational RNA code’ (10). More generally, tRNA is a pivotal marker in evolution, illustrated by breakthroughs in eukaryotic tRNA biology (2,3). On the other hand, large-scale searches of tRNA sequences by bioinformatics methods revealed patterns of importance of tRNA positions related to identities and suggested that ‘almost nothing in most tRNA positions is even close to random’ (385). Therefore, the long-term prospect will be to decipher globally in given tRNAs the functional role of nucleotides at these positions.

What are the most immediate prospects? Only a few aminoacylation identities are known in organelles, and the field is completely open for apicoplast and chloroplast tRNAs. Furthermore, it is crucial to better understand identities outside of aminoacylation, especially for tRNA maturation and splicing. To do this, known and predicted determinants must be deconvoluted in terms of aminoacylation and associated functions. In addition, much remains to be discovered in tRNA biology, including its dynamic regulation under specific environmental and ecological conditions. In particular, how the cellular tRNA pool varies in response to various metabolic contexts is poorly understood and needs to be quantified. Epistasic effects that cover combined effects of mild mutations in tRNAs and/or aaRSs are other challenges (387,436). To achieve these ambitious goals, appropriate biochemical, genetic, computational and deep sequencing tools are needed. Finally, it is becoming clear that the identity of tRNAs recapitulates the evolutionary history of protein synthesis and life, and this puzzle must be clarified to understand the origins of tRNAs.

## DATA AVAILABILITY

All data are available from the authors upon request.

## Supplementary Material

gkad007_Supplemental_File

## Appendix

### Definitions

Catalytic efficiency: defined by the *k*_cat_/*K*_M_ ratio of aminoacylation reactions, with *k*_cat_ the catalytic rate constant and *K*_M_ the Michaelis constant representing an approximation of the inverse of tRNA affinity for an aaRS, so that ‘L’ = (*k*_cat_/*K*_M_)_native_/(*k*_cat_/*K*_M_)_mutant_ (with ‘L’ representing the loss of catalytic efficiency).

Classes of tRNAs: based on the presence of a small variable region of 4/5 nt (class I) or a large variable region with an extra loop (class II).

Classes and subclasses of aaRSs: defined based on the structure and peptide motifs present in their catalytic sites.

Identity determinant: nucleoside or chemical groups on a nucleoside, as well as structural elements (mainly in tRNAs) essential for biological activity (in proteins, this concerns amino acids).

Identity set: a set of key determinants for specific activities.

Identity rules: account for the operational functioning of identity determinants in taxonomic groups (originally they applied exclusively to the aminoacylation reaction of tRNAs and were considered ‘universal’ if they were conserved in the three domains of life).

Isoacceptor tRNAs: tRNAs that accept the same amino acid and may have different anticodons due to genetic code degeneracy.

Isodecoder tRNAs: tRNAs with the same anticodon sequence that decode the same codon. They belong to the same class of isoacceptors but have sequence differences elsewhere in the tRNA body.

Strength of aminoacylation identity determinants: measured *in vitro* by losses of catalytic efficiency of aminoacylation reactions of tRNA upon mutation at identity positions. Alternatively, strength can be measured *in vivo* by *E. coli* suppressor genetics that utilizes a reporter dihydrofolate reductase gene with the amber mutational position 10.

Tertiary interactions in tRNA: interactions that stabilize the structure of tRNA. They are mainly located in the core of the tRNA molecule and include, for example, interactions between the D stem and variable loop, T loop and anticodon loop, and U_8_ with A_14_.

tRNA domains: D stem/loops from N_10_ to N_25_ with loops from A_14_ to A_21_ divided into α and β subdomains (sizes from 2 to 4 nts) located down- and upstream of the constant G_18_ and G_19_); anticodon stem/loops from N_27_ to N_43_ with 7 nt loops (N_32_–N_38_); variable regions (VR) from N_44_ to Y_48_ with smallVRs (4 or 5 nt) or largeVRs (also named extra arm) (up to 16 nt from positions 47a to 47p); T stem/loops from N_47_ to N_65_ with 7 nt loops (T_54_–N_60_); connecting Ns: U_8_N_9_ and N_26_.
