# Supplementary material for: The tRNA identity landscape for aminoacylation and beyond
Source: Nucleic Acids Res. 2023 Feb 6;51(4):1528–70. doi: 10.1093/nar/gkad007 (PMC9976931; doi:10.1093/nar/gkad007)
Supplement: gkad007_Supplemental_File [file gkad007_supplemental_file.pdf]

Supplementary data and Tables to

The tRNA identity landscape for aminoacylation and beyond

by

Richard Giegé and Gilbert Eriani

**Supplementary Table 1** Organisms covering the 3 domains of life in which identity determinants were experimentally validated, including eukaryal organelles	**Page 2**
**Supplementary Table 2** Positive identity elements of aminoacylation experimentally validated in mt-tRNAs and api-$tRNA^{Tyr}$	**Page 3**
**Supplementary Table 3** Importance of identity positions in bacterial tRNAs determined by Relative Entropy calculation	**Page 4**
**Supplementary Table 4** Importance of identity positions in eukaryal tRNAs determined by Relative Entropy calculation	**Page 5**
**Supplementary Table 5** Importance of identity positions in archaeal tRNAs determined by Relative Entropy calculation	**Page 6**

Supplementary Table 1 Organisms in the 3 domains of life in which identity determinants have been experimentally validated, including eukaryotic organelles.

Domains of Life		
Bacteria	***Eukarya***	***Archaea***
Bacillus stearothermophilus[a]<br>Proteobacteria/Geobacilli	***Saccharomyces cerevisiae*** (C,O)<br>Fungi	***Aeropyrum pernix***<br>Crenarchaeota
Caulibacter crescentus<br>Proteobacteria	*Pneumocystic carinii* (C)<br>Fungi	*Haloferax volcanii*<br>Euryarchaeota
Escherichia coli<br>Proteobacteria	*Acanthamoeba castellanii* (C)<br>Protozoa	***Methanocaldococcus jannaschii***<br>Euryarchaeota
Haemophilus influenzae<br>Proteobacteria	*Plasmodium falciparum* (O)<br>Protozoa	*Methanococcus maripaludis*<br>Euryarchaeota
Helicobacter pylori<br>Proteobacteria	*Trypanosoma brucei* (C)<br>Protozoa	*Methanosarcina mazei*<br>Euryarchaeota
Bacillus subtilis<br>Firmicute	*Arabidopsis thaliana* (C)<br>Plantae	*Methanosarcina barkeri*<br>Euryarchaeota
Bacillus mori<br>Firmicute	*Orysa sativa* (O)<br>Plantae	*Methanohalarchaeum thermophilus*<br>Euryarchaeota
Lactobacilli (plantarum, casei)<br>Firmicute	Zea mays (C)<br>Plantae	
Borrelia burgdorferi<br>Spirochaeta	Tobacco Mosaic Virus (C*)<br>Plantae (Solanaceae)	
Aquifex aeolicus<br>Aquificia	Turnip Yellow Mosaic Virus (C*)<br>Plantae (Brassicacea)	
Thermotoga maritima<br>Thermotoga	several *Porifera & Arthropoda* (O)<br>Animalia	
Thermus thermophilus<br>Deinococci	*Ascaris suum (O)*<br>Animalia (Nematoda)	
	Caenorhabtidis elegans (O)<br>Animalia (Nematoda)	
	Parachtes romandiolae<br>Animalia (Spiders)	
	Carryedes brasiliensis (O)<br>Animalia (Insecta)	
	Drosophila melanogaster<br>Animalia (Insecta)	
	Monodelphis domestica (O)<br>Animalia (Mammalia)	
	several *Rodentia* (Rattus, hamster) (C)<br>Animalia (Mammalia)	
	Homo sapiens (C;O)<br>Animalia (Mammalia)	

A variety of taxa (here with their kingdom classification) have been studied, but only a few taxa (in bold) were studied extensively revealing determinants for a wide range of tRNA specificities. [a] Renamed *Geobacillus stearothermophilus*. Determinants in eukayal cytosol (C), in organelles (O) and at the 3' terminus of plant RNA viruses (C*)

Supplementary Table 2 Positive identity elements of aminoacylation experimentally validated in mt-tRNAs and api-$tRNA^{Tyr}$.

tRNA	mt-tRNAs and api-$tRNA^{Tyr}$ domains		
**families**	**Acceptor branch**	**Core region**	**Anticodon branch**
**Arg**	…	…	U_{34}, G_{36}
**Ile** [c]	A_7	A_{59}	…
**Leu**	A_{73}	A_{14}	…
**Met**	…	m^1A_9	…
**Trp**	G_{73}, G_1–C_{72}, U_5–A_{68}	…	…
**Tyr**	G_1–C_{72}	long variable region [a]	G_{34} [a], U_{35} [a]
**Ala**	G•U [d]	…	…
**Gly**	…	…	C_{35}
**His**	G_{-1}	…	…
**Ser**	…	A_{57} [b], A_{58} [b]	…
**Asp**	…	A_9:A_{12}–U_{23}	G_{34}, U_{35}, C_{34}
**Lys**	…	m^1A	…
**Phe**	A_{73}, G_1–C_{72}	m^1A	…

The tRNA families are displayed according to the class of their cognate aaRSs. The position of the determinants in the sequence of the tRNA acceptor branch, the core region, and the anticodon branch is shown with standard numbering. Footnotes: [a] api-tRNAs. [b] truncated $tRNA^{Ser(UGA)}$; [c] 5 modified nts (m^1G_9, $m^2{}_2G_{26}$, Y_{27}, Y_{28}, t^6A_{37}) contribute collectively to identity; [d] the pair G_3•U_{70} can be translocated in the acceptor stem (see text); … , no data available.

Supplementary Table 3 Importance of identity positions in bacterial tRNAs determined by Relative Entropy calculation. Adapted from Branciamore *et al.* (2018) Intrinsic properties of tRNA molecules as deciphered via Bayesian network and distribution divergence analysis. *Life (Basel)* **8**, E5.

tRNA families	*Bacteria*
**Arg**	*4-69*; 5-68; 20; 20a; *35*; *36*; 73
**Cys**	*3-70*; 12-23; *13-22*; 17; 29-41; *34*; *35*; *36*; 43; 45; 46; 51-63; 71; 73
**Ile**	6-67; 12-23; 13-22; 20a; 27-43; 28-42; *29-41*; *34*; *35*; *36*; 44; 51-63
**Leu**	2-71; 9; 15; 21; *12-23*; *13-22*; *20a*; 21; *35*; *36*; 44; 46; 47; *EA*; 73
**Met$_{ini}$**	***1**-72*; *2-71*; 3-70; 5-68; 6-67; **11-24**; 12-23; 17a; 26; *27-43*; 29-41; *31-39*; 32; *34*; *35*; *36*; 44; 57; 59; 73
**Met**	*31-39*; *34*; *35*; *36*; 71; 73
**Val**	*35*; *36*; 73
**Gln**	**1-72**; *12-23*; *13-22*; 20a; *34*; *35*; *36*; 38; *44*; 45; *46*; 51-63; 65; *73*
**Glu**	*3-70*; 4-69; 5-68; 7-66; 9; *11-24*; *12-23*; *13*; 17; 20a; 30-40; *34*; *35*; *36*; 38; 45; 46; *47*; 49-65; 51-63; 71
**Trp**	1-72; 3-70; 20a; 29-41; *31-39*; *34*; *35*; *36*; 73
**Tyr**	6-67; 10-25; *12-23*; 17; 20; 20a; **20b**; *34*; *35*; *36*; 44; EA; 59; 71; 73
**Ala**	*2-71*; *3-**70***; *4-69*; 17; 20; *20a*; *35*; *36*; 44; 64; 73
**Gly**	*2-71*; *3-70*; 29-41; *31-39*; *35*; *36*; 63; **73**
**His**	**-1**; *2-71*; 3-70; 4-69; 31-39; *32*; *34*; *35*; *36*; *38*; **73**
**Pro**	**1-72**; *2-71*; 3-70; 17a; *35*; *36*; *37*; 44; 73
**Ser**	2-71; 3-70; 12-23; *13-22*; 20a; **20b**; *35*; *36*; *46*; *47*; *EA*; 59; *73*
**Thr**	2-71; 3-70; 20a; *35*; *36*
**Asp**	*11-24*; 20a; *25*; *31-39*; *34*; *35*; *36*; 38; 43; 44; 50-64; 51-63; 65; *73*
**Asn**	1-72; *2-71*; 3-70; 12-23; *31-39*; 32; *34*; *35*; *36*; *51-63*; 73
**Lys**	4-69; 5-68; 7-66; *12-23*; 20a; *31-39*; *34*; *35*; *36*; 37; 73?
**Phe**	3-70; *12-23*; 17; *20a*; 27-43; 31-39; *34*; *35*; *36*; 39; 43; 44; 45; 51-63; 59; *73*

The tRNA families refer to the set of isoacceptor and isodecoder specific to a given amino acid. The importance of identity positions is measured by RE (Relative Entropy) values and is displayed in black scripts (RE 0.22–0.4), red scripts (RE ~0.4–0.7) and **red bold scripts** (RE ~0.7 and above). The positions occupied in some taxa by confirmed aminoacylation determinants (including the entire extra arm 'EA' in a few tRNA subclasses), are underlined. Positions predicted as important in tRNAs from the three Domains of Life are in italics.

Specific comments: (i) High RE's above 0.7 (in the Gln, Tyr, Ala, Gly, His, Pro, and Ser tRNA families) correspond to positions occupied by validated determinants, except positions 20b in the Tyr and Ser families. (ii) Note the conservation, for example, of $G_3 \bullet U_{70}$ in $tRNA^{Ala}$ and $C_3–G_{70}$ in $tRNA^{Cys}$. (iii) Discriminator position 73 is predicted in 18 tRNA families, except in the Glu and Thr families.

Supplementary Table 4 Importance of identity positions in eukaryal tRNAs determined by Relative Entropy calculation. Adapted from Branciamore *et al.* (2018) Intrinsic properties of tRNA molecules as deciphered via Bayesian network and distribution divergence analysis. *Life (Basel)* **8**, E5.

tRNA Families	*Eukarya*
**Arg**	*4-69*; 15; 20; *35*; *36*; 48; 71
**Cys**	2-71; *3-70*; 12-23; *13-22*; *34*; *35*; *36*; 51-63; 59; 68; 69
**Ile**	4-69; 17; 20a; 28-42; *29-41*; 30-40; *34*; *35*; *36*; 60
**Leu**	9; *12-23*; *13-22*; *20a*; 20b; *35*; *36*; 37; 44; 45; 47; *EA*; 49-65; 68
**Met$_{ini}$**	*1-72*; *2-71*; *3-70*; 4-69; 5-68; 6-67; 7-66; 12-23; 20; 22; 27-43; 29-41; 31-39; 33; *34*; *35*; *36*; 38; 46; 51-63; 54; 60; 73
**Met**	1-72; 12-23; 20; *31-39*; *34*; *35*; *36*; 60; 64
**Val**	2-71; 3-70; 5-68; 11-24; 12-23; 13-22; 27-43; 31-39; *35*; *36*; 38; 60
**Gln**	6-67; 7-66; *12-23*; *13-22*; 26; 29-41; 31-39; *34*; *35*; *36*; *44*; *46*; 47; 52-62; *73*
**Glu**	1-72; 2-71; *3-70*; 5-68; *11-24*; *12-23*; 13-22; 26; 31-39; *34*; *35*; *36*; 38; 47; 59
**Trp**	2-71; 15; 20a; *31-39*; *34*; *35*; *36*; 43; 48; 52-62; 65
**Tyr**	1-72; *12-23*; 13-22; 27-43; 27-43; 28-42; 31-39; *34*; *35*; *36*; 51-63; 70
**Ala**	*2-71*; ***3-70***; *4-69*; 5-68; 9; 12-23;13-22; 17; *20a*; 27-43; 31-39; 32; *35*; *36*; 38; 59
**Gly**	*2-71*; *3-70*; 11-24; 25; *31-39*; *35*; *36*; 47; 59; **73**
**His**	*2-71*; 9; 11-24; 12-23; 26; 30-40; 31-39; *32*; *34*; *35*; *36*; 37; *38*; 44; 46; 47
**Pro**	*2-71*; 11-24; 12-23; 13-22; 20a; 25; 26; 27-43; 29-41; 31-39; 32; *35*; *36*; *37*; 38; 49-65; *73*
**Ser**	4-69; 5-68; *13-22*; 49-65; 59; *35*; *36*; 44; *46*; *47*; *EA*; 51-63; 73
**Thr**	31-39; *35*; *36*; 73
**Asp**	1-72; 9-12-23; *11-24*; 12-23; 13-22; *25*; 29-41; 20a; 25; 26; 28-42; *31-39*; *34*; *35*; *36*; 38; 43; 46; 47; 49-65; 50-64; 59; 63; 72; *73*
**Asn**	*2-71*; 4-69; 13-22; 17; 27-43; 29-41; *31-39*; *34*; *35*; *36*; 38; 49-65; 50-64; *51-63*; 59; 73
**Lys**	2-71; 7-66; 9; *12-23*; 13-22; 17; 20a; 29-41; *31-39*; *34*; *35*; *36*; 44; 59; 70
**Phe**	2-71; 4-69; 5-68; 6-67; 9; *12-23*; 13-22; 17; 20; *20a*; 28-42; 29-41; 31-39; *34*; *35*; *36*; 37; 51-63; 59; 60; *73*

The tRNA families refer to the set of isoacceptor and isodecoder specific to a given amino acid. The importance of identity positions is measured by RE (Relative Entropy) values and is displayed in black scripts (RE 0.22–0.4), red scripts (RE ~0.4–0.7) and **red bold scripts** (RE ~0.7 and above). The positions occupied in some taxa by confirmed aminoacylation determinants (including the entire extra arm 'EA' in a few tRNA subclasses), are underlined. Positions predicted as important in tRNAs from the three Domains of Life are in italics.

Specific comments: Several features are obvious, notably: (i) Position 70 occupied by U (part of the identity determinant C_3•U_{70}) in the Ala family shows by far the highest RE indicator (>0.7). (ii) In contrast, all other important positions are marked by medium or low RE indicators. (iii) Positions occupied by experimentally proven aminoacylation identity determinants are the most abundant in the Gly, Asp, and Phe families.

Supplementary Table 5 Importance of identity positions in archaeal tRNAs determined by Relative Entropy calculation. Adapted from Branciamore *et al.* (2018) Intrinsic properties of tRNA molecules as deciphered via Bayesian network and distribution divergence analysis. *Life (Basel)* **8**, E5.

**tRNA Families**	***Archaea***
**Arg**	*4-69*; 20; *35*; *36*; 73
**Cys**	*3-70*; 4-69; 5-68; *13-22*; 17; 17a; 20; 21; 24; 27-43; *34*; *35*; *36*; 45; 73
**Ile**	2-71; 11-24; 12-23; *29-41*; *34*; *35*; *36*; 31-39; 37; 46; 73
**Leu**	2-71; 3-70; 4-69; 5-68; *12-23*; *13-22*; *20a*; 20b; 31-39; *35*; *36*; 37; 44; 46; *EA*
**Met_{ini}**	*1-72*; *2-71*; 9; 11-24; 17; 17a; 20; 20b; *27-43*; *31-39*; *34*; *35*; *36*; 37; 47; 51-63; 57; 73
**Met**	*31-39*; *34*; *35*; *36*; 37; 73
**Val**	3-70; 4-69; 5-68; 6-67; 11-24; 12-23; 20a; 30-40; 31-39; *35*; *36*; 47; 73
**Gln**	**1-72**; 4-69; *12-23*; *13-22*; 17; 17a; 20a; 20b; 25; *34*; *35*; *36*; 37; *44*; *46*; 49; *73*
**Glu**	2-71; *3-70*; *11-24*; *12-23*; *13*-22; 17a; 20a; 20b; *34*; *35*; *36*; *47*; 46; 49-65
**Trp**	2-71; 3-70; 6-67; 22; 27-43; *31-39*; *34*; *35*; *36*; 48; 50-64
**Tyr**	1-72; 4-69; 9; *12-23*; 13-22; 17; 27-43; 28-42; 31-39; *34*; *35*; *36*; 46; 47; 51-63
**Ala**	*2-71*; *3-**70***; *4-69*; 9; 12-23; 13-22; *20a*; *35*; *36*; 30-40; 44; 47
**Gly**	*2-71*; *3-70*; 11-24; *31-39*; *35*; *36*; 13-22; 49-65
**His**	**-1**; *2-71*; 3-70; 5-68; 11-24; 12-23; *32*; *34*; *35*; *36*; 37; *38*; 50-64; 63; 73
**Pro**	*2-71*; 3-70; 6-67; 11-24; 12-23; 13-22; 17a; 25; *35*; *36*; 37; 46; 59
**Ser**	2-71; 4-69; 5-68; 12-23; *13-22*; 20a; 22; 24; *35*; *36*; 44; *46*; *47*; *EA*; 51-63; 73
**Thr**	2-71; 3-70; 11-24; 12-23; *35*; *36*; 37; 46; 73
**Asp**	2-71; 3-70; 6-67; *11-24*; 12-23; 13-22; 17a; 20; 20a; 20b; *25*; 28-42; *31-39*; *34*; *35*; *36*; 44; 46; 47; 49-65; 64; *73*
**Asn**	*2-71*; 3-70; 9; 11-24; 12-23; 17; 20a; 22; *31-39*; 34; 35; 36; 37; 46; 47; *51-63*; 59; 73
**Lys**	2-71; 3-70; 4-69; 9; 11-24; *12-23*; 22; *31-39*; *34*; *35*; *36*; 37; 46; 73
**Phe**	9; *12-23*; 13-22; 20; *20a*; *34*; *35*; *36*; 37; 45; 46; 47; *73*

The tRNA families refer to the set of isoacceptor and isodecoder specific to a given amino acid. The importance of identity positions is measured by RE (Relative Entropy) values and is displayed in black scripts (RE 0.22–0.4), red scripts (RE ~0.4–0.7) and **red bold scripts** (RE ~0.7 and above). The positions occupied in some taxa by confirmed aminoacylation determinants (including the entire extra arm 'EA' in a few tRNA subclasses), are underlined. Positions predicted as important in tRNAs from the three Domains of Life are in italics.

Specific comments: The results show similarities with the predicted positions in eukaryotic tRNAs. For instance, the RE indicators are low, except at position 70 in the Ala family and only few additional positions outside anticodon are occupied by validated determinants.
